# A survival of the fittest strategy for the selection of genotypes by which drug responders and non-responders can be predicted in small groups

**DOI:** 10.1371/journal.pone.0246828

**Published:** 2021-03-05

**Authors:** Daniël Höhle, Kim van Rooij, Jos Bloemers, James G. Pfaus, Frits Michiels, Paddy Janssen, Eric Claassen, Adriaan Tuiten

**Affiliations:** 1 Emotional Brain B.V., Almere, The Netherlands; 2 Utrecht Institute for Pharmaceutical Sciences and Rudolf Magnus Institute of Neuroscience, Utrecht University, Utrecht, The Netherlands; 3 Centro de Investigaciones Cerebrales, Xalapa, Mexico; 4 Chemistry and Life Sciences, V.O. Patients & Trademarks, Amsterdam, The Netherlands; 5 Department of Clinical Pharmacy and Toxicology, Maastricht University Medical Center+, Maastricht, The Netherlands; 6 Department of Hospital Pharmacy, VieCuri Medical Center Venlo, Venlo, The Netherlands; 7 Athena Institute, Faculty of Science, Vrije Universiteit Amsterdam, Amsterdam, The Netherlands; UNITED STATES

## Abstract

Phenotype Prediction Scores (PPS) might be powerful tools to predict traits or the efficacy of treatments based on combinations of Single-Nucleotide Polymorphism *(*SNPs) in large samples. We developed a novel method to produce PPS models for small samples sizes. The set of SNPs is first filtered on those known to be relevant in biological pathways involved in a clinical condition, and then further filtered repeatedly in a survival strategy to select stabile positive/negative risk alleles. This method is applied on Female Sexual Interest/Arousal Disorder (FSIAD), for which two subtypes has been proposed: 1) a relatively insensitive excitatory system in the brain for sexual cues, and 2) a dysfunctional activation of brain mechanisms for sexual inhibition. A double-blind, randomized, placebo-controlled cross-over experiment was conducted on 129 women with FSIAD. The women received three different on-demand drug-combination treatments during 3 two-week periods: testosterone (0.5 mg) + sildenafil (50 mg), testosterone (0.5 mg) + buspirone (10 mg), or matching placebos. The resulted PPS were independently validated on patient-level and group-level. The AUC scores for T+S of the derivation set was 0.867 (95% CI = 0.796–0.939; p<0.001) and was 0.890 (95% CI = 0.778–1.000; p<0.001) on the validation set. For T+B the AUC of the derivation set was 0.957 (95% CI = 0.921–0.992; p<0.001) and 0.869 (95% CI = 0.746–0.992; p<0.001) for the validation set. Both formulas could reliably predict for each drug who benefit from the on-demand drugs and could therefore be useful in clinical practice.

## Introduction

The main symptoms of the diagnoses Female Sexual Interest/Arousal Disorder (FSIAD) [1] are low sexual desire and sexual arousal. This condition negatively influences psychological well-being and results in personal distress [2]. Although FSIAD is a common condition which is prevalent among women of all ages and ethnicities, little is known about its etiology.

Attempts to develop a drug treatment for FSIAD have been guided by the principle of "one size fits all" and have failed to acknowledge the complexity of female sexuality. Sex and its problems form an integral part of human existence, evoking strong emotional responses and fierce debates among scientists and in the public domain. Each message in the media about drug development for FSIAD raises a schism: nurture against nature, seldom the combination of the two. In our search for personalized medicines for FSIAD, we embraced the idea that people acclimate to sexual experiences and adjust their expectations accordingly. Thus, by the time they seek help, they have lived with low desire and/or arousal for a relatively long time. Biological constitution forms a foundation upon which experiences either raise or diminish expectations. For women with FSIAD, two biological constraints underlie desire and arousal problems: 1) A relatively insensitive system for sexual cues, and 2) dysfunctional activation of sexual inhibitory mechanisms. Hence, we differentiated two subtypes of women with FSIAD (i.e. two phenotypes for which the involvement of different genes are assumed), for which we developed two on-demand drugs that addressed these two mechanisms specifically.

In a phase 2 program in which these treatments were successfully tested, a demarcation formula (a combination of biological and psychological variables) was developed to distinguish the two groups before the start of the experiments [3]. However, the regulatory authorities European Medicines Agency (EMA) and Food and Drug Administration (FDA) assessed this demarcation formula as being too complicated and time consuming for practical use in the clinical setting. In response to these concerns we initially developed and tested one single Phenotype Prediction Score (PPS) based on Single Nucleotide Polymorphisms (SNPs) (using an external measure: a questionnaire focused on etiology), to predict potential responders to one drug compared to the other [4]. However, our hypothesis regarding the FSIAD subdivisions of low-sensitive versus high-inhibition (for which the two different drug treatments were developed) is not yet recognized as part of the clinical diagnosis in the Diagnostic and Statistical Manual of Mental Disorders (DSM). Therefore, the FDA recommended to test both drugs separately in an all-comer FSIAD population, enabling the use of a drug-specific SNP-based demarcation formula leading to greater effectiveness in the predicted direction [5].

Here we describe the development of two separate SNP-based PPS demarcation formulae (*without* the involvement of an external measure, such as questionnaires), to predict drug responders and non-responders separately for each drug. Our novel approach to the genetic subtyping of FSIAD patients, resulted in two PPSs (Companion Demarcation formulae–demarcating responders from non-responders in each drug), based on: 1) selection of genes that affect excitatory and inhibitory neurochemical systems known to be involved in the regulation of female sexual behavior [6, 7]; 2) Selection of SNPs associated with these genes (see for comparable hypothesis and genetic-driven selections of SNPs [8–10]); 3) A selection strategy which is best described as survival of the fittest (most stable) alleles; 4) Calculation of cumulative effect of multiple selected alleles, resulting in the final PPS, per drug.

For the development (and validation in an independent sample) of the SNP-based allocation formulae, we conducted a clinical experiment, in which women with FSIAD (n = 144) were subjected to both drugs and placebo. The PPS formulae were constructed using a series of recodings and filtering procedures in order to select the most stable alleles.

## Methods

Our manuscript does not describe the clinical trial results. This has already been done in the journal Women’s Health (https://doi.org/10.1177/1745506518788970), according to CONSORT guidelines. The present manuscript describes a different, novel method of making a group-division in our study sample. The manuscript’s goal is to explain how this method was designed.

### Study participants

**Fig 1:**
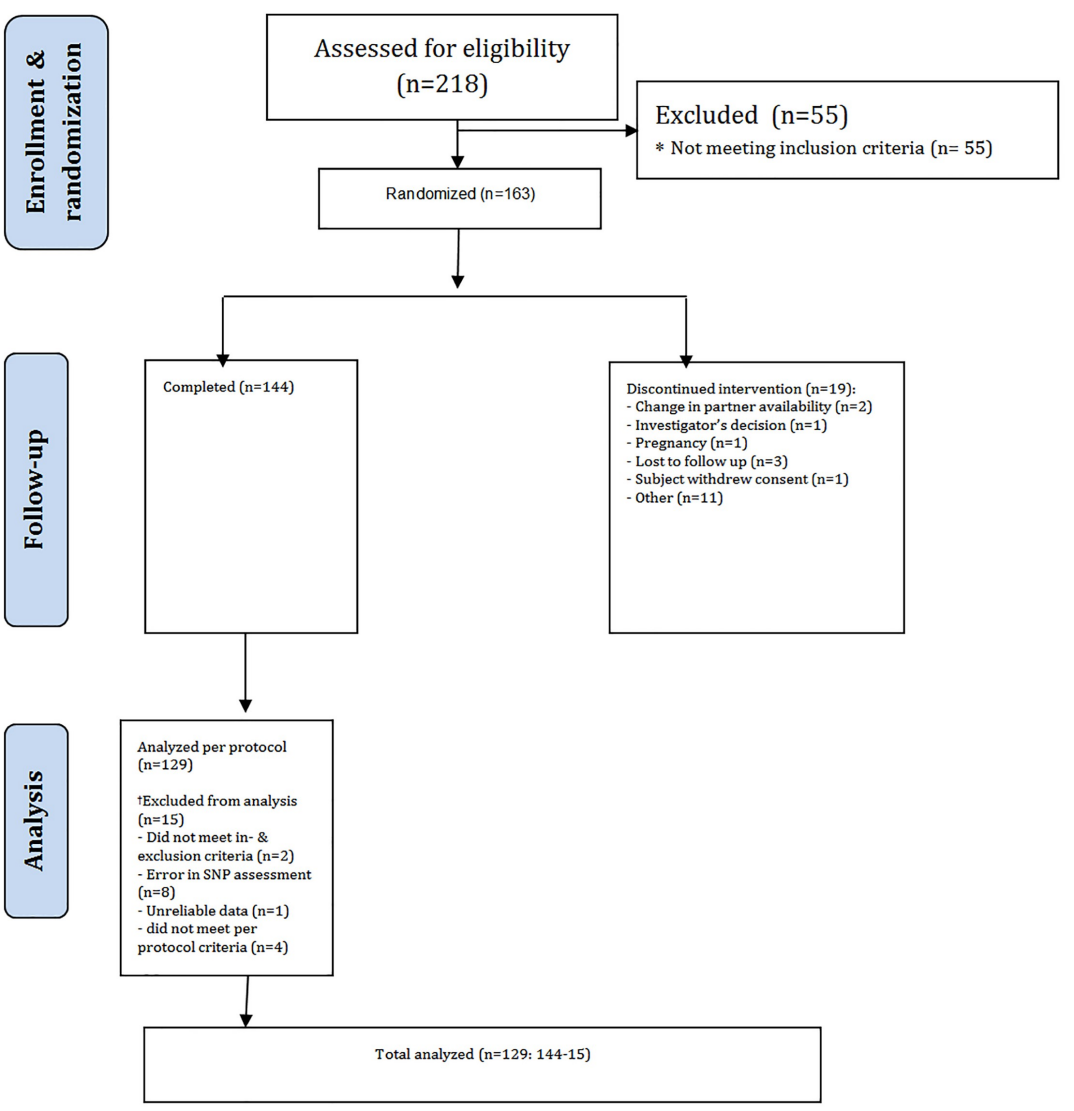
Consort diagram.

Women between the ages of 18 and 70 were recruited via advertisements and a volunteer database of Emotional Brain BV (Almere, The Netherlands). Enrollment took place between 7 February 2014 (first screening visit) and 12 August 2014 (last follow-up visit, as per protocol). 218 women were screened after providing written informed consent. A trained psychologist diagnosed FSIAD according to the Diagnostic and Statistical Manual for Mental Disorders, edition 5 (DSM-5) [1]. Female orgasmic disorder as a secondary diagnosis was allowed. Medical history was recorded, a physical examination including a 12-lead electrocardiogram, and a urine pregnancy test were performed. Standard biochemistry, serology and hematological laboratory parameters were assessed. Baseline levels of total testosterone, sex hormone-binding globulin, albumin, thyroid-stimulating hormone, follicle-stimulating hormone, luteinizing hormone, prolactin, and estrogen, were also assessed at screening. Exclusionary criteria included: not being in a stable and communicative monogamous relationship; having a sexually dysfunctional partner; abnormal medical history; pregnancy; and abnormal medical markers as determined during the physical examination, laboratory values, and vital signs (see **Fig 1**). Subject demographics are summarized in **Table 1**.

**Table 1:** Demographics.

	Number of Subjects (%)
Parameter	Total (N = 129)
**Age (Category)**	
< 40	84 (65.1)
40–60	41 (31.8)
≥ 60	4 (3.1)
**Age (Years)**	
Mean	34.3
Minimum	18.0
Maximum	64.0
**Body Mass Index**	
< 35	128 (99.2)
≥ 35	1 (0.8)
**Menopausal status**	
Post-menopausal	22 (17.1)
Pre-menopausal	107 (82.9)
**Race**	
Caucasian	119 (92.2)
Black	2 (1.6)
Asian	3 (2.3)
Other	5 (3.9)

Note: Denominator for the calculation of percentages: total number of subjects that meet per protocol analysis.

### Study execution and approval

The study was conducted at two clinical research units of Emotional Brain BV (Almere and Utrecht, The Netherlands) by trained research personnel, and was carried out in agreement with the Declaration of Helsinki (October 2008) and the International Conference on Harmonization—Good Clinical Practice guidelines for clinical research. It was approved by Medical Ethics Committee ‘Stichting BEBO’ (Assen, The Netherlands) and the Dutch Competent Authority (Centrale Commissie Mensgebonden Onderzoek), authorization number NL44803.056.13. It was registered in the European Clinical Trials Database, EudraCT number 2011-000457-23, under Primary Registry trial number (Nederlands Trial Register) NTR4426 (30/01/2014) and was monitored by PSR Group (Hoofddorp, The Netherlands).

### Study design

This randomized, double-blind, placebo-controlled, cross-over study included a 2-week single-blind placebo run-in period (PRI) and three 2-week double-blind treatment periods (placebo, testosterone + silfenafil [T+S], testosterone + buspirone [T+B]), followed by a 1-week follow-up period. Each regimen was separated by at least one 2-day washout period (both drugs are intended for on-demand use; systemic clearance takes approximately 24 hours). Participants’ treatment sequence was based on a randomization list generated by independent statisticians of Pharma Consulting Group (Uppsala, Sweden), according to a 6-sequence Williams design. Participants were randomized using an interactive web-response system, integral to the electronic case report form (Viedoc™, version 3.22; Pharma Consulting Group, Uppsala, Sweden). No study participants, no research personnel, and no sponsor personnel had access to the randomization list before database lock.

Participants visited the study site 7 times: 1 screening visit, 1 startup visit, 4 regimen follow-up visits, and 1 final follow-up visit. During the start-up visit and regimen follow-up visits, sexual functioning was evaluated, health was monitored, and study medication was dispensed. At the start-up visit, blood was drawn for the assessment of the SNPs.

#### Primary endpoint

The primary endpoint was the change in the number of satisfactory sexual events (SSEs) after medication intake between the single-blind PRI and the double-blind active treatment period, as measured by the Sexual Event Diary (SED) [11, 12]. For derivation and validation of the formulae, the primary endpoint was used as the outcome measure. For a complete description of the SED and the data capture and processing of change in SSE, see [4].

#### Medication and dosing

See Supplementary Methods for rationale behind and technical information on the investigational pharmaceutical products.

**Sublingual testosterone + sildenafil (T+S)**: a dual-route/dual-release fixed-dose combination of testosterone and sildenafil citrate [13], intended for on/demand use.

**Sublingual testosterone + buspirone (T+B)**: a dual-route/dual-release fixed-dose combination of testosterone and buspirone hydrochloride [14], intended for on/demand use.

**Placebo**: Placebo tablets had the exact same appearance and flavor as the fixed-dose combination T+S and T+B tablets. All medication was manufactured and packaged at Piramal Healthcare UK (Morpeth, UK).

**Dosing**: Participants were instructed to keep the tablet under their tongue for 90 seconds, then to swallow the tablet as a whole, without chewing or otherwise disrupting the tablet. Participants were allowed to swallow the tablet using water. Participants were instructed to engage in sexual activity between 3 and 6 hours after ingestion. The dosing instructions were the same for all medications.

**Duration of treatment**: A total of 8 doses per regime were provided. Participants were instructed to try to take a minimum of 4 doses during the 2-week treatment periods (2 doses/week). The other 4 doses could be taken as desired (i.e., “on demand”) throughout the 2-week treatment period; dosing was permitted every 2 days (i.e., on alternate days).

#### SNP analysis

Deoxyribonucleic acid (DNA) extraction was performed by Medigenomix (Ebersberg, Germany). SNP analysis was performed using a Microarray chip (HumanCytoSNP-12 bead chip, Illumina, containing 297 622 SNPs). The hybridization and chip readout were outsourced to expert labs Eurofins (The Netherlands)/AROS Applied Biotechnology (Aarhus, Denmark).

#### A priori selection of SNPs based on theoretical arguments

We selected SNPs known to be in genes of interest. Based on literature, genes related to sexual functioning and which might be involved in FSIAD and/or might be affected by T+S or T+B were selected. Cross-referencing with genes represented on the chip resulted in an a priori selection of 285 SNPs including: the dopamine signaling pathway (dopamine receptor genes D2 and D3 [DRD2 and DRD3], solute carrier family 6 member 3 [SLC6A3] dopamine transporter gene, dopamine beta hydroxylase [DBH] and monoamine oxidase B [MAOB] genes, and the adenosine A2A (ADORA2A) receptor gene; serotonin [5-HT] signaling pathway (HTR1A, HTR1B, HTR1E, HTR1F, HTR2A, HTR2B, HTR2C, HTR3B, HTR3C, HTR4 and HTR7 receptor genes and the SLC6A4 serotonin transporter gene); norepinephrine pathway (solute carrier family 6 member 2 [SLC6A2] norepinephrine transporter gene and the adrenoceptor alpha 1A [ADRA1A] and adrenoceptor alpha 1B [ADRA1B] receptor genes); glucocorticoid Nuclear Receptor Subfamily 3 Group C Member 1 (NR3C1) receptor gene; melanocortin 1 receptor (MC1R) and melanocortin 2 receptor (MC2R) genes; sex hormone binding globulin (SHBG) gene; oxytocin signaling pathway (oxytocin [OXT] gene and oxytocin receptor [OXTR] gene); prolactin gene (PRL) and receptor gene (PRLR); nitric oxide signaling pathway (nitric oxide synthase [NOS1] gene); phosphodiesterase type 5 (PDE5) gene, and the estradiol receptors (ESR1 and ESR2).

### Procedural steps to derive and validate a final PRS/GRSS formula

- **Outcome Measure for Deriving polygenic risk score(PRS)/genetic risk sum score (GRSS)**

The primary endpoints were the change in number of sum of squared errors (ΔSSE). The outcome measures for the development of PRS/GRSS for each drug were dichotomizations of ΔSSE(ΔSSE_D_). Thus, when ΔSSE was 0 or lower (non-responders), the ΔSSE_D_ value was 1; when ΔSSE was 1 or higher (responders), the ΔSSE_D_ value became 2.

- **Derivation and Validation Samples**

**Fig 2:**
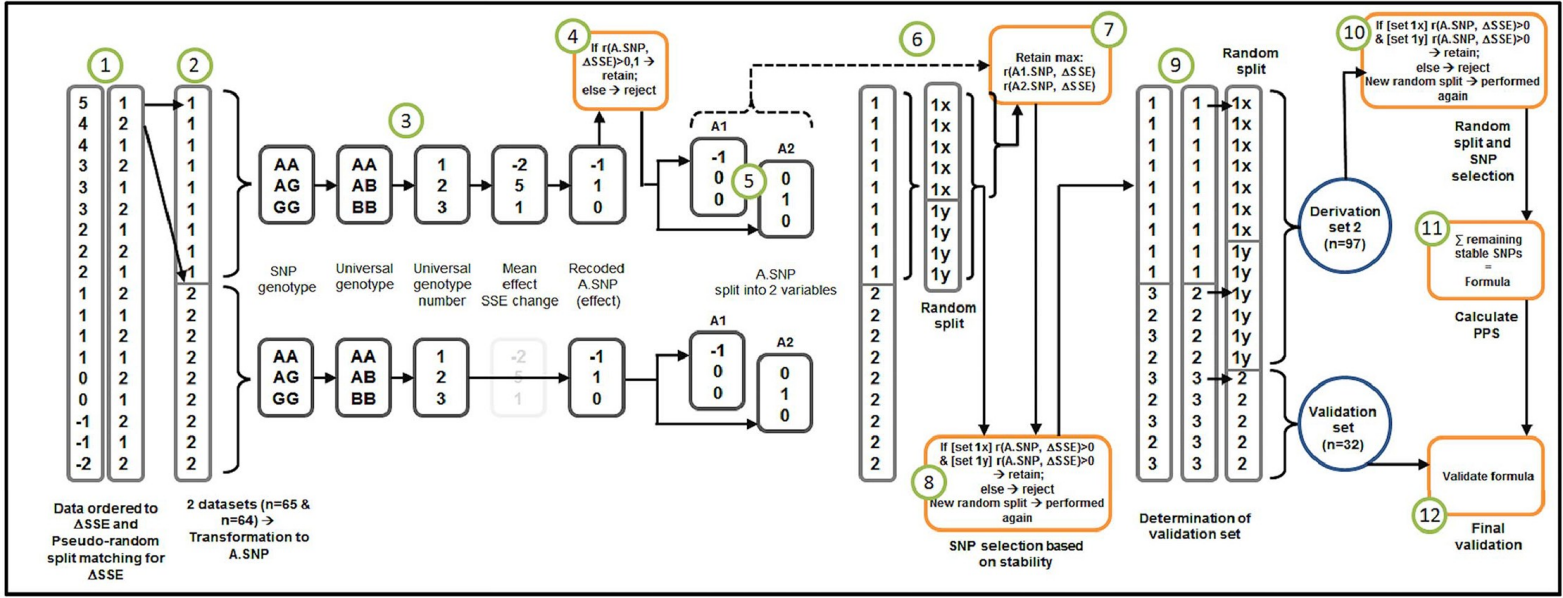
Visualization of recoding method.

For both drugs the derivation sample consisted of 97 women (75%), and the validation sample of 32 women (25%). The classification into derivation and validation samples is achieved in two consecutive steps during the procedures. The formula was validated on the patient-level and group-level. The development of the formula performed in the derivation sample is described in **Fig 2**.

#### Recoding method

From Derivation to Recodings and Accommodations of SNPs. The green-encircled numbers in the figure are referred to by the hashtag numbers here. #1] Participants were ordered according to ΔSSE. #2] the subjects were assigned a 1 (n = 65; first part of the assignment to the derivation sample) or a 2 (n = 64; intermediary set) in a pseudo-random manner, matching for number of SSEs. SNPs have the universal allelic categorical values 1, 2 and 3 (e.g. AA = 1; AC = 2; CC = 3). SNPs with less than 3 observations of an allele were excluded. Based on the first derivation sample (n = 65), ΔSSE averages were calculated for each of the alleles (of all SNPs). #3] Alleles were subsequently recoded as numerical values (i.e. according to the order of the corresponding ΔSSE average): The allele was recoded as -1 when the associated primary endpoint was lowest, as 1 when it was associated with the highest outcome value and 0 if there is an intermediary. For convenience, we call these recoded alleles: A.SNPs. We applied procedures to increase the likelihood of survival of the most consistent allele of each SNP regarding their associations with a dichotomized value of ΔSSE_D_. #4] We calculated for the first part of the assigned derivation sample (on which the recoding was based; see #2) correlations (Pearson) between the A.SNPs and ΔSSE_D_. A.SNPs with correlations lower than 0.1 were excluded. #5] The remaining A.SNPs were split into two new variables: The first only containing the negative risk alleles, with a values of -1, with the others set to 0; The second only containing the positive risk alleles, with values of 1, setting the others to 0. This split enables investigation into which of the alleles has the most influence on ΔSSE_D_. These new variables of recoded A.SNPs are hereafter referred to as A1.SNPs and A2.SNPs, for negative and positive risk alleles respectively. #6] The first part of the assigned derivation set (see #2), was randomly split in half, giving 2 sets (set 1x, n = 33; set 1y, n = 32). #7] In the 1x set, correlations were calculated for these A1 and A2.SNPs, separately, with ΔSSE_D_. The A1 or A2.SNPs with the highest correlation (per SNP) with ΔSSE_D_ survived for further analysis. #8] In the first part of the assigned derivation set (#2), the stability of the association of the alleles with ΔSSE_D_ was tested and used to select only stable alleles. This sample was randomly divided into two sets (see #6, set 1x & set 1y) and then the correlations between A1 respectively A2.SNPs with ΔSSE_D_ were calculated for these sets. If the correlation in one of the two sets was lower than 0, the associated allele was excluded from further analysis. The set was reshuffled and randomly split and this procedure was performed a second time. #9] The dataset was then restructured into the final derivation and validation set. The final derivation set consisted of the first derivation set, plus half (randomly assigned) of the intermediary set. The other half of the intermediary set would, automatically, become the validation set. This gave the derivation set (n = 97) and the validation set (n = 32). The derivation set was then randomly divided into 2 derivation subsets as before, 1x and 1y. #10] correlations between A1.SNPs and A2.SNPs with ΔSSE_D_ were calculated in both subsamples (1x and 1y). Alleles with correlations less than 0 in one of these subsamples were excluded. The set was reshuffled and randomly split again, and this procedure was performed a second time. #11] Based on the surviving “fittest” SNPs, the sum of these scores were calculated, resulting in the companion demarcation tool. #12] The tool was validated in the validation set (n = 32).

### Statistical analyses

Statistical analyses were performed using both SPSS Statistics for Windows, version 26.0 (SPSS Inc., Chicago, Ill., USA) and Statistica version 7 (TIBCO Data Sciences (formerly StatSoft), Palo Alto, CA, USA). Group-level statistics and patient-level statistics were derived to assess the efficacy, usefulness, and validity of the formulae. A Kolmogorov-Smirnov test of normality revealed that the SSE data for both T+S and T+B groups in the Placebo Run-In (PRI) phase were not normally distributed, therefore nonparametric Friedman rank-based ANOVAs (χ^2^) were conducted to assess differences between responders and non-responders in the change from PRI to T+S and to T+B for both formulae. This was assessed for the derivation sample (n = 97), validation sample (n = 32) and total sample (n = 129) of the per protocol analysis set. Effect sizes were calculated using Kendall’s coefficient of concordance (W), as defined in Kendall and Gibbons [15].

Utility at patient-level was tested by deriving a Receiver-Operating Characteristic (ROC) curve for the derivation-, validation-, and total sample (n = 129) of the per protocol analysis set. The area under the curve (AUC) of the ROC served as the test statistic for the relationship between demarcation formulae predicted and observed responder status. It was hypothesized that the formulae would perform above chance level in correctly classifying women as T+S or T+B responder/non-responder. The AUC can be interpreted as the probability that, for a given random responder–non-responder pair, the formula would produce a larger outcome for the former than for the latter. ROCs with an AUC larger than 0.714 indicate a large effect size (d’ > 0.8), and an AUC in excess of 0.80 are considered suitable for clinical use. Classification performances (accuracy, sensitivity, specificity, positive predictive value (PPV) and negative predictive value (NPV)) were calculated over all sets. These classification performance statistics are usually used to obtain information about the ability of companion diagnostic tools to identify the presence of a disease marker. In this application, our demarcation formulae were designed to classify women as T+S and T+B responder or non-responder, and these classification performances were interpreted accordingly.

## Results

In total 69 SNPs were used for the T+S formula and 65 for the T+B formula. Of these, 7 alleles overlapped from a total of 15 overlapping SNPs (see **S1 Table in S2 File**).

### ROC analyses

**Fig 3:**
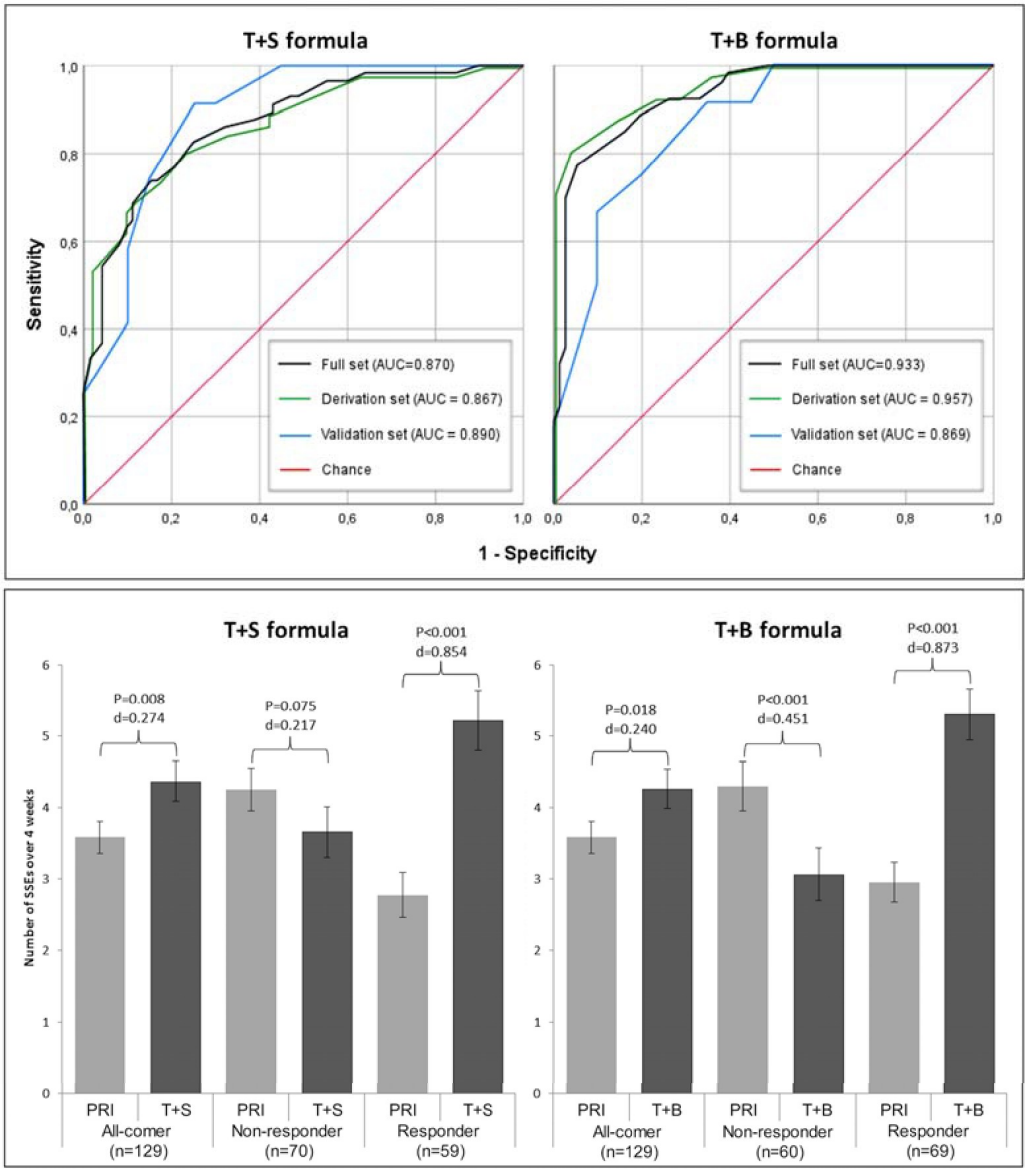
ROC. Upper panel: Receiver Operating Characteristic (ROC) curves of the derivation, validation and full sets of the T+S and T+B formulae. Lower panel: change in satisfactory sexual events (ΔSSE) from placebo run in (PRI) to testosterone + sildenafil (T+S) (left) and testosterone + buspirone (T+B) (right) active treatment period, for the entire sample (no prediction), and for the predicted T+S responders and predicted non-responders according to the T+S formula.

The ROC analyses of both formulae over all analysis sets gave AUCs of over 0.8. For the T+S formula, the AUC of the derivation set was 0.867 (95% CI = 0.796–0.939; p<0.001), for the validation set 0.890 (95% CI = 0.778–1.000; p<0.001), and for the full set 0.870 (95% CI = 0.809–0.931; p<0.001). For the T+B formula, the AUC of the derivation set was 0.957 (95% CI = 0.921–0.992; p<0.001), for the validation set 0.869 (95% CI = 0.746–0.992; p<0.001), and for the full set 0.933 (95% CI = 0.892–0.975; p<0.001). See **Fig 3** for ROC curves.

Sensitivity of the T+S formula, defined as the ability to identify T+S responders, was 0.772 over the full set. Sensitivity of the T+B formula, defined as the ability to identify T+B responders, was 0.925 over the full set. Specificity, defined as the ability of each formula to identify the respective non-responders, was 0.792 and 0.737 over the full set for the T+S and T+B formulas, respectively. PPV, defined as the proportion of correctly classified responders, was 0.746 and 0.71 over the full set for the T+S and T+B formulas, respectively. NPV, defined as the proportion of correctly classified non-responders, was 0.814 and 0.933 over the full set for the T+S and T+B formulas, respectively. Accuracy over the full sets was 0.783 and 0.814 over the full set for the T+S and T+B formulas, respectively. See **Table 2** for these classification performance statistics in the derivation and validation sets.

**Table 2:** Classification performance statistics for the T+S and T+B formulae over all analysis sets.

	T+S formula			T+B formula		
	Derivation set (n = 97)	Validation set (n = 32)	Full set (n = 129)	Derivation set (n = 97)	Validation set (n = 32)	Full set (n = 129)
Sensitivity	0.733	0.917	0.772	0.927	0.917	0.925
Specificity	0.827	0.700	0.792	0.768	0.650	0.737
PPV	0.786	0.647	0.746	0.745	0.611	0.710
NPV	0.782	0.933	0.814	0.935	0.929	0.933
Accuracy	0.784	0.781	0.783	0.835	0.750	0.814

**Notes**: PPV = positive predictive value; NPV = negative predictive value.

### Group-level analysis

In all subjects, the ΔSSE from PRI (mean = 3.58; SD = 2.55) to the T+S active treatment arm (mean = 4.37; SD = 3.17) and to the T+B active treatment arm (mean = 4.26; SD = 3.10) were significant (χ^2^ [N = 129, df = 1] = 4.74, p = 0.028, and χ^2^ [N = 129, df = 1] = 5.70, p = 0.013, respectively). Dividing the sample into T+S responders and T+S non-responders using the T+S formula resulted in a significant increase (χ^2^ [N = 57, df = 1] = 57, p<0.00001) in ΔSSE from PRI (mean = 2.78; SD = 2.41) to T+S active treatment (mean = 5.22; SD = 3.20) for the responders, and a significant decrease (χ^2^ [N = 72, df = 1] = 36, p<0.0001) from PRI (mean = 4.26; SD = 2.48) to T+S active treatment (mean = 3.66; SD = 2.99) for the non-responders. Dividing the sample into T+B responders and T+B non-responders using the T+B formula resulted in a significant increase (χ^2^ [N = 53, df = 1] = 53, p<0.00001) in ΔSSE from PRI (mean = 2.96; SD = 2.27) to T+B active treatment (mean = 5.30; SD = 2.99) for the responders, and a significant decrease (χ^2^ [N = 76, df = 1] = 36, p<0.00001) from PRI (mean = 4.30; SD = 2.68) to T+B active treatment (mean = 3.07; SD = 2.79) for the non-responders. See **Fig 3,** also for effect sizes. Calculated effect sizes of the ΔSSE from PRI to active treatments were small in the overall groups (W = 0.036 for PRI to T+S and W = 0.025 for PRI to T+B). However, after separating responders and nonresponders for each treatment using both formulae, effect sizes were much larger, with responders in both T+S and T+B groups having complete concordance (both Ws = 1.00) and non-responders having moderate concordance (T+S W = 0.50 and T+B W = 0.49).

T + S and T + B were well tolerated and no drug related serious adverse events were observed. Adverse events were consistent with the approved labelling of sildenafil (S), buspirone (B) and/or testosterone (T). Adverse events were collected according to MeDRA method (Medical Dictionary of Regulatory Activities). See [4] for a full overview of the safety data.

## Discussion

The present study demonstrates that the described novel method of selecting and combining SNPs in relation to a specified primary endpoint can be applied to small sample sizes to develop formulae that reliably predict drug responses in different groups. Moreover, the results show that the method can be applied to different datasets (i.e. the drug treatments T+S and T+B respectively).

### Polygenic methods in literature

An increasingly common method for using SNPs to make predictions on a primary endpoint is the polygenic risk sum scores. Herein, a common method for the selection of SNPs is using the p-values of the correlation of the SNPs (with primary endpoint) as threshold [16–18]. This is in line with, yet not synonymous to, selecting based on the effect size. For SNP analyses to have significant p-values, large effects are required in small sample sizes. Since our aim is to find a genotype based on a combination of SNPs, our focus is on significant p-values over the accumulation of SNPs, not individual ones. By focussing on the stability of the effect over several subgroups in the derivation sample, instead of the effect size, we expect to have reduced the chance of overfitting and increased the likelihood of generalizability to independent subgroups such as the validation sample. In each iteration, unstable variables, i.e. variables that have a negative correlation in either–or both–of the two randomly split subsets, are excluded from further analysis. This is performed four times in our method, thereby substantially reducing the risk of overfitting. This conclusion is supported by the formula’s performance in the validation set.

As described, the alleles were ordered–separately for both described treatment groups—on effect. A score of -1 for the lowest effect, +1 for the highest and 0 if there is an intermediary. The primary endpoint (ΔSSE) was dichotomized (responder, non-responders; ΔSSE_D_) because the small sample size could result in individual cases having a disproportional effect on the score if the number of SSE itself was used. The average number of SSEs for a certain subgroup could fluctuate with individual cases when the number of subjects with a certain allele was low–this could skew the results. We propose to use this average number of SSEs when the sample sizes are more substantial. These (effect) scores were used as model coefficients, contrary to using the (multiple) linear regression values [18].

### GWAS for sexual dysfunction

In classic genome-wide association studies (GWAS), individual SNPs are selected as risk factors for diseases or disorders in very large populations. The results of GWAS’s are increasingly significant and provide important insights in genome wide correlations, even in SNPs that are very far apart or even on different chromosomes [19]. Although the GWAS studies are becoming increasingly successful given the steadily increase in sample sizes [20], this approach is impractical for small data sets. Furthermore, GWAS studies focus on finding significant associations between one or a few SNPs and a somatic disorder and not for predicting drug effects using the composite influence of a set of SNPs associated with etiological variables. Most successful GWAS studies have far more than 1000 cases [19]–not counting controls. Some even over a million [21, 22]. The current study has 129 subjects in comparison. Moreover, human (female) sexual functioning is complex, and affected by past experiences, present psychological processes, and social interactions, and is influenced at the biological level by genetic factors, hormone-induced gene expression, and functional changes in brain neurochemistry. In other words, FSIAD is a highly complex polygenic condition [6] with multiple etiologies. Moreover, the low penetrance of individual genetic loci often means that no single SNP determines or predicts phenotype in highly complex polygenic conditions [23]. Indeed, in a study of female sexual dysfunctions, only some suggestive associations were reported [24]. Given the heterogenic nature of FSIAD we did not expect to find a single SNP, nor a small number, to have significant effect on either of the two subgroups. The effects on these subgroups are subtle, contrary to mutations such as in breast cancer type 1 (BRCA1) & breast cancer type 2 (BRCA2) that, when present, predict an approximate 70% chance of the development of breast cancer by the age of 80 [25]. If the influence of a single SNP in FSIAD would be as large as e.g. BRCA1, the subject would—arguably—have severe medical problems given the generic nature of the genes in proposed pathways—e.g. neurotransmitters.

An important precondition in our approach, contrary to GWAS and genome-wide polygenic scores [18], is the method to decrease the number of SNPs under investigation by first investigating the etiologies of the diagnosis/disease/treatment. The SNPs in those biological pathways can be preselected, decreasing the likelihood of false positive findings. This part of the present SNP filtering approach is similar to Pathways of Distinction Analysis [26] where it is applied to large GWAS datasets (e.g., breast and liver cancer) and shows strong support for using system/pathway to SNP reduction for additional analysis. Given the number of variables compared to the number of cases (297 622 SNPs and 129 subjects, respectively) there is an inherent chance of overfitting. Hence this method is focussed, primarily, on stability. The next step is to filter SNPs known to be in linkage disequilibrium and exclude SNPs with missing values. This resulted in a reduction from 297 622 to less than 300 SNPs. Note that missing values can be imputed by matching nucleotides in (near) full linkage disequilibrium if necessary [27].

FSIAD is often interpreted by neurobiological or clinically oriented scientists (and subsequently described in the media) as caused either by nature *or* nurture. Contrary to some diseases (e.g. BRCA1, sickle-cell), one may have a biological predisposition (nature) for a certain form of FSIAD, but experience (nurture) could increase (or decrease) susceptibility for impaired sexual experience as well. Thus, an exclusive neurobiological explanation for FSIAD is unlikely. Importantly, our formulae can only be applied to women with the diagnoses FSIAD. This, too, is contrary to aforementioned genetic diseases which are present when the subject has a certain genotype.

Both the T+B as the T+S results show AUCs above the clinical-use threshold of 0.80. There are, however, some shortcomings: the small sample size and the 2-week trial treatment periods. As described in Tuiten et al. [3], a substantial proportion (e.g. 20–25%) of women with 0 SSEs in a 4-week placebo run-in period had an increase of 1 SSE in a 4-week T+S or T+B treatment period. Yet the treatment period for this study were 2-weeks. Therefore, we expect another 10% to 12.5% of participants to be responders. Regarding sample size, the–combined—derivation set was 97 (75%) subjects with the validation set of 32 (25%), for both treatments. We argue the increase in the NPV, sensitivity and AUC of T+S in the validation set compared to the derivation set is a sample size artifact. We expect this artifact to disappear in larger sample sizes. An increased sample size would, further, help the described method to find more alleles or could be used to set more stringent filtering conditions.

The aim of the present study is to provide a novel procedure for the development of clinically applicable models (formulae) using SNPs to differentiate responders from non-responders on drug treatments, viable even to relatively small sample sizes.

## Supporting information

S1 Checklist (DOC)

S1 File (PDF)

S2 File (DOCX)

S1 Data (ZIP)
