# Supplementary material for: A survival of the fittest strategy for the selection of genotypes by which drug responders and non-responders can be predicted in small groups
Source: PLoS One. 2021 Mar 5;16(3):e0246828. doi: 10.1371/journal.pone.0246828 (PMC7935233; doi:10.1371/journal.pone.0246828)
Supplement: S1 File — (PDF) [file pone.0246828.s002.pdf]

CLINICAL STUDY PROTOCOL

A double-blind, randomized, placebo-controlled, cross-over study to validate the predictive power of the demarcation formula for Lybrido and Lybridos efficacy in women with female sexual interest/arousal disorder, in the domestic situation.

**Protocol Number:**	CD 001
**Version:**	Version 7
**Date of protocol:**	11 April 2014
**Sponsor:**	Companion Diagnostics BV<br>Louis Armstrongweg 78<br>1311 RL Almere<br>The Netherlands<br>Tel: 00 31 36 546 83 46<br>Fax: 00 31 36 549 71 86

SIGNATURES

Protocol Title: A double-blind, randomized, placebo-controlled, cross-over study to validate the predictive power of the demarcation formula for Lybrido and Lybridos efficacy in women with female sexual interest/arousal disorder, in the domestic situation.

Protocol Number: CD 001

Version Number: 7, dated 11 April 2014

Date of Original Protocol: version 1, 01 May 2013

REVIEWED AND APPROVED BY:

On behalf of the sponsor, Companion Diagnostics BV:

____ ____________________

Jos Bloemers, MSc, Director Scientific Operations
Emotional Brain BV. Date

____ ____________________

Kim van Rooij, MD, Principle Investigator Date

1. STUDY SYNOPSIS

Phase of Development

Phase 2

Objectives

Primary objective:

- To validate the existing demarcation formula (consisting of psychometric and biological markers) which predicts the sensitivity to Lybrido or Lybridos in women with female sexual interest/arousal disorder (FSIAD with or without FOD as secondary diagnosis), measured by the number of satisfactory sexual events (in 150 subjects).

Secondary objectives:

- To identify in additional psychometric and biological markers for the demarcation formula in order to increase predictive power for sensitivity to Lybrido or Lybridos in women with female sexual interest/arousal disorder (FSIAD with or without FOD as secondary diagnosis), measured by the number of satisfactory sexual events (in a first set of 75 subjects of the total amount of 150 subjects).

- To evaluate the altered demarcation formula in women with female sexual interest/arousal disorder (FSIAD with or without FOD as secondary diagnosis), measured by the number of satisfactory sexual events (in a second set of 75 subjects).

Study Design

This is a double-blind, randomized, cross-over, placebo-controlled study with a 2-week single-blind placebo run-in period, 3 times 2-week double-blind treatment periods, and a 1-week follow-up period. The double-blind treatment periods consists of a Lybrido (0,50 mg testosterone + 50 mg sildenafil) regime (duration 2 weeks); a Lybridos (0.50 mg testosterone + 10 mg buspirone) regime (duration 2 weeks); and a placebo regime (duration 2 weeks). Each regimen will be separated by at least a 2-day wash out period. Each subject completes the 2-week treatment period in a randomized order according to a Williams design with 6 sequences.

Subjects will visit the study site a total of 7 times: 1 screening visit, 1 start-up visit, 4 study regimen follow-up visits, and 1 final follow-up visit. During the start-up visit and study regimen follow-up visits, the subject’s sexual functioning will be evaluated, subject’s health will be monitored and study medication will be dispensed (no medication will be dispensed on the last study regimen follow-up visit, V6). Sexual satisfaction will be measured using several questionnaires, some of which will be completed at home following a sexual event. At the start-up visit, the subject will see a psycho-educational DVD with theoretical background information about sexual functioning and fantasizing exercises, to keep the basic knowledge of the subjects about sexuality on an approximately equal level. After watching the DVD the information will be discussed with a psychologist or similar healthcare provider.

The study is divided into a validation part and an exploratory part using two populations. In the validation part, the existing demarcation formula, described elsewhere, will be validated in 150 subjects with FSIAD. In the exploratory part, the possibility of an improved demarcation formula will be investigated using the first 75 of these 150 subjects and then validated in the last 75 subjects.

Number of Subjects

Approximately 150 healthy female subjects with FSIAD will be enrolled and randomized to study treatment.

Study Sites and Location

Up to 2 study sites in the Netherlands will participate to ensure that 150 subjects are randomized to study treatment.

Inclusion and Exclusion Criteria

Criteria for inclusion

Subjects must meet *all* of the following criteria:

1. Provision of written informed consent
2. Females between 18 and 70 years of age, inclusive, pre- or postmenopausal, with FSIAD (comorbidity with female orgasmic disorder [FOD]; only as secondary diagnosis) is allowed. The diagnosis of FSIAD will be established by a trained health care professional
3. Be involved in a stable, communicative, monogamous relationship and have a sexually functional partner who will be at home for a large part of the study duration
4. Healthy with normal medical history, physical examination, laboratory values, and vital signs; exceptions may be made if the investigator considers an abnormality to be clinically irrelevant
5. Use of highly effective contraception

Criteria for exclusion

Subjects who meet *any* of the following criteria are not eligible to participate in the study:

Cardiovascular Conditions

1. Any underlying cardiovascular condition, including unstable angina pectoris, that would preclude sexual activity;

2. Systolic blood pressure ≥ 140 mmHg and/or diastolic blood pressure ≥ 90 mmHg (supine blood pressure). For subjects ≥ 60 years old and without diabetes mellitus, familial hypercholesterolemia, or cardiovascular disease: systolic blood pressure ≥ 160 mmHg and/or diastolic blood pressure ≥ 90 mmHg;

3. Systolic blood pressure ≤ 90 mmHg and/or diastolic blood pressure ≤ 50 mmHg (supine blood pressure);

Gynecological and Obstetric Conditions

4. Use of any contraceptive containing anti-androgens (e.g. Cyproteron acetate) or (anti)androgenic progestogens (drospirenone, dienogest, chlormadinone acetate and norgestrel);

5. Use of any contraceptive or hormone replacement therapy (HRT) containing more than 50 µg/day of estrogen;

6. Pregnancy or intention to become pregnant during this study (Note: A urine pregnancy test will be performed in all women of child bearing potential prior to the administration of study medications);

7. Lactating or delivery in the previous 6 months prior to signing Informed Consent Form;

8. History of bilateral oophorectomy;

9. Other unexplained gynecological complaints, such as clinically relevant abnormal uterine bleeding patterns;

10. Perimenopausal status (cycle shortening/irregular menstrual bleeding in the last 12 consecutive months and/or occurrence of vasomotor symptoms (e.g. hot flashes, night sweating) and/or FSH levels (>40 IU/L) for women from age 40 onwards; in women with a history of hysterectomy perimenopausality can be assessed by FSH levels (> 40 IU/L) and/or vasomotor symptoms);

Other Medical Conditions

11. Liver and/or renal insufficiency (aspartate aminotransferase, alanine aminotransferase and gamma glutamyltransferase > 3 times the upper limit of normal and/or estimated glomerular filtration rate (eGFR) **<** 60.00 mL/min based on the Cockcroft-Gault formula)

12. Any current endocrine disease or endocrinopathy (e.g. uncontrolled thyroid function) as determined by medical history, basic physical examination and/or laboratory values significantly outside normal range of the central laboratory; or uncontrolled diabetes mellitus (HbA1c > 7.5%)

13. Free- and/or total testosterone levels outside the upper limit of the reference range of the central laboratory;

14. Any current clinically relevant neurological disease which, in the opinion of the investigator, would compromise the validity of study results or which exclude from use of sildenafil, buspirone and/or testosterone;

15. History of hormone-dependent malignancy (including all types of breast cancer);

16. Positive test result for immunodeficiency virus, hepatitis B, or hepatitis C (acute and chronic hepatitis infection);

<u>Psychological/Psychiatric Factors</u>

17. History of (childhood) sexual abuse that, in the opinion of the investigator, could result in negative psychological effects when testosterone is administered;

18. (Psychotherapeutic and/or pharmacological treatment for) a psychiatric disorder (other than those under inclusion criterion 6) that, in the opinion of the investigator, would compromise the validity of study results or which could be a contraindication for sildenafil, buspirone and/or testosterone use;

19. Current psychotherapeutic treatment for female sexual dysfunction;

20. Current genito-pelvic pain/penetration disorder according to the Diagnostic and Statistical Manual of Mental Disorders, fifth edition (DSM-5).

21. A substance abuse disorder that, in the opinion of the investigator, is likely to affect the subject's ability to complete the study or precludes the subject's participation in the study;

<u>Concomitant Medications</u>

22. Use of potent CYP3A4 inhibitors (eg, ritonavir, ketoconazole, itraconazole clarithromycin, erythromycin and saquinavir);

23. Use of potent CYP3A4 inducers (eg, carbamazepine, phenytoin, phenobarbital, St John's wort, rifampin);

24. Use of antidepressants including SSRIs, tricyclic and other;

25. Use of any other medication that interferes with study medication (eg, triptans, monoamine oxidase [MAO] inhibitors [includes classic MAO inhibitors and linezolid] and spironolactone);

26. Use of medication (including herbs) that would compromise the validity of study results;

27. Use of testosterone therapy within 6 months before study entry prior to signing the Informed Consent Form;

<u>General</u>

28. Illiteracy, unwillingness, or inability to follow study procedures;

29. Participation in other clinical trials within the last 30 days;

30. Any other clinically significant abnormality or condition which, in the opinion of the investigator, might interfere with the participant's ability to provide informed consent or comply with study instructions, compromise the validity of study results, or be a contraindication for buspirone, and/or sildenafil and/or testosterone use.

Test Product, Dose, and Mode of Administration

Lybrido is a fixed-dose combination of testosterone and sildenafil citrate. The drug product is a 9 mm, round, biconvex, white, menthol-flavored tablet for sublingual administration. The outer, polymeric, film coating contains testosterone (0.5 mg) that is released immediately after sublingual administration. The inner core of the tablet contains sildenafil (50 mg). This inner core has a polymeric coating designed to delay release of the sildenafil for approximately 2.5 hours; after this time delay, the sildenafil is released immediately (i.e., it is not a sustained release).

Lybridos is a fixed-dose combination of testosterone and buspirone hydrochloride. The drug product is a 9 mm, round, biconvex, white, menthol-flavored tablet for sublingual and oral administration. The outer, polymeric, film coating contains testosterone (0.5 mg) that is released immediately after sublingual administration. The inner core of the tablet contains buspirone hydrochloride (10 mg). This inner core has a polymeric coating designed to delay release of the buspirone for approximately 2.5 hours; after this time delay, the buspirone is released immediately (i.e., it is not a sustained release).

Placebo has the exact same appearance and flavor as Lybrido and Lybridos.

Dosing Instructions

Subjects will be instructed to keep the tablet under their tongue for 90 seconds and swish the produced saliva beneath their tongues during these 90 seconds, then to swallow the tablet as a whole thereafter, without chewing or otherwise disrupting the dosage form.
A total of 8 doses will be provided for each medication regime; 8 doses Lybrido, 8 doses Lybridos, and 2x (placebo run-in and placebo for double blind regime) 8 doses placebo. Subjects are required to take a minimum of 4 doses over each 2-week treatment regime (2 dose/week). The other 4 doses may be taken as desired (i.e., "on demand") throughout the 2-week treatment regime; dosing is permitted every day until all doses are used. If within a regime a subject uses all doses, medication will not be resupplied.

Duration of Treatment
The trial duration per subject from initial screening through the final follow-up visit is approximately 13 weeks, including a 4-week screening period, a 2-week placebo run-in period, 6 weeks on study medication, and a 1-week follow-up period. In addition, an enrolment period of 16 weeks is anticipated, so the total study duration is expected to be approximately 7 months.

Endpoints

Primary Endpoint

Change from placebo in frequency of satisfactory sexual events, following study medication intake, measured by the Sexual Event Diary (SED), item 4. This endpoint will be used for validation of the existing demarcation formula.

Exploratory Endpoints

- ◊ Change from placebo in experienced sexually-related personal distress, measured by the Female Sexual Distress Scale-Revised (FSDS-R), specifically item 13.
- ◊ Evaluation of meaningful improvement during treatment period, measured by the single item Patient's Global Impression of Improvement (PGI-I)
- ◊ Evaluation of meaningful benefit of study medication during treatment period, measured by the single item Patient Benefit Evaluation (PBE)
- ◊ Change from placebo in frequency of orgasms, following medication intake, measured by the SED
- ◊ Change from placebo in sexual desire, following medication intake, measured by the SED
- ◊ Change from placebo in physical arousal, following medication intake, measured by the SED
- ◊ Change from placebo in mental arousal, following medication intake, measured by the SED
- ◊ Change from placebo in sexual pleasure, following medication intake, measured by the SED

Safety evaluation

- ◊ Adverse events
- ◊ Physical examination (including vital signs)
- ◊ Signs of hyperandrogenism
- ◊ Laboratory assessments (including blood chemistry, hematology and hormone levels)

Measures for the demarcation formula to predict efficacy for Lybrido and Lybridos:
A variety of different genetic parameters will be assessed to calculate the current demarcation formula for each individual and to explore an improved demarcation formula for the efficacy of Lybrido and Lybridos. These parameters are based on genetic variations in the, serotonergic, androgen, noradrenergic and other pathways and the stress system, and will be combined with the measurement of hormones (e.g., serum androgen metabolites), second digit to fourth digit ratio and questionnaires regarding sexual functioning.

TABLE OF CONTENTS

1. STUDY SYNOPSIS 3
2. INTRODUCTION 18
2.1. Background 18
2.1.1. Female Sexual Dysfunction 18
2.1.2. Sex Steroids in Animals and Man: Sensitivity for Sexual Cues 19
2.1.3. Sublingual Testosterone and Treatment of Female Sexual Dysfunction 19
2.1.4. Lybrido and Lybridos 21
2.1.5 HSDD Group Subdivision 23
2.1.5.1 Biological Markers Measuring Sensitivity for Sexual Cues & Inhibitory Predisposition 23
2.1.5.2 The existing demarcation formula 26
2.1.6 Exploratory research into other potential biological markers 31
2.2 Hypotheses 32
2.2.1 Validation of existing demarcation formula 32
2.2.2 Testing of altered demarcation formula 32
2.2.2.1 Primary hypotheses explorative part 33
2.3 Benefits and Risks 33
3 INVESTIGATIONAL PLAN 36
3.2 Objectives 36
3.2.1 Primary Objective 36
3.2.2 Secondary Objectives 36
3.3 Endpoints 36
3.3.1 Primary Endpoint 36
3.3.2 Exploratory Endpoints 36
3.3.3 Safety evaluation 37
3.4 Study Design 37
3.5 Subject Selection 38
3.5.1 Number of Subjects 38
3.5.2 Inclusion Criteria 38

3.5.3 DSM-5 Criteria for FSD 38
3.5.4 Exclusion Criteria........ 40
4 STUDY ACTIVITIES AND EVALUATION METHODS 44
4.1.1 Study Medication & Route of Administration 44
4.1.2 Packaging, Labeling, and Storage of Study Medication 44
4.2 Enrollment and randomization 45
4.3 Study Procedures 45
4.3.1 Placebo Run-in Period 46
4.3.2 Visits 46
4.3.2.1 V0: Screening Visit 46
4.3.2.2 V1: Start-up Visit 48
4.3.2.3 V2, V3, V4, and V5: Study Regimen Follow-up Visits 49
4.3.2.4 V6: Final Follow-up Visit 50
4.3.2.5 Home: On-going Measurements 50
4.3.3 Description of Measurements 51
4.3.3.1 Hand Scan Measuring Digit Ratio 51
4.3.4 Questionnaires 51
4.3.4.1 Sexual Anamnesis Questionnaire-Diagnostic 51
4.3.4.2 Weekly Diary 51
4.3.4.3 Sexual Event Diary 51
4.3.4.4 Sexual Motivation Questionnaire 51
4.3.4.5 Female Sexual Distress Scale –Revised 51
4.3.4.6 Patient’s Global Impression of Improvement (PGI-I) 52
4.3.4.7 Patient Benefit Evaluation (PBE) 52
4.3.5 Program for psycho-education 52
4.3.6 Laboratory Parameters 52
4.3.6.1 Urine Drug Screening 52
4.3.6.2 Urinalysis 53
4.3.6.3 Urine Pregnancy Test 53
4.3.6.4 Hematology and Blood Chemistry 53

4.3.6.5 Luteinizing Hormone, Follicle Stimulating Hormone, Sex Steroids, Sex Hormone Binding Globulin, and Prolactin 53
4.3.6.6 Serology 53
4.3.6.7 Biological markers 53
4.3.7 Safety and Tolerability Measurements Procedure 53
4.3.8 Safety Assessments 53
4.3.8.1 Vital Signs 53
4.3.8.2 Hyperandrogenism Monitoring 54
4.3.8.3 Electrocardiogram 54
4.3.8.4 Physical Examination 54
4.3.9 Adverse Events 54
4.3.10 Food and Beverages 54
4.4 Study Termination 54
4.4.1 Regular Study Termination 54
4.4.2 Premature Study Termination 54
4.5 Breaking the Blind 55
4.6 Interim Database Lock 56
5 ADVERSE EVENTS AND SERIOUS ADVERSE EVENTS 57
5.1 Definitions 57
5.2 Evaluating AEs and SAEs 57
5.3 Reporting of SAEs and Other Significant Events 57
5.4 Other Contact Information 59
6 STATISTICAL METHODS 60
6.1 Determination of Sample Size 60
6.2 Analysis Populations 60
6.3 Study Endpoints Analyses 60
7 STUDY ADMINISTRATION 63
7.1 Quality Assurance 63
7.2 Monitoring 63
7.3 Archiving and Data Handling 63
7.4 Drug Accountability 63

7.5 Ethical Considerations 64
7.5.1 General Regulations 64
7.5.2 Informed Consent 64
8 DATA MANAGEMENT 65
8.1 General 65
8.2 e-Case Report Form 65
8.3 Data Transfer 65
8.3.1 Data for the Viedoc Clinical Database 65
8.3.2 Transfer of Data 65
9 REPORTING, PUBLICATION, AND ARCHIVING 66
9.1 Reporting 66
9.2 Publication(s) 66
9.3 Archiving of Data 66
10 INVESTIGATOR AGREEMENT 67
11 REFERENCES 68
12 APPENDICES 76

List of In-text Tables

Table 1: Overview of Study Visits 46
Table 2: Measurements and Activities at V0 47
Table 3: Measurements and Activities >12 weeks after screening 47
Table 4: Measurements and Activities at V1 48
Table 5: Measurements and Activities at V2, V3, V4, and V5 49
Table 6: Measurements and Activities at V6 50

LIST OF ABBREVIATIONS

Abbreviation	Definition
2D:4D	second digit to fourth digit ratio
3α-diol-G	5 alpha-Androstane – 3 alpha- 17 beta-diol glucuronide
5-HT	Serotonin
5-HT1a	serotonin 1 alpha
5-HTTLPR	serotonin transporter-linked polymorphic region
ADT-G	androsterone glucuronide
AE	adverse event
ANOVA	analysis of variance
AR	androgen receptor
AVP	arginine vasopressine
BMI	body mass index
BOLD	blood oxygenation level dependent
bpm	beats per minute
CAG	trinucleotide (codon): cytosine, adenine and guanine
CAIS	complete androgen insensitivity syndrome
cGMP	cyclic guanosine monophosphate
CRA	clinical research associate
CRF	case report form
CRH	corticotropin-releasing hormone
DHEA	dehydroepiandrosterone
DHT	dihydrotestosterone
DSM	diagnostic and statistical manual of mental disorders
DSM-IV-TR	DSM, fourth edition, text revision
DSM-5	DSM, fifth edition
DVD	digital versatile disk
ECG	electrocardiogram
eGFR	estimated Glomerular Filtration Rate
FOD	female orgasmic disorder
FSAD	female sexual arousal disorder

Abbreviation	Definition
FSDS-R	Female Sexual Distress Scale –Revised
FSIAD	Female Sexual Interest / Arousal Disorder
GC	glucocorticoid hormone
HIV	human immunodeficiency virus
HSDD	hypoactive sexual desire disorder
5-HTR1a	serotonin receptor 1a
MAO	monoamine oxidase
MC	Melanocortin
NANC	Non-adrenergic/Non-cholinergic receptors
NET	Norepinephrine transporter
NO	nitric oxide
PDE-5	phosphodiesterase type 5
POMC	pro-opiomelanocortin
SAE	serious adverse event
SAQ-D	Sexual Anamnesis Questionnaire-Diagnostic
SERT	serotonin transporter
SHBG	sex hormone binding globulin
SMQ	Sexual Motivation Questionnaire
SED	Sexual Event Diary
STAR	Steroidogenic acute regulatory protein
SUSAR	suspected unexpected serious adverse reaction
VPA	vaginal pulse amplitude
VTA	ventral tegmental area

DEFINITION OF TERMS

Existing demarcation formula: Sensitivity for sexual cues and maladaptive activity of sexual inhibitory mechanism(s): The brain's sensitivity for sexual cues and its predisposition for (sexual) inhibitory activity is influenced by the function of the androgen and serotonergic systems and reflected behaviorally as motivational stances towards emotionally relevant cues. Sensitivity to sexual cues and dysfunctional activity of sexual inhibitory mechanism(s) is therefore determined using the following measures:

1. Trinucleotide cytosine-adenine-guanine repeat length of the androgen receptor gene polymorphism (CAG repeat)
2. Serotonin (5-HT) transporter and 5-HT_{1a} receptor genotype polymorphism
3. Androgen metabolites: androsterone glucuronide (ADT-G) and 3-alpha-androstanediol glucuronide (3AG)
4. Second digit to fourth digit ratio (bilateral hand scan)
5. Sexual motivation and inhibition (using the Sexual Motivation Questionnaire [SMQ])
6. Sexual arousal and desire (using the Sexual Anamnesis Questionnaire [SAQ-D])

New biomarkers that will be measured to possibly improve the demarcation formula (using microarray chips) are:

1. Dopamine receptor genotype polymorphism (D1-D5 receptor) and the dopamine transporter
2. All serotonin receptors and the serotonin transporter
3. Corticotropin-releasing hormone (CRH)/ arginine vasopressine (AVP)/ glucocorticoid hormone (GC)/ Melanocortin (MC)/ pro-opiomelanocortin (POMC) receptor genes
4. Noradrenergic transporter (NET), Non-adrenergic/Non-cholinergic receptors (NANC)
5. Oxytocin receptors
6. Prolactin gene polymorphism
7. Steroidogenic acute regulatory protein (STAR), Cytochrome P450 enzymes, Dehydroepiandrosterone (DHEA), Dihydrotestosterone (DHT),Sex-hormone binding globulin (SHBG) gene polymorphism, SHBG receptor polymorphism, aromatase/5-alpha-reductase
8. Nitric Oxide (NO) synthase, Phoshodiesterase type 5 (PDE5) enzymes

2. INTRODUCTION

2.1. Background

2.1.1. Female Sexual Dysfunction

Low sexual desire, with or without sexual arousal problems, is the most common sex-related complaint reported by women. As a result, many women suffer from sexual dissatisfaction, which often negatively interferes with psychological and social wellbeing. This has been classified as a clinical condition, referred to as Hypoactive Sexual Desire Disorder (HSDD [1]) (American Psychiatric Association, 2000). A range of biological and psychological regulatory mechanisms control human sexual functions. However, the degree of activation of these mechanisms (i.e. their relative strength) varies within and between subjects. One possible reason for the limited success in developing drug treatments for HSDD could be the lack of consideration of multiple interacting biological *and* psychological mechanisms in the existence of individual differences in sexual (dys)function. In humans, a range of complex cognitive and affective processes mediate the transition from a preparatory sexual motivational state to actual sexual behavior. These conscious and subconscious processes are partly the result of present and previous beneficial and/or adverse experiences and significantly influence the extent to which an individual is willing to engage in sexual activities and the extent to which those activities are enjoyable. Therefore, the delicate and relative balance between activation of central mechanisms governing sexual excitation and sexual inhibition, controls sexual activities in humans, including fantasizing, masturbation and sexual intercourse.

For our drug development program we assumed that different causal mechanisms might be responsible for seemingly similar symptoms associated with HSDD. In particular, in some women with HSDD, sexual dysfunction results from a relative insensitivity for sexual cues, whereas in others, sexual complaints result from dysfunctional automatic activation of sexual inhibitory mechanisms during sexual stimulation. The suggested causal mechanisms are related to different levels of the brain's sensitivity to sexual stimuli. The degree of this sensitivity for sexual cues or stimuli is largely influenced by biological factors, such as hormonal levels and genetic factors.

These differentiations were taken into account when designing and developing two new medications for HSDD: *Lybrido* has been developed for a subgroup of patients in which low sexual motivation and desire occurs as the result of a relatively insensitive system for sexual cues; *Lybridos* has been developed for a subgroup of patients in which the sexual complaints result from over-activation of sexual inhibitory mechanisms.

[1] Hypoactive Sexual Desire Disorder is a diagnosis from the Diagnostic and Statistical Manual of Mental Disorders fourth edition (text revision (DSM-IV-TR)). In May 2013 the DSM-5 was published. The Hypoactive Sexual Desire Disorder (HSDD) and the Female Sexual Arousal Disorder (FSAD) are combined and replaced by the Female Sexual Interest/Arousal Disorder (FSIAD).Because previous studies were all executed with HSDD diagnosed women, we will refer to this indication instead of FSIAD throughout this background paragraph).

Lybrido is sublingual testosterone combined with a PDE-5 inhibitor, in which the time of the peak plasma concentration of the PDE-5 inhibitor coincides with the 4 hour delay in behavioral effects of the testosterone. *Lybridos* is sublingual testosterone combined with a 5-HT_{1A} receptor agonist, in such a timeframe that the pharmacological effects of the 5-HT_{1A} receptor agonist coincide with the behavioral window induced by the testosterone administration.

In the next paragraphs we describe the theoretical background of the development of Lybrido and Lybridos.

2.1.2. Sex Steroids in Animals and Man: Sensitivity for Sexual Cues

Animal models of sexual behavior have focused on the relationship between brain mechanisms and behavioral indices of sexual function. In many mammalian species, female sex steroids are essential for the expression of female sexual behavior. Animal research has revealed a steroid-responsive neural network (Cottingham and Pfaff, 1986), which is involved in selective filtering of sensory input and amplification of signals that may facilitate sexual behavior. The ability of cues to evoke sexual responses is related to individual differences, such as the sensitivity of the respective steroid receptor systems, sex steroid levels, and environmental factors, such as the proximity of an attractive mate. In higher primates, like humans, sexual intercourse is not limited to the peri-ovulatory period, and it has been suggested that testosterone (T) plays an important role in socio-sexual behavior of women (Freeman and Rissman, 1996). This is supported by the fact that the depletion of T in women following ovariectomy and adrenalectomy, is accompanied by a complete loss of libido(Waxenberg et al., 1959), while substitution of T restores sexual desire and fantasies after surgical menopause (Sherwin et al., 1985). Because testosterone appears to play a central role in the steroid-responsive neural network of human (males and) females, it will influence the sensitivity of the brain for sexual cues in both sexes.

2.1.3. Sublingual Testosterone and Treatment of Female Sexual Dysfunction

Single administration of sublingual testosterone (0.5 mg) can induce an increase in physiological and subjective indices of sexual responding in sexually functional women (Tuiten et al., 2000;Tuiten et al., 2002). This might indicate this dosage form as a potential pharmacotherapy for hypoactive sexual desire disorder (HSDD).

In a randomized, placebo-controlled, double blind, cross-over study, we investigated the efficacy of sublingual testosterone (0.5 mg), the PDE5 inhibitor vardenafil and the combination of these drugs on pre-conscious attentional bias for sexual cues and physiological sexual function in women diagnosed as having HSDD. We assume that the delay in effect of testosterone on physiological sexual arousal in sexually functional women occurred as the result of an increase in activation of central sexual motivational mechanisms. Central sexual stimulation is necessary for a PDE5 inhibitor to induce an increase in the amount of blood in erectile tissue of the genitals. This occurs in the following way: In genital erectile tissue of both men and women, sexual stimulation will induce the release of nitric oxide (NO) from nerves and endothelium. NO induces an increase in

production of cyclic guanosine monophosphate (cGMP). cGMP is a key mechanism in relaxing smooth muscle necessary for the induction of enlargement of the erectile tissue. cGMP is hydrolyzed by the phosphodiesterases in the corpora cavernosa, in which phosphodiesterase type-5 is the most abundant PDE. Therefore, during sexual stimulation, the action of NO/cGMP on erectile function will be enhanced by PDE5 inhibitors (Goldstein et al., 1998). Thus, without adequate central stimulation, i.e. activation of central sexual motivational mechanisms, a PDE5 inhibitor cannot be effective, which is likely why the trials investigating PDE5 inhibitor efficacy in FSD failed.

Indeed, in former research it has been shown that PDE-5 inhibitors alone have no effect on physiological sexual functioning of patients, but neither did testosterone alone. In contrast, the combined use of sublingual testosterone and the PDE5 inhibitor produced an increase in physiological sexual responding about four hours after the intake of the testosterone (Van der Made et al., 2009). Apparently, in addition to testosterone-mediated facilitation of central sexual stimulation, peripheral facilitation of the physiological sexual response occurred and was needed. Interestingly, we found a striking difference in effect between women who had and women who had not reported the experience of childhood sexual abuse (CSA). In women without CSA, testosterone treatment induced an increase in their originally low levels of preconscious attentional bias for sexual cues, while women with CSA showed a decrease in their originally high levels of attention. The effects of the combination of testosterone and the PDE-5 inhibitor on the physiological sexual response also differed between these groups. Women without CSA revealed an increase in their physiological response, while the women with CSA showed no alterations in this response.

These results were reproduced in a second randomized, double blind, cross-over, placebo-controlled study in 28 women with HSDD (van der Made et al., 2009). This study was designed to investigate the effects of testosterone, a PDE5-inhibitor, and the combination of both drugs on alterations in preconscious attentional bias for sexual cues. Moreover, we investigated the influence of these drugs on physiological and subjective indices of sexual function during neutral and erotic visual stimulation. In this study women who reported experience of CSA were excluded. Because other (negative) sexual experiences can influence preconscious attentional bias for erotic cues, we examined possible group differences in preconscious attentional bias for erotic cues and its effects on physiological and subjective sexual responding. Again, neither testosterone (0.5 mg) nor the PDE5 inhibitor (vardenafil, 10mg) alone had an effect on measures of physiological and subjective arousal. In two subgroups, which were differentiated on the basis of their initial preconscious attentional bias for sexual cues, a different sexual response profile was found. Testosterone treatment, in the group initially low in attention for sexual cues, produced an increase in their attentional bias for sexual cues, while in the group that initially had a high attention a reversed pattern was found. The combined administration of testosterone and vardenafil in the former group produced a significant increase 4 hours post dose in physiological and subjective sexual functioning, while in the latter group no drug induced alterations in these measures were observed. This study had no subjects with a history of CSA, but the latter group did have a higher prevalence of negative sexual experiences (63% vs. 17%), which implies that this group’s past experience (as in the CSA

group of the former study) may have contributed to the development of HSDD as a result of activation of central inhibitory mechanisms.

2.1.4. Lybrido and Lybridos

These findings led to the hypothesis that there are (at least) 2 subgroups with different biological causes for the sexual complaints in HSDD. Women in the group with low initial preconscious attention for erotic cues (or none at all) suffer from reduced central sensitivity for erotic cues. Women in the second group, with normal to high initial preconscious attention for erotic cues, suffer from dysfunctional activation of sexual inhibitory mechanism(s).

Bancroft and Janssen (Janssen and Bancroft, 2007) conceptualized that individual differences in sexual responding depend on a delicate interplay of excitatory and inhibitory processes. It is widely accepted that the prefrontal cortex (PFC) is involved in the inhibitory control of human behavior (Chambers et al., 2009), including sexual behavior (Beauregard et al., 2001;Stoleru et al., 2003). A sexual event which is consciously or subconsciously negatively valenced or which induces inappropriate responses in, for that response, inappropriate situation, can be expected to induce a phasic increase in PFC mediated sexual inhibition. Amplifying negative valence or response sensitivity in an inappropriate setting would be expected to increase inhibition even further. Indeed, in two studies (unpublished data) we found support for this hypothesis, in that testosterone can actually induce an inhibitory sexual response.

In the first experiment we tentatively examined whether treatment with testosterone would cause an increase in neural activity in brain areas associated with sexual behavior. In a randomized, placebo-controlled, double-blind, crossover design, a functional Magnetic Resonance Imaging (MRI)-Vaginal Pulse Amplitude (VPA) study was conducted in 12 sexually functional eugonadal female volunteers. Four hours after the intake of the placebo or 0.5 mg of testosterone sublingually, the blood oxygen level dependent (BOLD) MRI response was measured while subjects viewed neutral and erotic film excerpts.

As expected, we found comparable BOLD responses to erotic stimuli in the placebo condition, as in other imaging studies (Karama et al., 2002;Stoleru et al., 1999): the amygdala and temporal pole, hippocampus, hypothalamus, brainstem, orbitofrontal cortex (OFC), anterior cingulate cortex (ACC) and decreased BOLD response of dorsal prefrontal areas (Figure 1, top row). We expected this pattern to become more pronounced following testosterone administration relative to placebo.

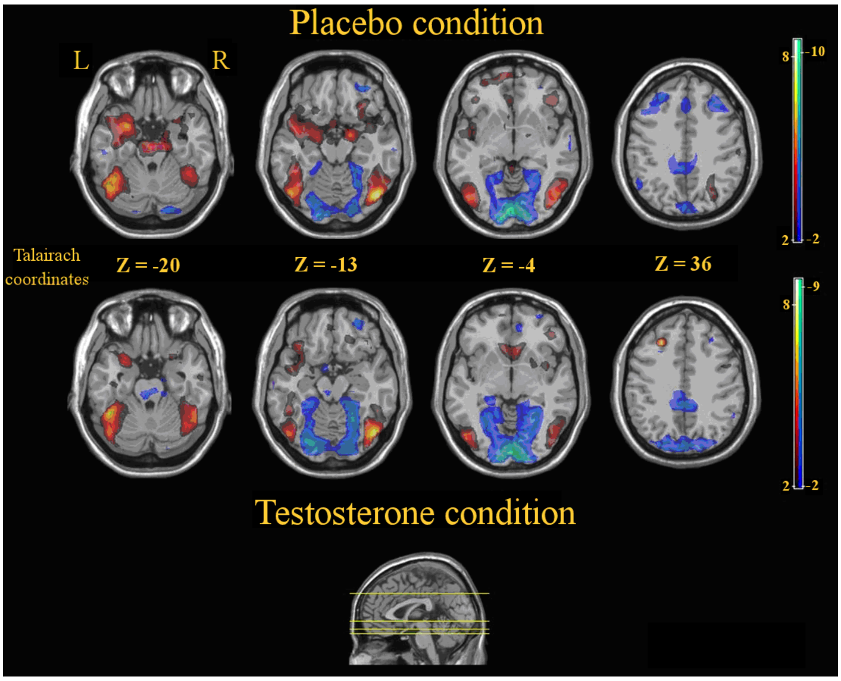

Figure 1. BOLD Responses to erotic stimuli under placebo and testosterone. Transverse images of the brain. Z-coordinates in Talairach space **(**Source: Unpublished results).

In the testosterone condition, however, women showed decreased BOLD responses in all brain structures implicated in the normal sexual response (Figure 1, bottom row). Additionally, there was an increased BOLD response in the superior part of the middle frontal gyrus, a portion of the dorsolateral prefrontal cortex (DLPFC), which mediates inhibition of task-irrelevant responses (Konishi et al., 2003;Liddle et al., 2001;Liu et al., 2004;Milham et al., 2003;Rubia et al., 2001) and willful inhibition of the sexual response(Beauregard et al., 2001). Also, there was increased BOLD response of the septal nuclei, which have been postulated to play a constrictive role on subcortical structures, reducing emotional and arousal extremes, thus preventing emotional overshoot(Joseph, 1999).

Further neurophysiological evidence for a possible HSDD subdivision was derived from Study EB64 (see the Investigator’s Brochure). In this study functional MRI was used to measure functional BOLD activity in response to erotic stimuli under different levels of attentional engagement, 2 subgroups emerged, according to their propensity to inhibit their functional ‘sexual’ brain activity in response to Lybrido.

These findings prompted the development of *Lybridos*, an on-demand drug combination of sublingual testosterone plus a 5-HT_{1A} receptor agonist (buspirone hydrochloride), for HSDD when the symptoms are the result of dysfunctional activation of inhibitory sexual mechanisms. An important biochemical mediator of inhibitory mechanisms is the neurotransmitter 5-hydroxytryptamine (5-HT, serotonin), which exerts part of its inhibitory effect in the PFC. Acute treatments with 5-HT_{1A} receptor agonists decrease serotonergic activity and the release of serotonin. Consequently, acute treatment with a 5-HT_{1A} receptor agonist may result in decreased

serotonergic activity in the PFC, which in turn might prevent or reduce the inhibitory response to sexual cues in women with this type of HSDD. Accordingly, when exposed to sexual stimuli (whether internally or externally induced), women prone to sexual inhibition might show an increased physiological and subjective sexual response when treated with a combination of testosterone and a 5-HT_{1A} receptor agonist (i.e., Lybridos), in such a timeframe that the pharmacological effects of the 5-HT_{1A} receptor agonist coincide with the behavioral window induced by the testosterone administration.

In Study EB70 (see the Investigator's Brochure and (Poels et al., 2013;van Rooij et al., 2013)) it was shown that the subdivision of HSDD in 2 groups, 1 with a relatively insensitive system for sexual cues and 1 with increased activity of sexual inhibitory mechanisms, accurately predicted the efficacy of Lybrido and Lybridos. The effects of Lybrido and Lybridos on physiological and subjective sexual responses were evaluated in an ambulatory psychophysiology laboratory at home for 1 week. Subsequently, sexual improvement and satisfaction during use of the 2 drugs were measured (in the bedroom) for 3 weeks. Lybrido and Lybridos significantly improved sexual functioning in both studies. The level of improvement attained depended on the underlying etiology: Lybrido worked best in women with low sensitivity to sexual cues, whereas Lybridos was most effective in those with high levels of sexual inhibition. In addition, women with a high sensitive sexual system as compared with low sensitive women might be more sensitive for positive sexual experiences, which can lead to a positive sexually lusty life. However, a high sensitive system for sex means also more sensitivity for negative sexual experiences, which can result in an increased proneness for (learning of) activation of automatic inhibitory mechanisms during sexual stimulation. Thus we assume that women with HSDD as the result of activation of sexually inhibitory mechanism have also a more sensitive system for sexual stimuli.

2.1.5 HSDD Group Subdivision

The subdivision of HSDD subjects has to date been based on their preconscious attentional bias, as measured using an adapted Stroop task (discussed below). However, in Study EB85 (see the Investigator's Brochure), a psychobiological basis for the HSDD group subdivision was explored. Here we will discuss some of the variables involved in this psychobiological basis for high and low sensitivity for sexual cues.

2.1.5.1 Biological Markers Measuring Sensitivity for Sexual Cues & Inhibitory Predisposition

Androgenic Sensitivity

In premenopausal women, testosterone is produced by the ovaries (25%), the adrenal glands (25%), and the peripheral conversion (50%) of the prehormones androstenedione and its precursor dehydroepiandrosterone (DHEA). Androstenedione is produced by the ovaries (50%) and adrenals (50%), while DHEA and DHEA sulfate, are almost exclusively produced by the adrenals (90% to 95%).

Menopause and age-related atrophy of the adrenal cortex influence this DHEA/DHEA sulfate ratio (Brand and van der Schouw, 2010;Longcope, 1986).

Since in females the peripheral conversion of the various prehormones is so important, not only serum testosterone but in particular peripheral conversion of its precursors determine the physiological effects of androgens in women. Of note, testosterone and its precursor androstenedione are in turn aromatizable and can be converted into estradiol and estrone, respectively. The production and action of sex steroids in peripheral target cells is called intracrinology (Labrie et al., 2005). Because a large proportion of sex steroids is synthesized and metabolized in peripheral tissues, serum testosterone alone cannot be used to gauge androgen tissue exposure or action.

In women, the levels of the 2 main metabolites of androgens, androsterone glucuronide (ADT-G) and 3-alpha-androstanediol glucuronide (3α-diol-G), are 70% of those found in men of the same age, while the level of serum testosterone is 3% of that in men. This suggests that serum testosterone alone is not a valid marker of androgenicity in women, and that locally produced glucuronide derivatives of androgens are better markers of androgenic activity (Labrie et al., 2005). Therefore, serum concentrations of ADT-G, and 3α-diol-G can be determined as a measure of overall androgenic activity.

Various testosterone concentrations within the normal range are able to saturate androgen receptors. Thus, it can be argued that within the range of normal hypothalamic-pituitary-gonadal-axis function and eugonadal plasma testosterone concentration, genetically determined functional differences in androgen receptor activity in target tissues can be observed and will be of clinical significance.

The influence of testosterone on the sensitivity of the brain may be mediated by different, but interrelated, variables. The first intervening variable is associated with the androgen receptor. This receptor belongs to a superfamily of nuclear receptors which are present in the cytoplasm and act as transcription factors to regulate gene expression. After binding of testosterone/dihydrotestosterone (DHT), a conformational change occurs, leading to dissociation of the receptor from 'heat-shock' proteins. This activated ligand-bound receptor then translocates to the nucleus, binds to DNA, and initiates transcription through binding to response elements of the target gene. The variation in the effects of testosterone in the normal range on sexual function will be mediated by mechanisms involving the androgen receptor. Mutations of the androgen receptor gene cause a variety of androgen insensitivity syndromes. In the normal population, the trinucleotide cytosine adenine guanine (CAG) repeats expansion (coding for polyglutamine tract) of the first exon of the androgen receptor polymorph, with the length varying from 11 to 38 repeats; the longer the CAG repeats, the weaker the receptor transactivation (Quigley et al., 1995;Zitzmann, 2009). Women with a relatively long CAG repeat length are assumed to have a relatively less functioning androgen system, and consequently, low sensitivity for sex.

The second intervening variable is related to the differentiation between organizational and activational effects of testosterone on the brain. Testosterone preprograms the brain during early development, and later in life, it selectively modifies brain processing to facilitate or inhibit behaviors depending on the social context. In humans, the influence of testosterone on brain

organization has to be considered critical during the fetal period of prenatal development (between weeks 12 and 19 of gestation). The activational effects of this steroid become prominent and manifest during adolescence and adulthood (Breedlove et al., 1999;Knickmeyer et al., 2005;Manson, 2008;Manson, 2008;Okten et al., 2002). The second digit to fourth digit ratio (2D:4D) may be a valuable marker for prenatal testosterone; women generally have higher digit ratios than men (Manning et al., 1998). Furthermore, studies in subjects with complete androgen insensitivity syndrome (CAIS) provide a good example of prenatal testosterone effects. Women with CAIS had high (i.e., extremely feminized) digit ratios;(Berenbaum et al., 2009) in contrast, women with congenital adrenal hyperplasia had significantly lower (i.e., more masculine) digit ratios, reflecting high prenatal testosterone levels (Brown et al., 2002;Ciumas et al., 2009;Okten et al., 2002). Women with relatively high digit ratios are assumed to have a relatively low sensitivity system for sex.

Serotonergic Sensitivity: Serotonin Transporter and 5 HT_{1A} Receptor

Serotonergic transmission affects a wide range of behaviors, from food intake and reproductive activity, to sensory processing and motor activity, to cognition and emotion. One key regulator is the serotonin transporter (SERT), which removes serotonin (5-HT) from the synaptic cleft. The SERT protein is encoded by a single gene, SLC6A4. Transcriptional activity of human SLC6A4 is modulated by several variations, including the SLC6A4-linked polymorphic region (5-HTTLPR), which is composed of a short and long version that result in differential SERT expression and function (Canli and Lesch, 2007). Short variant carriers are associated with less SERT expression and function relative to long variant carriers. Consequently, extracellular 5-HT concentrations are increased and 5-HT receptor functions are dysregulated within short variant carriers (Murphy et al., 2008). Several studies have demonstrated that short variant carriers are associated with greater amygdala reactivity in response to emotionally provocative stimuli compared to neutral stimuli, showing attention bias patterns to positive and negative emotion faces (Beevers et al., 2010;Perez-Edgar et al., 2010). Relative loss of SERT gene function increases anxiety and has a negative influence on the capacity to cope with stress. Furthermore, individuals with the short repeat of the 5-HTTLPR are at greater risk for depression following stress (Canli and Lesch, 2007;Hariri and Holmes, 2006).

Another key regulator in the serotonin system is the 5-HT_{1A} receptor, encoded by the 5-HT receptor 1A (HTR1A) gene. A common C(-1019)G polymorphism in the 5-HT1a receptor promoter region is associated with altered expression of the 5-HT_{1A} receptor. The G allele or G/G genotype, as compared to the C allele or C/C genotype, has been shown to be a risk factor for anxiety and depression, like the short variant of the 5-HTTLPR gene (Le Francois et al., 2008;Mekli et al., 2010;Zhang et al., 2009). The combination of risk alleles of the 5-HTTLPR and 5-HT1a receptor genes have been associated with reduced response to antidepressants (Arias et al., 2005).

Studies in rats have shown that the serotonin system plays an important role in sexual behavior. Considering the effects on the serotonergic transmission induced by polymorphism of the 5-HTTLPR gene and 5-HT_{1A} receptor gene, these genetic parameters could be significant in the study of sexual behavior and the development of targeted treatment modalities for women with HSDD/FSAD.

2.1.5.2 The existing demarcation formula

In an iterative search process using the data gathered from Studies EB85 and EB70 (see above) a diagnostic working model has been formulated. This model uses an individual's CAG repeat length of the androgen receptor, their $5HT_{1A}$ receptor genotype, their ADT-G and 3α-diol-G levels, their 2D:4D ratio, and 5 questionnaire items (from the Sexual Motivation Questionnaire [SMQ] and the Sexual Anamnesis Questionnaire–Diagnostic [SAQ-D]) to investigate their responsiveness to Lybrido and Lybridos.

In our search for a possible relationship between, on the one hand, the above mentioned biological factors, and, on the other hand, the sensitivity for sexual cues (low vs. high) and the responsiveness to Lybrido and Lybridos, we used in our calculations the z-scores of these variables based on 201 study subjects of the EB85 study. Furthermore, we have made use of 41 patients from which we had access to the biological markers (EB85), but also to the results of Lybrido and Lybridos on their sexual satisfaction (EB70) during sexual events.

The aim of our endeavor was to find one demarcation-formula, which could make an optimal differentiation between the effects of Lybrido and Lybridos in our patients. Although we were aware that we would lose information, we took as dependent variable the difference in effect between Lybrido and Lybridos on sexual satisfaction.

In our iterative search-process we used the following (relatively simple) assumptions:
Firstly, for the supposed influence of the different biological markers on sensitivity to sexual cues, we supposed the following directions:

Biological marker	Low Sensitive Women	High Sensitive Women
CAG repeat Androgen receptor	Long repeat length	Short repeat length
2D4D ratio	High ratio	Low ratio
3^{α}-diol-G & ADT-G	Low levels	Normal/High levels
Serotonin $_{1A}$ receptor genotype	C variant (CC)	G variant (GG or CG)

Secondly, we assumed that these biological markers can strengthen and weaken each other in their effects on the dependent variable. As an illustrative example, we looked at the cumulative effects of the androgen variables and serotonin variable (sums of z-scores) on the relative increase on sexual satisfaction (or decrease) of both drugs compared to placebo [(drug - placebo) / placebo] * 100.

**Relative increase in sexual satisfaction (n=41)**	**HIGH SENSITIVITY:**<br>**$5HT_{1A}$ G-carriers**	**LOW SENSITIVITY:**<br>**$5HT_{1A}$ CC**
**HIGH SENSITIVITY:**<br>Low 2D4D ratio, short CAG and high androgen metabolites	**Lybrido 10,4% increase**<br>**Lybridos 30,6% increase**	Lybrido 9,1% increase<br>Lybridos 14,6% increase
**LOW SENSITIVITY:**<br>High 2D4D ratio, long CAG and low androgen metabolites	Lybrido 4,7% increase<br>Lybridos 2,5% increase	**Lybrido 35,6% increase**<br>**Lybridos -11,6% decrease**

Based on these findings we looked at the relationship between the total of z-scores of the biological markers (= biological part of the demarcation-formula) and the dependent variable (e.g. difference on sexual satisfaction between Lybrido and Lybridos). Subsequently we made a comparison between the originally used Stroop task (which measures preconscious attentional bias for sexual cues, an indirect measure for sexual cue sensitivity) and the biological part of the demarcation formula on the dependent variable. The most obvious difference between both measures of sensitivity is the increase in explained variance of the dependent variable by the biological part of the demarcation-formula.

Distribution in the low or high sensitivity grouping variable for both independent variables (Stroop task and biological part of the demarcation formula) is determined by the direction of the resulted sign (negative or positive). The following figures show the relationship between the measurements of sensitivity for sex according to the Stroop task and the biological part of the demarcation-formula respectively, with the dependent variable:

Relationship between sensitivity of the brain and sexual satisfaction, according to the Stroop task

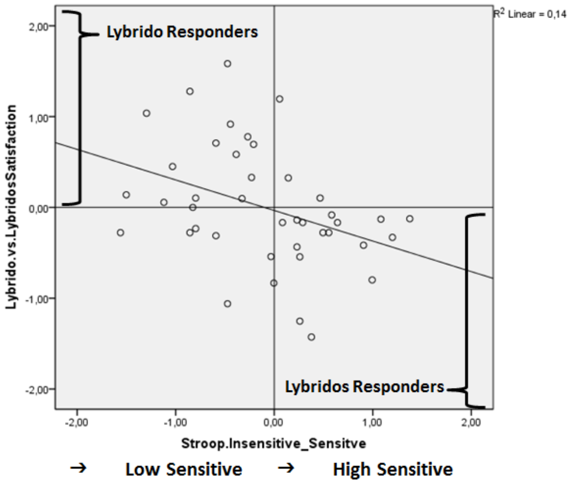

Relationship between sensitivity of the brain and sexual satisfaction, according to the Biological System

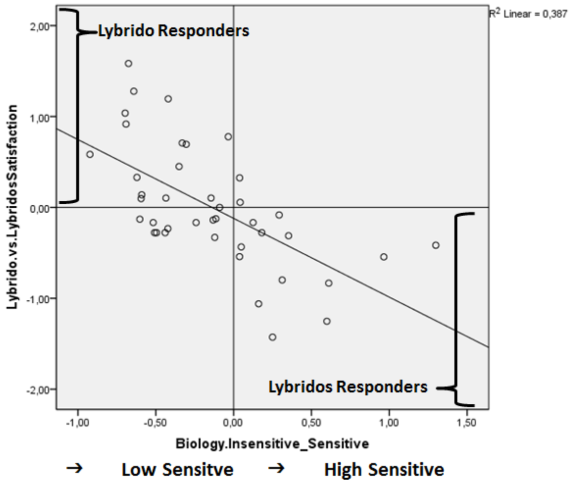

Based on these results we looked at additional subjective variables in order to further increase the explained variance of the dependent variable. The resulted combination of the biological and subjective systems leads to the following relationship between the independent and dependent variable:

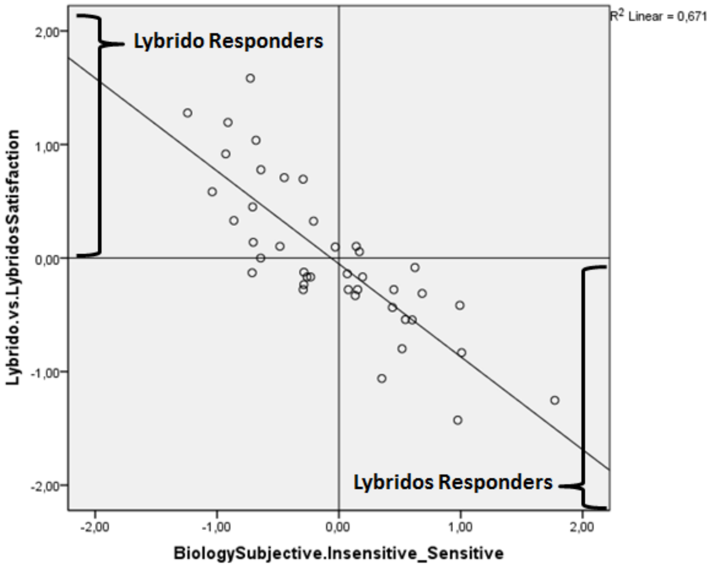

→ **Low Sensitve** → **High Sensitive**

This demarcation formula has been used as an inclusion criterion in two Phase 2b studies in the US (EB82 and EB90; see Investigator Brochure). For each drug condition (Lybrido and Lybridos) 210 women are included according to the following decision tree:

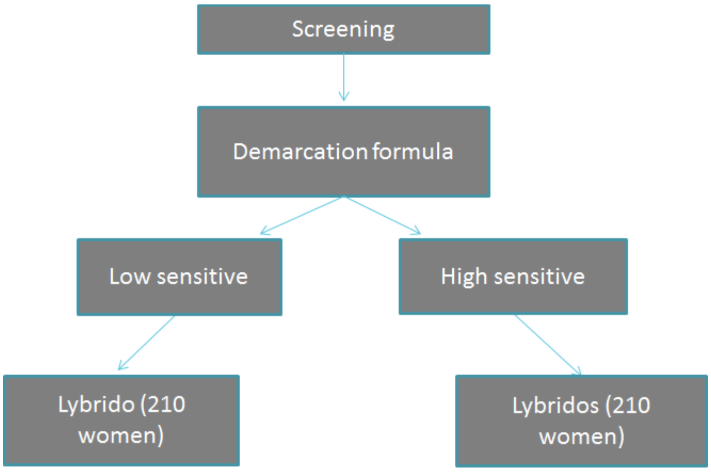

In both studies the following design was used (note that for the Lybridos study, the sildenafil 50 mg arm was replaced by a buspirone 10 mg arm, and the 4 Lybrido dose arms by 4 Lybridos dose arms):

	Baseline	Placebo run in	Placebo run in	Treatment	Treatment
n = 30	Baseline	Placebo run in		Placebo	
n = 30	Baseline	Placebo run in		Sildenafil 50 mg	
n = 30	Baseline	Placebo run in		T 0.50 mg	
n = 30	Baseline	Placebo run in		Lybrido dose 1	
n = 30	Baseline	Placebo run in		Lybrido dose 2	
n = 30	Baseline	Placebo run in		Lybrido dose 3	
n = 30	Baseline	Placebo run in		Lybrido dose 4	
	4 weeks	8 weeks		8 weeks	

Changes in the number of satisfactory sexual events between the baseline and the different drug conditions (treatment arms) in our Phase 2b study (EB82) for Lybrido are shown below (the Phase 2B study (EB90) for Lybridos has not been unblinded yet):

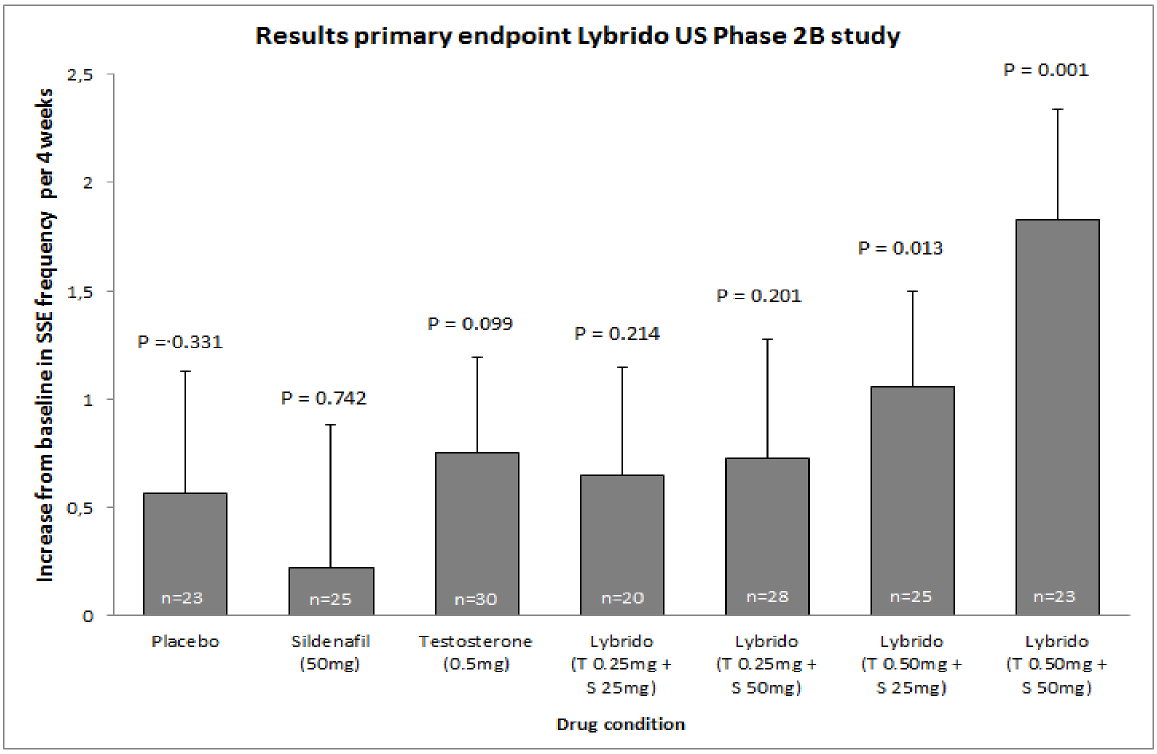

These results demonstrate that based on the selection by the use of the demarcation formula, the highest dosage of Lybrido produced the highest and significant effect in the primary endpoint. Further analyses revealed statistically significant interaction effects for the comparison between Placebo and the highest dosage of Lybrido.

Although these results demonstrate that the demarcation formula is adequate for its purpose (e.g. assigning Lybrido treatment to a subdivision of women with HSDD), it may become more sensitive if

additional biological markers are included. Moreover, if the 2D:4D ratio and questionnaire items can be replaced by other biological markers, such a demarcation formula would be easier to use for a clinician. The demarcation formula will be validated for use in several phase 3 studies, and ultimately be used in the clinical setting should Lybrido and Lybridos receive marketing approval.

2.1.6 Exploratory research into other potential biological markers

The above results demonstrate that genetic variation may impact a subject's response to therapy. The other way around, variable response to therapy may be due to genetic determinants that impact the disease etiology and /or the metabolism and mechanism of action of the drug. The primary objective of the present study is validation of the current demarcation formula.

A secondary objective is to assess in an exploratory way the involvement of additional genetic and biological parameters influencing sensitivity of the brain. We want to investigate in an iterative search process whether adding and/or substitution by other genetic and biological variables (involved in sexual behavior) the demarcation formula can be improved, in the sense that it becomes more practical and/or works as a better predictor for the differentiation of Lybrido and Lybridos responders.

For instance, polymorphisms in the Androgen, SHBG and Aromatase promoter region have been identified to be highly variable. Long repeats in the SHBG promoter region in combination with short microsatellite allele of Aromatase, or short CAG repeat of the Androgen receptor are reported to act synergistic on hyper-androgenic phenotype (Coviello et al., 2012;Vanbillemont et al., 2009;Xita et al., 2008;Xita and Tsatsoulis, 2010). Individuals with genetically determined low SHBG levels may be exposed to higher free testosterone throughout life but more importantly during fetal life when programming of the different target tissues takes place.

The central dopaminergic and serotonergic systems are key elements of the control of sexual function both in the pre-copulatory or appetitve, and consumatory phase. Polymorphisms of both systems have been implicated in the regulation of cognitive, motivational and reward processes, and have been associated with different behavioral syndromes, e.g., reward deficiency syndrome and impulsive, addictive behavior (Varga et al., 2012).

It is conceivable that these genetic variations in association with other (yet) unknown polymorphisms have impact on the sensitivity of the brain for sexual cues and as a result the sexual responsiveness of subjects, and may help to predict the response to treatment with specific drugs in an individual subject.

In empirical research the origin of the scientific hypotheses is subordinate to the testing of the hypothesis. These hypotheses must have theoretical validity, but may have come into existence as the result of a lost love, a lost job or as the result of a creative search process. The relevant hypothesis is then subjected to an empirical test to examine her legitimacy. In other words, an empirical scientific experiment is decisive by which a supposed theoretical relationship can be freed (or not) out of her hypothetical existence.

In this exploratory part we want to look - by means of a theoretically driven search process - if changes in combinations of biological variables may come to a better prediction of the Lybrido or Lybridos responses. This iterative search process will result in one new independent variable, which

has to be tested in an independent experiment on its efficacy for the Lybrido and Lybridos responses.

In extension to our earlier search process which determined the first version of the demarcation formula, other biomarkers will be added and investigated to assess in an iterative search process if the demarcation formula can be improved. These biomarkers of interest are:

- Dopamine receptor genotype polymorphism (D1-D5 receptor) and the dopamine transporter
- All serotonin receptors
- CRH/ AVP/ GC/ MC/ POMC receptor genes
- NET, NANC
- Oxytocin receptors
- Prolactin gene polymorphism
- STAR, Cytochrome P450 enzymes, DHEA, DHT, SHBG gene polymorphism, SHBG receptor polymorphism, aromatase/5-alpha-reductase
- NO synthase, PDE5 enzymes

A blood sample will be collected to determine the potential clinical utility of genetic analysis (microarray chips to identify alleles that are correlated with a trait/ multiple SNP analysis/tandem repeats et cetera) for the prediction of the response to treatment with Lybrido(s).

2.2 Hypotheses

2.2.1 Validation of existing demarcation formula

In the present study, the existing demarcation formula will be validated. Its predictive power for Lybrido and Lybridos sensitivity will be measured using the subjects number of satisfying sexual events on Lybrido and Lybridos as compared to Placebo in the domestic setting in 150 healthy female subjects with FSIAD.

- Women with a low sensitivity (as compared to high sensitivity) for sexual cues as determined by the present demarcation formula will have a statistically significant higher number of satisfying sexual events in the Lybrido regime as compared to the placebo and Lybridos treatments;
- Women with a high sensitivity (as compared to low sensitivity) for sexual cues as determined by the present demarcation formula, will have a significantly higher number of satisfying sexual events in the Lybridos regime as compared to the placebo and Lybrido regimes.

2.2.2 Testing of altered demarcation formula

The demarcation formula could be altered and improved with new biological markers using essentially the same iterative process as described in section 2.1.5.2, using the first 75 subjects who

complete this study. This altered demarcation formula will be tested in the second set of 75 subjects who complete the study, in the same way as the existing demarcation formula is validated above.

2.2.2.1 Primary hypotheses explorative part

- Women with a low sensitivity (as compared to high sensitive women) for sexual cues - as determined by the renewed demarcation formula - will have a higher number of satisfactory sexual events during treatment with Lybrido as compared with Placebo and Lybridos;
- The other way around, women with a high sensitivity (as compared to low sensitive women) for sexual cues will have a higher number of satisfactory sexual events during treatment with Lybridos as compared with Placebo and Lybrido;

Moreover,

- Women with a low sensitive system for sexual cues as determined with the new demarcation formula, will have a higher number of satisfactory sexual events during treatment with Lybrido in comparison with placebo and Lybridos, than women with a low sensitive system established by the original formula;
- Women with a high sensitive system for sexual cues as determined with the new demarcation formula, will have a higher number of satisfactory sexual events during treatment with Lybridos in comparison with placebo and Lybrido, than women with a high sensitive system established by the original formula.

2.3 Benefits and Risks

Use of Lybrido and Lybridos is expected to improve the level of sexual satisfaction. Women who are suffering from FSIAD do not have healthy sexual relationships. This often causes personal distress and/or strain on the relationship with their partner and often influences their self-esteem and perception of self-worth in a negative way. These women want to (re)find their joy in sexual activity. Both drugs are expected to enhance sexual motivation and arousal and to improve the pleasure associated with sexual activity and thus relieve personal distress and relational strain due to sexual problems. The correct allocation to the right drug treatment regimen is greatly increases the likelihood of a positive outcome of each of the treatments. Validation and improvement of the demarcation formula therefore contributes to an improvement in the welfare of patients.

Lybrido

Dosed separately, testosterone and sildenafil administration at the doses and frequency proposed for this study are not known to result in any serious health risks. Of particular note, the study medication will be taken on a per need basis, with an average estimated frequency of around 2 to 3 times per week. No significant adverse events (AEs) have been observed when Lybrido was administered in previous clinical studies. The maximum amount of testosterone (0.5 mg) administered in a dose of Lybrido roughly corresponds to what women produce endogenously per day (Greep and Astwood, 1973) . After administration of Lybrido, testosterone increases to supra-physiological levels for only a very short period of time and quickly returns to baseline levels

within 3 hours. In contrast to this, continuous androgen administration chronically raises testosterone levels to supra-physiological levels, which can result in masculinization effects such as hirsutism; it has also been linked to an increased risk of developing specific types of cancer and adverse cardiovascular effects. Lybrido, however, only contains a very modest amount of testosterone, and in this study, the maximum amount of testosterone administered will be 0.5 mg 8 times per 2 weeks; this level of testosterone exposure is significantly less than levels that have been associated with the above-mentioned side effects. Hence, the suggested dose and dosing regimen for testosterone, as a component of Lybrido, is considered to be safe.

Sildenafil has been combined with testosterone-containing gels or patches in previous studies and has been well tolerated without any significant AEs or interactions being reported; see the Investigator's Brochure for Lybrido for additional information. The most common AEs were minor headaches and blushing. More rarely, nausea, dyspepsia, palpitations, nasal congestion, rashes, and changes in vision have been reported.

Data on the effect of sildenafil and testosterone on oral contraceptives is lacking. For this reason, participants on oral contraceptives will be instructed to use a second contraceptive method (double barrier). All participants will be instructed not to become pregnant during the study.

Clinically relevant abnormalities e.g. in serum chemistry may be noticed, in which case the investigator may request a medical specialist for advice. If the specialist confirms that medical treatment is necessary, the participant's physician will be informed. This procedure is mandatory and will be explained to subjects in the Informed Consent form prior to enrollment.

Lybridos

Dosed separately, testosterone and buspirone administration at the doses and frequency proposed for this study are not known to result in any serious health risks. Of particular note, the study medication will be taken on a per need basis, with an average estimated frequency of around 2 to 3 times per week. No significant adverse events (AEs) have been observed when Lybridos (10 mg buspiron) was administered in previous clinical studies. The maximum amount of testosterone (0.5 mg) administered in a dose of Lybridos roughly corresponds to what women produce endogenously per day (Greep and Astwood, 1973). After administration of Lybridos, testosterone increases to supra-physiological levels for only a very short period of time and quickly returns to baseline levels within 3 hours. In contrast to this, continuous androgen administration chronically raises testosterone levels to supra-physiological levels, which can result in masculinization effects such as hirsutism; it has also been linked to an increased risk of developing specific types of cancer as well as adverse cardiovascular effects. Lybridos, however, only contains a very modest amount of testosterone, and in this study, the maximum amount of testosterone administered will be 0.5 mg 8 times per 2 weeks, for a total duration of 4 weeks; this level of testosterone exposure is significantly less than levels that have been associated with the above-mentioned side effects. Hence, the suggested dose and dosing regimen for testosterone, as a component of Lybridos, is considered to be safe.

The combined use of sublingual testosterone and buspirone is considered safe because buspirone efficacy is not influenced when testosterone is given concomitantly (Fernandez-Guasti and Picazo, 1997). Moreover, the dose of buspirone of 10 mg is at most two thirds of the starting daily dose and

less than a half (2/5) of the lightest weekly dose of buspirone prescribed for use as an anxiolytic drug. A previous home study (EB70) showed that Lybridos is well tolerated during 4 weeks on-demand use. See the Investigator's Brochure for Lybridos for additional information.

Data on the effect of buspirone and testosterone on oral contraceptives is lacking. For this reason, participants should be aware to use highly effective contraception during the study. All participants will be instructed not to become pregnant during the study.

Clinically relevant abnormalities e.g. in serum chemistry may be noticed, in which case the investigator may request a medical specialist for advice. If the specialist confirms that medical treatment is necessary, the participant's physician will be informed. This procedure is mandatory and will be explained to subjects in the Informed Consent form prior to enrollment.

3 INVESTIGATIONAL PLAN

3.2 Objectives

3.2.1 Primary Objective

- To validate the existing demarcation formula (consisting of psychometric and biological markers) which predicts the sensitivity to Lybrido or Lybridos in women with female sexual interest/arousal disorder (FSIAD with or without FOD as secondary diagnosis), measured by the number of satisfactory sexual events (in 150 subjects).

3.2.2 Secondary Objectives

- To identify in an iterative process additional psychometric and biological markers for the demarcation formula in order to increase predictive power for sensitivity to Lybrido or Lybridos in women with female sexual interest/arousal disorder (FSIAD with or without FOD as secondary diagnosis), measured by the number of satisfactory sexual events (in a first set of 75 subjects of the total amount of 150 subjects).

- To evaluate the altered demarcation formula in women with female sexual interest/arousal disorder (FSIAD with or without FOD as secondary diagnosis), measured by the number of satisfactory sexual events (in a second set of 75 subjects).

3.3 Endpoints

3.3.1 Primary Endpoint

- The primary endpoint is the change from placebo in frequency of satisfactory sexual events, following study medication intake, measured by the Sexual Event Diary (SED), item 4 (this endpoint is used for both the primary and secondary objective above)

3.3.2 Exploratory Endpoints

- Change from placebo in experienced sexually-related personal distress, measured by the Female Sexual Distress Scale-Revised (FSDS-R), specifically item 13.
- Evaluation of meaningful improvement during treatment period, measured by the single item Patient's Global Impression of Improvement (PGI-I)
- Evaluation of meaningful benefit of study medication during treatment period, measured by the single item Patient Benefit Evaluation (PBE)
- Change from placebo in frequency of orgasms, following medication intake, measured by the SED
- Change from placebo in sexual desire, following medication intake, measured by the SED
- Change from placebo in physical arousal, following medication intake, measured by the SED
- Change from placebo in mental arousal, following medication intake, measured by the SED
- Change from placebo in sexual pleasure, following medication intake, measured by the SED

3.3.3 Safety evaluation

- Adverse events
- Physical examination (including vital signs)
- Signs of hyperandrogenism
- Laboratory assessments (including blood chemistry, hematology and hormone levels)

3.4 Study Design

This is a double-blind, randomized, cross-over, placebo-controlled study with a 2-week single-blind placebo run-in period, a 6-week double-blind treatment period, and a 1-week follow-up period. The double-blind treatment period consists of a Lybrido (0,50 mg testosterone + 50 mg sildenafil) regime (duration 2 weeks), a Lybridos (0,50 mg testosterone + 10 mg buspirone) regime (duration 2 weeks), and a placebo regime (duration 2 weeks). Each regimen will be separated by at least a 2-day wash out period. Each subject completes the 2-week treatment periods in randomized order following a Williams design with 6 sequences. Randomization will be performed at least 24 hours before visit 2, but not at visit 1. This will ensure that the allocation of medication kit-number to a subject according to the randomization list is done in time to be able to dispense the medication on visit 1.

Subjects will visit the study site a total of 7 times: 1 screening visit, 1 start-up visit, 4 study regimen follow-up visits, and 1 final follow-up visit. During the start-up visit and study regimen follow-up visits, the subject's sexual functioning will be evaluated, subject's health will be monitored and study medication will be dispensed (no medication will be dispensed on the last study regimen follow-up visit, V6). Sexual satisfaction will be measured using several questionnaires, some of which will be completed at home following a sexual event. At the start-up visit, the subject will see a psycho-educational DVD with theoretical background information about sexual functioning and fantasizing exercises, to keep the basic knowledge of the subjects about sexuality on an approximately equal level. After watching the DVD the information will be discussed with a psychologist or similar healthcare provider.

The study is divided into a validation part and an exploratory part using two populations. In the validation part, the existing demarcation formula, described elsewhere, is validated in 150 subjects with FSIAD. In the exploratory part, the possibility of improving the demarcation formula is examined using the first 75 of these 150 patients, and then validated in the last 75 patients.

3.5 Subject Selection

3.5.1 Number of Subjects

Approximately 150 healthy female subjects with FSIAD will be enrolled and randomized to study treatment.

3.5.2 Inclusion Criteria

Criteria for inclusion

Subjects must meet *all* of the following criteria:

1. Provision of written informed consent;

2. Females between 18 and 70 years of age, inclusive, pre- or postmenopausal, with FSIAD (comorbidity with female orgasmic disorder [FOD]; only as secondary diagnosis) is allowed. The diagnosis of FSIAD will be established by a trained health care professional;

3. Be involved in a stable, communicative, monogamous relationship and have a sexually functional partner who will be at home for the majority of the study duration;

4. Healthy with normal medical history, physical examination, laboratory values, and vital signs; exceptions may be made if the investigator considers an abnormality to be clinically irrelevant;

5. Use of highly effective contraception.

3.5.3 DSM-5 Criteria for FSD

FSD refers to various disturbances or impairments of sexual functioning, of which low sexual desire and sexual arousal problems are the most common among women.

In May 2013 the DSM-IV-TR was replaced by the DSM-5. Up until then a problem with sexual desire was classified as HSDD, and a problem with sexual arousal was classified as FSAD. Because of the significant overlap between arousal and desire, recommendations were done for revision of the DSM-IV-TR criteria. In the DSM-5 these two sexual problems are combined into the new female sexual interest and arousal Disorder (FSIAD).

In this study subjects with desire and/or arousal problems will be diagnosed according to the DSM-5 criteria for FSIAD. To qualify for FSIAD according to the DSM-5, three of the six indicators mentioned in criterion A should be met. For this study two of the three indicators of criterion A that should be met these are; 'absent/reduced interest in sexual activity' and 'absent/reduced sexual/erotic thoughts or fantasies'. These indicators match with the main indicators of the formerly used DSM-IV-TR diagnosis of HSDD. Because of this, all women who meet these criteria are automatically diagnosed as having HSDD (but not vice versa).

For this study, subjects may be enrolled when FSIAD (with or without female orgasmic disorder) is diagnosed by a trained health care professional.

Diagnostic Criteria for FSIAD

A. Lack of, or significantly reduced, sexual interest/arousal, as manifested by at least three of the following:
   1. Absent/reduced interest in sexual activity
   2. Absent/reduced sexual erotic thoughts or fantasies
   3. No/reduced initiation of sexual activity, and typically unreceptive to a partner's attempts to initiate.
   4. Absent/reduced sexual excitement/pleasure during sexual activity in almost all or all (approximately 75%-100%) sexual encounters (in identified situational contexts of, if generalized, in all contexts).
   5. Absent, reduced sexual interest/arousal in response to any internal or external sexual/ erotic cues (e.g., written, verbal, visual).
   6. Absent/reduced genital or nongenital sensations during sexual activity in almost all of all (approximately 75%-100%) sexual encounters (in identified situational contexts or, if generalized, in all contexts).
B. The symptoms in Criterion A have persisted for a minimum duration of approximately 6 months.
C. The symptoms in Criterion A cause clinically significant distress in the individual.
D. The sexual dysfunction is not better explained by a nonsexual mental disorder or as consequence of severe relationship distress (e.g. partner violence) or other significant stressors and is not attributable to the effects of a substance/medication or another medical condition.

Lifelong: The disturbance had been present since the individual became sexually active.
Acquired: The disturbance began after a period of relatively normal sexual function.

Generalized: Not limited to certain types of stimulation, situations, or partners.
Situational: Only occurs with certain types of stimulation, situations, or partners.

Mild: Evidence of mild distress over the symptoms in Criterion A.
Moderate: Evidence of moderate distress over the symptoms in Criterion A.
Severe: Evidence of severe or extreme distress over the symptoms in Criterion A.

Diagnostic Criteria for FOD

A. Presence of either of the following symptoms and experienced on almost all or all (approximately 75%-100%) occasions of sexual activity (in identified situational contexts or, if generalized, in all contexts):
   1. Marked delay in, marked infrequency of, or absence of orgasm.

2. Markedly reduced intensity of orgasmic sensations

B. The symptoms in criterion A have persisted for a minimum duration of approximately 6 months.
C. The symptoms in Criterion A cause clinically significant distress in the individual.
D. The sexual dysfunction is not better explained by a nonsexual mental disorder or as a consequence of severe relationship distress (e.g., partner violence) or other significant stressors and is not attributable to the effects of a substance/medication or another medical condition.

Lifelong: The disturbance has been present since the individual became sexually active.
Acquired: The disturbance began after a period of relatively normal sexual function.

Generalized: Not limited to certain types of stimulation, situations, or partners.
Situational: Only occurs with certain types of stimulation, situations, or partners.

Specify if never experienced an orgasm under any situation

Mild: Evidence of mild distress over the symptoms in Criterion A.
Moderate: Evidence of moderate distress over the symptoms in Criterion A.
Severe: Evidence of severe or extreme distress over the symptoms in Criterion A.

3.5.4 Exclusion Criteria

Criteria for exclusion

Subjects who meet *any* of the following criteria are not eligible to participate in the study:

<u>Cardiovascular Conditions</u>

1. Any underlying cardiovascular condition, including unstable angina pectoris, that would preclude sexual activity;

2. Systolic blood pressure ≥ 140 mmHg and/or diastolic blood pressure ≥ 90 mmHg (supine blood pressure). For subjects ≥ 60 years old and without diabetes mellitus, familial hypercholesterolemia, or cardiovascular disease: systolic blood pressure ≥ 160 mmHg and/or diastolic blood pressure ≥ 90 mmHg;

3. Systolic blood pressure ≤ 90 mmHg and/or diastolic blood pressure ≤ 50 mmHg (supine blood pressure);

<u>Gynecological and Obstetric Conditions</u>

4. Use of any contraceptive containing anti-androgens (e.g. Cyproteron acetate) or (anti)androgenic progestogens (drospirenone, dienogest, chlormadinone acetate and norgestrel);

5. Use of any contraceptive or hormone replacement therapy (HRT) containing more than 50 µg/day of estrogen;

6. Pregnancy or intention to become pregnant during this study (Note: A urine pregnancy test will be performed in all women of child bearing potential prior to the administration of study medications);

7. Lactating or delivery in the previous 6 months prior to signing Informed Consent Form;

8. History of bilateral oophorectomy;

9. Other unexplained gynecological complaints, such as clinically relevant abnormal uterine bleeding patterns;

10. Perimenopausal status (cycle shortening/irregular menstrual bleeding in the last 12 consecutive months and/or occurrence of vasomotor symptoms (e.g. hot flashes, night sweating) and/or FSH levels (>40 IU/L) for women from age 40 onwards; in women with a history of hysterectomy perimenopausality can be assessed by FSH levels (> 40 IU/L) and/or vasomotor symptoms);

Other Medical Conditions

11. Liver and/or renal insufficiency (aspartate aminotransferase, alanine aminotransferase and gamma glutamyltransferase > 3 times the upper limit of normal and/or estimated glomerular filtration rate (eGFR) **<** 60.00 mL/min based on the Cockcroft-Gault formula);

12. Any current endocrine disease or endocrinopathy (e.g. uncontrolled thyroid function) as determined by medical history, basic physical examination and/or laboratory values significantly outside normal range of the central laboratory; or uncontrolled diabetes mellitus(HbA1c > 7.5%);

13. Free- and/or total testosterone levels outside the upper limit of the reference range of the central laboratory;

14. Any current clinically relevant neurological disease which, in the opinion of the investigator, would compromise the validity of study results or which exclude from use of sildenafil, buspirone and/or testosterone;

15. History of hormone-dependent malignancy (including all types of breast cancer);

16. Positive test result for immunodeficiency virus, hepatitis B, or hepatitis C (acute and chronic hepatitis infection);

Psychological/Psychiatric Factors

17. History of (childhood) sexual abuse that, in the opinion of the investigator, could result in negative psychological effects when testosterone is administered;

18. (Psychotherapeutic and/or pharmacological treatment for) a psychiatric disorder (other than those under inclusion criterion 6) that, in the opinion of the investigator, would compromise the validity of study results or which could be a contraindication for sildenafil, buspirone and/or testosterone use;

19. Current psychotherapeutic treatment for female sexual dysfunction;

20. Current genito-pelvic pain/ penetration disorder according to the Diagnostic and Statistical Manual of Mental Disorders, fifth edition (DSM-5);

21. A substance abuse disorder that, in the opinion of the investigator, is likely to affect the subject's ability to complete the study or precludes the subject's participation in the study;

Concomitant Medications

22. Use of potent CYP3A4 inhibitors (eg, ritonavir, ketoconazole, itraconazole clarithromycin, erythromycin and saquinavir);

23. Use of potent CYP3A4 inducers (eg, carbamazepine, phenytoin, phenobarbital, St John's wort, rifampin);

24. Use of antidepressants including SSRIs, tricyclic and other;

25. Use of any other medication that interferes with study medication (eg, triptans, monoamine oxidase [MAO] inhibitors [includes classic MAO inhibitors and linezolid] and spironolactone);

26. Use of medication (including herbs) that would compromise the validity of study results;

27. Use of testosterone therapy within 6 months before study entry prior to signing the Informed Consent Form;

General

28. Illiteracy, unwillingness, or inability to follow study procedures;

29. Participation in other clinical trials within the last 30 days;

30. Any other clinically significant abnormality or condition which, in the opinion of the investigator, might interfere with the participant's ability to provide informed consent or comply with study instructions, compromise the validity of study results, or be a contraindication for buspirone, and/or sildenafil and/or testosterone use.

Restrictions

Only concomitant medications which the investigator has deemed safe may be used during the study. The use of alcohol is allowed, unless contraindicated due to the use of allowed concomitant medication; excessive drinking is discouraged. There is evidence that alcohol will not induce negative side effects or negatively influence sildenafil, buspirone or testosterone.[58, 59, 60] Subjects on acceptable oral contraceptives will be instructed to use highly effective contraception from the screening visit (V0) until the final follow-up visit (V6).

4 STUDY ACTIVITIES AND EVALUATION METHODS

4.1.1 Study Medication & Route of Administration

Lybrido

Lybrido is a fixed-dose combination of testosterone and sildenafil citrate. The drug product is a 9 mm, round, biconvex, white, menthol-flavored tablet for sublingual administration. The outer, polymeric, film coating contains testosterone (0.5 mg) that is released immediately after sublingual administration. The inner core of the tablet contains sildenafil (50 mg). This inner core has a polymeric coating designed to delay release of the sildenafil for approximately 2.5 hours; after this time delay, the sildenafil is released immediately (i.e., it is not a sustained release).

Lybridos

Lybridos is a fixed-dose combination of testosterone and buspirone hydrochloride. The drug product is a 9 mm, round, biconvex, white, menthol-flavored tablet for sublingual and oral administration. The outer, polymeric, film coating contains testosterone (0.5 mg) that is released immediately after sublingual administration. The inner core of the tablet contains buspirone hydrochloride (10 mg). This inner core has a polymeric coating designed to delay release of the buspirone for approximately 2.5 hours; after this time delay, the buspirone is released immediately (i.e., it is not a sustained release).

Placebo

The placebo has the exact same appearance and flavor as Lybrido and Lybridos.

Dosing Instructions

Subjects will be instructed to keep the tablet under their tongue for 90 seconds and swish the produced saliva beneath their tongues during these 90 seconds, then to swallow the tablet as a whole thereafter, without chewing or otherwise disrupting the dosage form.

A total of 8 doses will be provided for each medication regime; 8 doses Lybrido, 8 doses Lybridos, and 2x (placebo run in and placebo in the double blind regime) 8 doses placebo. Subjects are required to take a minimum of 4 doses over each 2-week treatment regime (2 dose/week). The other 4 doses may be taken as desired (i.e., “on demand”) throughout the 2-week treatment regime; dosing is permitted every day until all doses are used. If within a regime a subject uses all doses, medication will not be resupplied.

4.1.2 Packaging, Labeling, and Storage of Study Medication

All study medications will be supplied in labeled, high density polyethylene bottles of 8 tablets, which should be stored at 25ºC (77ºF); temperature excursions within the range of 15ºC to 30ºC (59ºF to 86ºF) are permitted.

All study medication must be stored in a lockable area with restricted access, and a temperature log or chart must be maintained to monitor the environment.

The following information will be included on the study medication labels: protocol number, kit identification number, batch number, description of contents, dosing instructions, storage instructions, subject identification number, identification of principal investigator, and sponsor contact information.

When study medication is dispensed, prior to each medication regime, the medication will be taken from the original bottle and put in a designated bottle, fitted with a MEMSCap, by the patient. (See section 7.4. for more information on the MEMSCap system and the use of designated bottles). This will be supervised by a study physician.

4.2 Enrollment and randomization

Subjects will be enrolled after meeting the inclusion/exclusion criteria and within 12 weeks after screening (V0) (before Visit 1). Thereafter they complete a 2-week single blind placebo regime. Up until visit 2, subjects will only receive placebo (run in period). However, since all medication is packed into a single kit, subjects will be randomized prior to V1. The placebo run-in period is followed by a double blind 6-week treatment period. This treatment period consists of a 2-week Lybrido, a 2-week Lybridos, and a 2-week placebo regime. Each subject completes the treatment regime in a randomized order. The randomization schedule follows a Williams design with 6 sequences. The list with the randomized medication kit-numbers will be designed by an independent statistician. Subjects will be randomized, at least 24 hours before V1, using the electronic CRF. One of the unique medication code numbers from the randomization schedule will be coupled with the randomized subject.

A subject identification code will be used to keep the data anonymous. The code will consist of a 2-digit site code, a 4-digit subject screening number and a 3-digit randomization number (e.g., 01-0123-001).Access to the key for the code (subject identification log) will be restricted and safeguarded by the Medical Monitor. If a subject discontinues from the study, the unique subject identification code will not be used again.

In alignment with the intention-to-treat analysis, subjects who have received study medication will not be replaced. A subject will be replaced if she has not yet received any study medication (e.g., the subject decides to stop before V1) but only during the enrolment period (a duration of 16 weeks) of the study. When the enrolment period has ceased, subjects will not be enrolled or replaced.

4.3 Study Procedures

Approximately 150 healthy female subjects with FSIAD will be enrolled in the study. After written informed consent has been obtained and inclusion and exclusion criteria have been met, subjects will be enrolled in the study. An overview of the study visits is provided in Table 1; a detailed schedule of the study procedures is provided in Appendix 2.

Table 1: Overview of Study Visits

Study Visit	Assessment (Duration)
Visit 0	Screening (3 hours)
Visit 1	Start-up visit (1,5 hour)
Visits 2, 3, 4, and 5	Study regimen follow-up (1 hour)
Visits 6	Final follow-up (1 hour)

4.3.1 Placebo Run-in Period

All subjects will begin the study with a 2-week single blind, placebo run-in period, before starting the double-blind treatment period. The placebo run-in period is organized exactly the same as the treatment period and is intended to minimize possible placebo effects.

4.3.2 Visits

4.3.2.1 V0: Screening Visit

Written informed consent will be obtained prior to any enrollment procedure.

The screening visit (V0) will include the following (not necessarily in this order, except giving informed consent which must always be done first):

1. Giving written informed consent
2. Interview with experienced psychologist/appropriately trained healthcare provider to assess sexual history and diagnose FSIAD with or without female orgasmic disorder .
3. Assessment of alcohol consumption and smoking history
4. Physical examination, including medical, gynecological, and surgical history, vital signs (including supine blood pressure and heart rate), 12-lead ECG (supine), monitoring for signs of hyperandrogenism (see Section 4.3.8.2)
5. Collection of blood sample for routine hematology, chemistry, steroid hormone levels, and serology (Appendix 3)
6. Urine drug screening (Section 4.3.6)
7. Routine urinalysis (Section 4.3.6.1)
8. Urine pregnancy test
9. Completion of the following questionnaires: Sexual Anamnesis Questionnaire-Diagnostic (SAQ-D), Sexual Motivation Questionnaire (SMQ), Female Sexual Distress Scale revised (FSDS-R)

Table 2: Measurements and Activities at V0

V0: Enrollment/Screening
Written informed consent
Inclusion/exclusion criteria
Demographics
Medical history
Interview psychologist/trained healthcare provider
Height
Body weight
Vital signs, ECG
Physical examination
Hyperandrogenism monitoring
Pregnancy test
Urine drug screen
Urinalysis: dipstick
Hematology and chemistry assessment
Questionnaires: SAQ-D, SMQ, FSDS-R
Concomitant medications

SAQ-D = Sexual Anamnesis Questionnaire-Diagnostic; SMQ = Sexual Motivation Questionnaire ; Female Sexual Distress Scale revised (FSDS-R)

If a subject is not enrolled for CD001 within 12 weeks after screening (V0) but is still eligible for inclusion consideration, the subject must have initial safety lab work repeated; chemistry, hematology, and hormone levels. Additionally, any other changes from baseline in the subject's health will be documented (see Table 3 for details).

Table 3: Measurements and Activities >12 weeks after screening

V0: Rescreening
Update Inclusion/exclusion criteria
Update Demographics
Update Medical history [1]
Pregnancy test (women of child-bearing potential)
Update Interview psychologist/trained healthcare provider [2]
Hematology, chemistry, and hormones assessment

Questionnaires: SAQ-D, FSDS-R, SMQ [3]

Update Concomitant medications [4]

SAQ-D = Sexual Anamnesis Questionnaire-Diagnostic;; SMQ = Sexual Motivation Questionnaire; Female Sexual Distress Scale revised (FSDS-R)

1 Check the medical history for significant and/or relevant changes or events (e.g., Urinary Tract Infection (UTI); recent surgery); in case of any event or change please repeat the appropriate screening measurements and/or activities (e.g. physical- ,or gynecological examination, urinalysis).
2 Check if subjects have had significant change in sexual functioning (e.g. due to life event).
3 If subjects report change in sexual functioning, SAQ-D, FSDS-R and SMQ must be filled out again.
4 Check if subjects concomitant medication has changed (e.g. because of UTI, recent surgery).

4.3.2.2 V1: Start-up Visit

During the start-up visit (V1), subjects will be examined for clinically significant deviations from normal in vital signs. AEs (as defined in Section 5) and concomitant medications will be recorded, monitoring for signs of hyperandrogenism and a urine pregnancy test will be done for all women of child-bearing capability. Also a hand scan of the left hand will be made to measure digit length. The subject will see a psycho-educational DVD with theoretical background information about sexual functioning and fantasizing exercises. After watching the DVD the information will be discussed with a psychologist or similar healthcare provider. Instructions will be given on how to complete the SED and the weekly diary. Subjects will fill out the FSDS-R questionnaire. The first medication bottle will be dispensed to subjects. A blood sample will be taken for the assessment of the biological markers (CAG repeat length of the androgen receptor (AR) gene, SERT, 5-HT1a genotype, and several other markers using microarray chips) if these results are not current within past 12 months and obtainable as part of medical history.

Subjects are expected to complete the full two (2) weeks of the placebo run in period before participating in Visit 2. Subjects may be scheduled for V2 from one day after the end of the placebo run in period and up to one (1) week after the end of that period.

Table 4: Measurements and Activities at V1

**V1: Start-up Visit**
Inclusion/exclusion criteria
Pregnancy test
Psychoeducational DVD
2D:4D measurement, left hand
Vital signs
Collection of blood samples for assessment of biological markers
Hyperandrogenism monitoring

V1: Start-up Visit
Instructions for SED and weekly diary
Study medication dispensing[a]
Concomitant medications
Adverse and serious adverse events
Questionnaires: FSDS-R
Randomisation (prior to V1)

Notes: ; FSDS-R=Female Sexual Distress Scale –Revised; SED= Sexual Event Diary;
[a] Study medication dispensed at V1, V2, V3, and V4.

4.3.2.3 V2, V3, V4, and V5: Study Regimen Follow-up Visits

During the study regimen follow-up visits (V2, V3, V4, and V5), subjects will be examined for clinically significant deviations from normal in vital signs. Any AEs and use of concomitant medications will be recorded, monitoring for signs of hyperandrogenism and a urine pregnancy test will be done for women of child bearing potential. During the study regimen follow-up visits, subjects will fill out the FSDS-R,PGI-I and PBE questionnaires and the psychologist/designee will conduct an interview to assess improvement. At visit 1, 2, 3, and 4 subjects will receive medication for 2 weeks.

The visits should be scheduled from one (1) day after the end of the 2-week treatment period up to three (3) weeks after the end of that period (see Appendix 1: Study Timelines and Windows).

Table 5: Measurements and Activities at V2, V3, V4, and V5

V2, V3, V4, and V5: Regimen Follow-up
Inclusion/exclusion criteria
Pregnancy test
Vital signs
Hyperandrogenism monitoring
Interview psychologist/sexologist
Concomitant medications
Adverse and serious adverse events
Study medication dispensing[a]
Study medication interim count[b]
Questionnaires: FSDS-R, PGI-I and PBE

V2, V3, V4, and V5: Regimen Follow-up

Notes: FSDS-R= Female Sexual Distress Scale –Revised; PBE = Patient Benefit Evaluation; PGI-I = Patient's Global Impression of Improvement

[a] Study medication dispensed at V2, V3, and V4.

[b] Study medication interim count at V3, V4, and V5

4.3.2.4 V6: Final Follow-up Visit

During the final follow-up visit (V6), subjects will receive a physical examination and will be assessed for clinically significant deviations from normal in vital signs; a blood sample will be collected for hematology and clinical chemistry assessments (Appendix 3). Any AEs and use of concomitant medications will be recorded, monitoring for signs of hyperandrogenism and a urine pregnancy test will be done for women of child bearing potential. Subjects will fill out the FSDS-R, and the psychologist/designee will conduct an interview to assess improvement. Visit 6 should be scheduled one (1) week after completion of Visit 5, +/- one (1) week.

Table 6: Measurements and Activities at V6

V6: Final follow-up
Interview psychologist/sexologist
Physical examination
Body weight
Pregnancy test
Vital signs
Hyperandrogenism monitoring
Hematology safety
Clinical chemistry safety
Concomitant medications
Adverse and serious adverse events
Questionnaires: FSDS-R

Notes: ; FSDS-R= Female Sexual Distress Scale –Revised;

4.3.2.5 Home: On-going Measurements

Subjects will be required to fill out 2 kind of diaries, a weekly diary (Section 4.3.4.2) and the SED (Section 4.3.4.3), accessing a secure website using a home computer. The weekly diary is a

structured questionnaire that has to be completed once a week. The SED is a questionnaire that has to be completed within 24 hours of a sexual event (this includes masturbation). Subjects are requested to have at least two sexual events a week.

4.3.3 Description of Measurements

4.3.3.1 Hand Scan Measuring Digit Ratio

Subjects will be asked to remove any jewelry or rings that would interfere with obtaining finger length measurements (subjects who are unable to remove rings will be asked to slide the jewelry up the digit as far as possible to allow documentation of the digit/hand intersection). Each subject's left hand will be scanned using a standard photo scanner at a resolution of 200 dpi. The subject will be instructed to place the ventral surface of their hand lightly on the surface of the scanner, with the second to fifth fingers held parallel and the tip of the middle finger aligned with the wrist and elbow. Scanned hand images will be analyzed using computer-assisted image analysis software. A built-in measurement tool (accurate to 0.1 mm) will be used to measure digit length. The length of the second and fourth digit of the left hand will be measured from the ventral proximal crease of the digit to the tip.

4.3.4 Questionnaires

4.3.4.1 Sexual Anamnesis Questionnaire-Diagnostic

The SAQ-D is a 43-item self-report questionnaire (Appendix 4). It is designed to assess the different aspects of sexual functioning (desire, arousal, orgasm, and pain corresponding to the DSM-IV-TR), presence of childhood sexual abuse, quality of partner relationship, and the presence of comorbidity factors. Several items of this questionnaire are used in the demarcation formula.

4.3.4.2 Weekly Diary

Subjects will be instructed to fill out the weekly diary once a week (Appendix 6). This self-report questionnaire includes 7 multiple choice questions concerning sexual desire, arousal, and inhibition felt during the past week.

4.3.4.3 Sexual Event Diary

The SED is a self-report questionnaire (Appendix 7). It is designed to assess the sexual satisfaction of a recent sexual event. Subjects are instructed to fill out the SED within 24 hours of any sexual event they experience.

4.3.4.4 Sexual Motivation Questionnaire

The SMQ is a 40-item self-report trait questionnaire using 5-point Likert scale items (Appendix 11). This questionnaire assesses specific aspects of sexual motivation. Several items of this questionnaire are used in the demarcation formula.

4.3.4.5 Female Sexual Distress Scale –Revised

The Female Sexual Distress Scale –revised is a 13-item standardized, quantitative scale (Appendix 12). This questionnaire measures sexually-related distress in women.

4.3.4.6 Patient's Global Impression of Improvement (PGI-I)

The PGI-I is used to determine if the subject feels that she has experienced any meaningful improvement during the treatment period (Appendix 14).

"How is your condition – meaning decreased sexual desire and feeling bothered by it – today compared with when you started study medication?" [very much improved 1 to 7 very much worse]

4.3.4.7 Patient Benefit Evaluation (PBE)

The PBE is used to determine if the subject feels that she has had any meaningful benefit of the study medication (Appendix 15).

4.3.5 Program for psycho-education

The program for psychological counseling will be carried out by digital versatile disk (DVD).

The DVD contains a 30-minute interview with an experienced psychologist. Topics include sexual functioning and the female response cycle, bodily changes during sexual arousal, differences between men and women, problems in sexual functioning, and misunderstandings. There is also a 7-minute training module on sexual fantasizing.

The entire DVD will only be shown at Visit 1. Afterwards, the subject and the psychologist will discuss the psycho-educational parts.

4.3.6 Laboratory Parameters

Clinical chemistry, hematology, serology, and steroid will be performed by a qualified central laboratory in the Netherlands. At the research site in Almere the total amount of blood to be drawn during this study is 47 ml (21 ml at screening, 16 ml at follow up, and 15 ml for assessment of genetic markers). Because of a different laboratory where the blood from the research site in Utrecht will be analysed, the total amount to be drawn in Utrecht is 65,5 ml (34,5 ml at screening, 16 ml at follow up, and 15 ml).

Urine drug screen, routine urinalysis (as listed below), and urine pregnancy testing will be performed at the clinical sites.

Each parameter outside the normal range will be designated as high or low on the laboratory documentation. The investigator will determine if "outside the normal range" values are clinically relevant; if so, an assessment will be provided.

4.3.6.1 Urine Drug Screening

A fresh urine sample (10-20 mL) will be collected for urinary drug screening. Urinary drug screening for amphetamines, barbiturates, benzodiazepines, cocaine, methamphetamines, MDMA, morphine, methadone, tricyclic antidepressants and THC will be performed qualitatively at the screening visit (V0).

4.3.6.2 Urinalysis

During screening (V0), a fresh urine sample (20 mL) will be collected and analyzed with a dipstick for bilirubin, blood, glucose, ketones, pH, protein, specific gravity, urobilinogen, leukocytes, and nitrite.

For each parameter testing +, the investigator will determine if the results are clinically relevant; if so, an assessment will be provided.

4.3.6.3 Urine Pregnancy Test

A test for urinary human chorionic gonadotropin will be done at each visit to the site for women of child-bearing capability.

4.3.6.4 Hematology and Blood Chemistry

Blood samples will be collected for hematology and blood chemistry safety assessments at the screening visit (V0) and at the final follow up visit (V6). See Appendix 3 for a list of assessments performed at each of these visits. For potential subjects who exceed the 12 week window between V0 and V1 and who continue to be eligible for inclusion, hematology, chemistry and hormone levels will be drawn.

4.3.6.5 Luteinizing Hormone, Follicle Stimulating Hormone, Sex Steroids, Sex Hormone Binding Globulin, and Prolactin

Blood samples will be collected at screening (V0) to determine levels of the following: total testosterone, sex hormone binding globulin (SHBG), estradiol, luteinizing hormone, follicle stimulating hormone, and prolactin. Total testosterone and SHBG will also be determined at the final follow-up visits (V6).

4.3.6.6 Serology

Assessment of hepatitis B surface antigen, hepatitis C antibody, and HIV antibodies for HIV-1 and HIV-2 will be performed at screening (V0).

4.3.6.7 Biological markers

A blood sample will be collected at the start-up visit (V1) after randomization.

4.3.7 Safety and Tolerability Measurements Procedure

All subjects will be part of the safety and tolerability evaluation. Subjects will be monitored for vital signs, AEs, hematology, and blood chemistry during the study and on follow-up.

4.3.8 Safety Assessments

4.3.8.1 Vital Signs

Vital signs will be measured at all visits. Systolic blood pressure and diastolic blood pressure will be taken with the subject in a supine position for at least 5 minutes. For subjects whose initial BP reading is outside the specified acceptable range, one to two subsequent readings should be obtained. Two successive out-of-range readings disqualify subjects from participation. Heart rate

will also be measured. Subjects should be observed closely for any sign or report of dizziness or lightheadedness upon standing.

4.3.8.2 Hyperandrogenism Monitoring

Monitoring for the following signs of hyperandrogenism will be performed at all visits: hirsutism (terminal hair on the lip, chin, chest, abdomen, and back); acne; seborrhea; temporal balding; acanthosis nigricans; striae; thin skin or bruising; deepening of the voice; frontal (or crown) balding; increased muscle mass; or clitoromegaly (if reported by subject, length > 10 mm and/or index > 35 mm^2 is considered above normal).

If a subject reports one or more of the above symptoms, an extra blood sample will be collected to determine the serum total testosterone, SHBG, albumin level and calculated free testosterone. See Section 4.4.2 for study stopping rules regarding hyperandrogenism.

4.3.8.3 Electrocardiogram

A 12-lead ECG with a hard-copy output will be taken at the screening visit (V0). The screening ECG will be evaluated by the medical investigator. Heart rate, PR interval, QRS duration, QT interval, QTc interval (Bazett), and any abnormalities will be recorded. The Investigator/qualified designee will initial and date the copy to indicate agreement with tracing analysis or will re-interpret, initial and date.

4.3.8.4 Physical Examination

The physical examination performed at the screening visit (V0) will constitute the baseline measurement for the study period.

4.3.9 Adverse Events

Information on AEs will be gathered by spontaneous reporting in the weekly diary and at all visits to the site.

4.3.10 Food and Beverages

Moderate use of alcohol is allowed.

4.4 Study Termination

4.4.1 Regular Study Termination

The final follow-up visit (V6) is also the end-of-study visit. This visit will be scheduled to occur 1 week after the last study regimen follow-up visit (V5). A full safety assessment will be conducted (see Section 4.3.2.4 for details).

4.4.2 Premature Study Termination

Subjects may be withdrawn from the study for any of the following reasons:

1. At their own request, or at the request of their legally authorized representative

2. If, in the investigator's opinion, continuation in the study would be detrimental to the subject's well being
3. A significant protocol violation
4. Severe laboratory abnormality where continuation of participation could put the subject at risk
5. Hyperandrogenism, defined as one or more reported signs of hyperandrogenism (see Section 4.2.9.2) in combination with serum total testosterone levels or calculated free testosterone above the normal range
6. A subject who develops new angina or significant cardiovascular disease should be terminated from the study to avoid the potential emergency intake of nitrate therapy in close association with the intake of study medication
7. The treatment code is broken
8. The subject becomes pregnant
9. There is a change in partner availability (through termination of the relationship, travel, or other circumstances that remove the partner for an extended period of time)

Subjects who experience an intolerable AE or who become pregnant will be withdrawn from study treatment but will continue to be monitored until the AE has resolved or the outcome of the pregnancy has been determined.

When a subject terminates between Visit 1 and Visit 6, she should be asked to return to site for a safety blood draw (Chemistry, Hematology and Hormone) and documentation of any significant changes from her baseline assessments, AEs and/or SAEs.

4.5 Breaking the Blind

The blind may be broken for a subject only in exceptional circumstances. The only acceptable reason for requesting to break the blind by the investigator is that the information is necessary for medical management of the subject. The investigator must, whenever possible, contact the sponsor's Medical Monitor to provide details on the reason for wanting to break the code. If approved, the Medical Monitor will unblind the subject and reveal the randomization code to the inquiring investigator. This notification must include the appropriate documentation, signed and dated by the investigator and giving the reason for breaking the code. All further procedures undertaken with respect to circumstances related to breaking the blind must be confirmed in writing within 3 working days after breaking the blind. Subjects whose code has been broken must be discontinued from the clinical trial, and the reason for discontinuation must be documented.

The sponsor, together with the investigator and the responsible statistician (if applicable) will determine the implications for analysis of data from the subject. Appropriate documentation must be completed, signed, and dated by the responsible clinical project manager.

4.6 Interim Database Lock

This study will have one interim database lock. This interim database lock will take place on the first 75 subjects with FSIAD who complete the study. This is done so that on these subjects an altered/improved demarcation formula can be based. The data of the second set of 75 subjects with FSIAD who complete the study will be used to validate this altered demarcation formula.

5 ADVERSE EVENTS AND SERIOUS ADVERSE EVENTS

5.1 Definitions

An AE is defined as any undesirable effect experienced by a subject during a clinical study, whether or not considered related to the investigational product and/or the experimental treatment. All AEs reported spontaneously by the subject or observed by the investigator or his/her staff will be recorded. The assessment for AEs will begin with Visit 1 and end at the completion of Visit 6.

A serious adverse event (SAE) is any untoward medical occurrence or effect that at any dose:

- results in death;
- is life threatening (at the time of the event);
- requires hospitalization or prolongation of existing inpatient's hospitalization;
- results in persistent or significant disability or incapacity;
- is a congenital anomaly or birth defect;
- is a new event of the trial likely to affect the safety of the subjects, such as an unexpected outcome of an adverse reaction, lack of efficacy of an investigational product used for the treatment of a life threatening disease, major safety finding from a newly completed animal study, etc.

Note: The term "life-threatening" refers to an event in which the patient was at risk of death at the time of the event; it does not refer to an event which hypothetically might have caused death if it were more severe.

A suspected unexpected serious adverse reaction (SUSAR) is an SAE that is unlisted and associated with the use of the drug.

5.2 Evaluating AEs and SAEs

All AEs and SAEs will be evaluated in terms of the following items:

- Duration - start date and stop date
- Intensity - mild, moderate, or severe
- Causality - relationship between study medication and occurrence of each AE/SAE
- Outcome - resolved, resolved with sequelae, not resolved, unknown, death
- Action taken with study medication - none, discontinued, interrupted, unknown

5.3 Reporting of SAEs and Other Significant Events

Investigators are obliged to notify, by fax (or telephone), to the sponsor all serious adverse events and unexpected suspected adverse drug reactions IMMEDIATELY (within 24 hours of the investigator becoming aware of the event). The investigator will be requested to supply as much detailed information regarding the event that is available at the time of the initial contact. The investigator is also required to submit follow-up reports to the monitor until the adverse event has resolved or in the case of permanent impairment, until the adverse event stabilizes.

ANY SERIOUS ADVERSE EVENT WHETHER OR NOT RELATED TO THE STUDY DRUG MUST BE REPORTED IMMEDIATELY TO THE FOLLOWING PERSON:

ProductLife

Fax: +44 1223-413-689

E-mail: safety@verius.co.uk

For all SAEs a Serious Adverse Event Report Form that includes a detailed written description should be faxed to the above mentioned fax number, within 48 hours.

The responsible pharmacovigilance officer will immediately evaluate the SAEs for reporting to the appropriate regulatory agencies in consultation with the medical responsible person from the sponsor.

All serious, unexpected and related adverse events must also be reported to the reviewing IEC by the investigator. Confirmation that these reports have been submitted to the ethics committees must be forwarded to the monitor.

A Serious Adverse Event (SAE) is defined as any untoward medical occurrence that at any dose:

- results in death;
- is life-threatening (see Note);
- requires in-patient hospitalization or prolongation of existing hospitalization;
- results in persistent or significant disability/incapacity;
- is a congenital anomaly/birth defect.

Note: the term “life-threatening” refers to an event in which the patient was at risk of death at the time of the event; it does not refer to an event which hypothetically might have caused death if it were more severe.

Medical and scientific judgment should be exercised in deciding whether expedited reporting is appropriate in other situations, such as important medical events that may not be immediately life-threatening or result in death or hospitalization, but may jeopardize the patient or may require intervention to prevent one of the other outcomes listed in the definition above. These should also usually be considered serious. Also serious events which could be associated with the trial procedures (rather than with the IP) will be reported as SAEs, even if occurring outside the treatment procedure.

Under this protocol, the following event(s) will not be considered as (an) SAE(s) and should not be entered on the SAE form:

Pre-planned hospitalizations for diagnostic, therapeutic, or surgical procedures for a pre-existing condition that did not worsen during the course of a clinical trial. These pre-planned hospitalizations must be entered on the medical history form, including the condition requiring hospitalization. These events will, if applicable, still be reported as an AE.

Companion Diagnostic BV or its representatives will report all Suspected Unexpected Serious Adverse Reactions (SUSAR: SAEs that are unlisted and associated with the use of the drug) to the investigators, the Independent Ethics Committee (BEBO), the regulatory authorities (CCMO), and the CBG (College ter Beoordeling van Geneesmiddelen) within 24 hours.

The SUSAR report will contain the following information:

- the Eudract number of the study and the sponsors protocol study name and study number in which the SUSAR has taken place;
- the identification number which the investigator has given the SUSAR;
- the name and address of the person whom has reported the SUSAR;
- a description of why the SUSAR is suspect;
- a description of the study trial medication which suspectedly caused the SUSAR;
- the subjectcode, age and gender of the subject whom has had –or is having- the SUSAR;
- a description of the SUSAR, of it’s course/the course of events, and a description of the treatment thereof/ the action taken;
- an assessment of the (consequences of) the SUSAR by the investigator for the (ongoing) clinical study;
- any other relevant information regarding the SUSAR.

5.4 Other Contact Information

The study will be monitored by:

PSR Group B.V.
Planetenweg 5
2132 HN Hoofddorp

6 STATISTICAL METHODS

The described analyses in this Chapter will be fully described in the Statistical Analysis Plan (SAP CD001).

6.1 Determination of Sample Size

MedCalc software (MedCalc Statistics for Biomedical Research 12.7, Ostend, Belgium) was used for the ROC curve power analysis. Assuming balanced design of 75 cases in the positive group and 75 in the negative group, giving a total of 150 cases, resulted in 90% statistical power to be able to detect a minimum AUC of .650 from the null hypothesis value 0.5 (meaning no discriminating power) with an α-level of .05. The distribution of subjects among the groups was based on previous studies, in which the demarcation formula reliably allocated around 50% of all subjects (with complete screening data) to either group. In the Lybrido(s) Phase IIb studies (EB82 & EB90), 48% was marked as low sensitive and 52% as having high inhibition. This, combined with the theoretical background of the subdivision of these subjects (discussed earlier in the protocol), justifies these number in the power calculation.

The assumed AUC of .650 means that a randomly selected person from the positive group has a higher self-reported number of satisfying sexual events than that for a randomly selected person from the negative group 65% of the time. This assumption is considered realistic from an earlier study as stated above, where the correlation between the demarcation formula and another similar measurement of satisfaction was .83.

6.2 Analysis Populations

All analyses will be done using the intention-to-treat set and using a per-protocol set of subjects. The intention-to-treat set is comprised of all randomized subjects except those who have stopped or completed the study without taking any trial medication. The per-protocol set of subjects consists of all subjects for whom the minimum amount of data for valid analysis has been collected. Exact definitions for these 2 sets are given in the SAP CD001.

6.3 Study Endpoints Analyses

Study Endpoints Analyses

Firstly, we start to differentiate two subgroups on the basis of their scores on the demarcation formula into low and high sensitive to sexual cues, which could affect different responses on Lybrido and Lybridos. Secondly, we will calculate two variables on the basis of the difference between the number of satisfactory sexual events (SSE's) during both drug conditions and placebo: *Lybrido-effect* = number of SSEs during Lybrido minus number of SSEs during Placebo; *Lybridos-effect* = number of SSEs during Lybridos minus number of SSEs during Placebo.

Primary Endpoint Analyses

- Differences in Lybrido-effect and Lybridos-effect between groups will be analyzed with two-way mixed design ANOVA- with 2 within subjects effect variables (Lybrido-effect *versus*

Lybridos-effect) x 2 between subjects group variables (Low Sexual Attention versus High Sexual Attention. The dependent variables are the effect variables Lybrido-effect and Lybridos-effect. ;

- To evaluate the quality of performance of the present demarcation formula for the identification of the Lybrido and Lybridos respondents, a receiver operating characteristic (ROC) curves analysis will be performed. For the true status of the women a classification of women into low sexual attention versus high sexual attention will be made based on the Lybrido- and Lybridos-effect variables: when the score on the Lybrido-effect variable is higher than the score on the Lybridos-effect variable, a women is classified as having low sensitivity for sexual cues and vice versa. For the predicted status of the women the present demarcation formula is used. By using different cut-off values of the present demarcation formula, a ROC curve is constructed and the AUC is derived.

Testing of the new demarcation formula analysis

The same iterative process will be used as described in section 2.1.5.2. in the first half of the dataset (training set), to improve the original demarcation formula. This will be validated in the second half of the dataset (validation set or testing set) using the same analyses as in the validation of the original demarcation formula:

- Differences in Lybrido-effect and Lybridos-effect between groups will be analyzed with two-way mixed design ANOVAwith 2 within subjects effect variables (Lybrido-effect *versus* Lybridos-effect) x 2 between subjects group variables (Low Sexual Attention versus High Sexual Attention. The dependent variables are the effect variables Lybrido-effect and Lybridos-effect. ;

The subset of women with low sensitivity according to the old formula will be divided in women with low and high sensitivity according to the new formula. The following analysis will be performed only if the smallest group has N ≥ 15:

- Differences between groups will be analyzed with two-way repeated measures ANOVA: a 2 x (Drug: Placebo *versus* Lybrido) x 2 Group (Low sensitivity new versus high sensitivity new), in which “Drug” is the within-subject factor, and “Group” is the between-subject factor. The dependent variable is the change in number of SSEs between placebo and Lybrido.

The subset of women with high sensitivity according to the old formula will be divided in women with low and high sensitivity according to the new formula. The following analysis will be performed only if the smallest group has N ≥ 15:

- Differences between groups will be analyzed with two-way repeated measures ANOVA: a 2 x (Drug: Placebo *versus* Lybrido) x 2 Group (Low sensitivity new versus High sensitivity new),

in which "Drug" is the within-subject factor, and "Group" is the between-subject factor. The dependent variable is the change in number of SSEs between placebo and Lybrido.

- To evaluate the quality of performance of the renewed demarcation formula for the identification of the Lybrido and Lybridos respondents, a receiver operating characteristic (ROC) curves analysis will be performed. For the true status of the women a classification of women into low sexual attention versus high sexual attention will be made based on the Lybrido- and Lybridos-effect variables: when the score on the Lybrido-effect variable is higher than the score on the Lybridos-effect variable, a women is classified as having low sensitivity for sexual cues and vice versa. For the predicted status of the women the present demarcation formula is used. By using different cut-off values of the present demarcation formula, a ROC curve is constructed and the AUC is derived.

Exploratory analyses

Summary statistics will be described for the FSDS-R, PGI-I and PBE data.

The level of statistical significance will be set at $p < 0.05$, one-sided. Data will be presented as mean ± standard error of the mean. All statistical analyses will be conducted using IBM SPSS Statistics for Windows (Version 19.0. Armonk, NY: IBM Corp.).

7 STUDY ADMINISTRATION

7.1 Quality Assurance

The study will be audited to assess adherence to the Protocol and the Quality System of Emotional Brain BV. A Quality Assurance Officer appointed by Companion Diagnostic BV will perform the audit(s). The items to be audited will be defined in a Study Audit Plan for this study. An audit of the report will be included.

A certifying statement will be provided with the report outlining the types of audits, the dates of the audits performed and the dates the audits are reported to the coordinating investigator of Companion Diagnostic BV.

7.2 Monitoring

This study will be monitored by PSR Group BV in the Netherlands.

7.3 Archiving and Data Handling

Completed electronic CRFs (original) will be maintained by the study sites. After final database lock, the CRFs will be archived by the sponsor.

All documents and source data will be archived for at least 15 years according to the sponsor's procedures.

7.4 Drug Accountability

Drug supplies will be kept in a secure, limited access storage area under the recommended storage conditions. The principal investigator and/or co-investigators are responsible for monitoring the inventory of drug supplies. Drugs may not be used for any purpose other than as described in this protocol. The investigator or a responsible person designated by the investigator must carefully monitor and record the receipt, storage, and use of drug supplies. An accurate accounting of drug dispensed to each subject will be maintained, and these records will be made available to the sponsor or authorized representatives of the sponsor or authorities upon request. Additionally, frequency of on-demand drug intake will be monitored using the Medication Event Monitoring System (MEMS; MWV Switzerland Ltd, Sion, Switzerland). Every time a subject takes medication, this will be recorded electronically by MEMS.

The bottles containing the medication and the MEMSCap are not compatible, therefore designated bottles with identical labeling are being used during the study regimes. In order to fully adhere to regulations, these bottles are labeled in the controlled environment of ACE Pharmaceuticals B.V. , Zeewolde, The Netherlands. Regulations also require the patient to transfer the medication from the original bottle to the designated bottle with the MEMSCap, under supervision of a study physician. Drug stability will not be compromised by these actions, considering each study regime lasts only two weeks.

7.5 Ethical Considerations

7.5.1 General Regulations

The study will be performed in accordance with the latest versions of the Declaration of Helsinki and Good Clinical Practice guidelines.

7.5.2 Informed Consent

Written informed consent will be obtained before a subject participates in the study. The subject will be asked to read a subject information section of the consent form prospectively approved by the ethics committee and to sign it to indicate consent to participate in the study. Informed consent will be obtained before any screening visit assessments are performed.

8 DATA MANAGEMENT

8.1 General

All data management will be handled by PSR Group BV in the Netherlands. Electronic data capture will be employed.

8.2 e-Case Report Form

The investigator is responsible for the correct, complete, and timely documentation of all data generated and gathered during the trial. The data from the source document will be entered into the individual electronic CRF by the investigator designee.

Additional information, such as laboratory results, ECGs, and questionnaire responses will be considered original source documents. At the completion of each subject visit, the designee will complete data entry into the e-CRFs and the investigator or one of the sub-investigators will sign the appropriate section of the e-CRF to document his/her approval of the subject visit conclusion and confirm that all data entered are correct. Queries will be generated according to the data handling plan by automatic queries and manually added by the CRA and/ or data manager and resolved on an ongoing basis.

8.3 Data Transfer

8.3.1 Data for the Viedoc Clinical Database

PSR Group will prepare a data handling plan, which will include a description of current data handling procedures, quality control procedures, systems and software to be used, and data validation checks to be performed. This data handling plan will be sent to the responsible database programmer before the start of the trial. Additionally, the data handling plan, including the data validation plan (describing reference ranges and other data validation rules), will be reviewed and approved by the sponsor prior to initiation of the study.

8.3.2 Transfer of Data

Within 4 weeks after completion of the last study assessment and associated e-CRF, PSR Group will lock all e-CRF-based data and send the sponsor a signed statement confirming the database lock. Any changes made to these data after database lock must be reported by the responsible data manager and the reason for the change(s) documented.

The final study data will be transferred to the sponsor. The data will be formatted as SAS data sets and, if requested by the sponsor, in Excel or other format.

9 REPORTING, PUBLICATION, AND ARCHIVING

9.1 Reporting

PSR Group bv will provide Companion Diagnostics BV with a written report including all appendices in electronic format.

The final trial report of this clinical trial will be prepared by PSR Group bv according to the most recent version of PSR Group bv quality system. The released final report may be submitted to relevant health authorities to support a request for registration.

Except for compelling legal reasons, neither the sponsor nor the investigator will communicate to third parties any result of the clinical trial before they have both agreed on the results of the analysis and its interpretation.

9.2 Publication(s)

Companion Diagnostics BV recognizes the right of the investigator(s) to publish, but all publications must be based on data validated by Companion Diagnostics BV. Any such scientific paper, presentation, or other communication concerning the clinical trial described in this protocol will first be submitted to Companion Diagnostics BV, at least six weeks ahead of estimated publication or presentation, for written consent, which shall not be withheld unreasonably.

In order to protect its proprietary interests, Companion Diagnostics BV shall have the right to make its consent conditional upon proper representation of the interpretation of **both** Companion Diagnostics BV **and** the investigator(s) in the discussion of the data in such communications.

Companion Diagnostics BV is free to use the data for publication. The investigator(s) may be invited to be co-author(s). Companion Diagnostics BV will submit the manuscript to the investigator(s), who will have the right to be represented in such publications.

9.3 Archiving of Data

The investigator will arrange for the retention of the raw data from the institution for at least 15 years after the completion or discontinuation of the trial. Archiving will be done according to the quality system of the study center.

Data on SAEs must always be included in the Study Master File.

All data and documents must be made available if requested by relevant authorities. Records must be maintained to verify the existence of each subject in the clinical trial, and must contain the full name, last known address, telephone number, and other pertinent information of each subject.

10 INVESTIGATOR AGREEMENT

I have read this protocol and agree to conduct this clinical study as outlined herein. I will ensure that all sub-investigators and other study staff members have read and understand all aspects of this protocol. I agree to cooperate fully with Companion Diagnostics BV and its appointed Clinical Research Organization (CRO) during the study. I will adhere to all International Conference on Harmonization (ICH), revised Declaration of Helsinki (2008), and other applicable regulations and guidelines regarding clinical trials on a study drug during and after study completion.

Principal Investigator:

Printed Name: ______________________________

Signature: ______________________________

Date: ______________________________

Protocol CD001

Title: A double-blind, randomized, placebo-controlled, cross-over study to validate the predictive power of the demarcation formula for Lybrido and Lybridos efficacy in women with female sexual interest/arousal disorder, in the domestic situation

Version Number: 7, dated 11 April 2014

Date of Original Protocol: version 1, 01 May 2013

11 REFERENCES

American Psychiatric Association,2000. Diagnostic and Statistical Manual of Mental Disorders. American Psychatric Assocation, Washington.

Arias, B., Catalan, R., Gasto, C., Gutierrez, B., Fananas, L.,2005. Evidence for a combined genetic effect of the 5-HT(1A) receptor and serotonin transporter genes in the clinical outcome of major depressive patients treated with citalopram. J. Psychopharmacol. 19, 166-172.

Beauregard, M., Levesque, J., Bourgouin, P.,2001. Neural correlates of conscious self-regulation of emotion. J. Neurosci. 21, RC165.

Beevers, C.G., Ellis, A.J., Wells, T.T., McGeary, J.E.,2010. Serotonin transporter gene promoter region polymorphism and selective processing of emotional images. Biol. Psychol. 83, 260-265.

Berenbaum, S.A., Bryk, K.K., Nowak, N., Quigley, C.A., Moffat, S.,2009. Fingers as a marker of prenatal androgen exposure. Endocrinology. 150, 5119-5124.

Brand, J.S., van der Schouw, Y.T.,2010. Testosterone, SHBG and cardiovascular health in postmenopausal women. International journal of impotence research. 22, 91-104.

Breedlove, S.M., Cooke, B.M., Jordan, C.L.,1999. The orthodox view of brain sexual differentiation. Brain Behav. Evol. 54, 8-14.

Brown, W.M., Hines, M., Fane, B.A., Breedlove, S.M.,2002. Masculinized finger length patterns in human males and females with congenital adrenal hyperplasia. Horm. Behav. 42, 380-386.

Canli, T., Lesch, K.P.,2007. Long story short: the serotonin transporter in emotion regulation and social cognition. Nat. Neurosci. 10, 1103-1109.

Chambers, C.D., Garavan, H., Bellgrove, M.A.,2009. Insights into the neural basis of response inhibition from cognitive and clinical neuroscience. Neurosci. Biobehav. Rev. 33, 631-646.

Ciumas, C., Linden Hirschberg, A., Savic, I.,2009. High fetal testosterone and sexually dimorphic cerebral networks in females. Cereb. Cortex. 19, 1167-1174.

Cottingham, S.L., Pfaff, D.,1986. Interconnectedness of Steroid-Binding Hormones: Existence and Implications. Springer, Berlin, pp. 223-250.

Coviello, A.D., Haring, R., Wellons, M., Vaidya, D., Lehtimaki, T., Keildson, S., Lunetta, K.L., He, C., Fornage, M., Lagou, V., Mangino, M., Onland-Moret, N.C., Chen, B., Eriksson, J., Garcia, M., Liu, Y.M., Koster, A., Lohman, K., Lyytikainen, L.P., Petersen, A.K., Prescott, J., Stolk, L., Vandenput, L., Wood, A.R., Zhuang, W.V., Ruokonen, A., Hartikainen, A.L., Pouta, A., Bandinelli, S., Biffar, R., Brabant, G., Cox, D.G., Chen, Y., Cummings, S., Ferrucci, L., Gunter, M.J., Hankinson, S.E., Martikainen, H., Hofman, A., Homuth, G., Illig, T., Jansson, J.O., Johnson, A.D., Karasik, D., Karlsson, M., Kettunen, J., Kiel, D.P., Kraft, P., Liu, J., Ljunggren, O., Lorentzon, M., Maggio, M., Markus, M.R., Mellstrom, D., Miljkovic, I., Mirel, D., Nelson, S., Morin Papunen, L., Peeters, P.H., Prokopenko, I., Raffel, L., Reincke, M., Reiner, A.P., Rexrode, K., Rivadeneira, F., Schwartz, S.M., Siscovick, D., Soranzo, N., Stockl, D., Tworoger, S., Uitterlinden, A.G., van Gils, C.H., Vasan, R.S., Wichmann, H.E., Zhai, G., Bhasin, S., Bidlingmaier, M., Chanock, S.J., De Vivo, I., Harris, T.B., Hunter, D.J., Kahonen, M., Liu, S., Ouyang, P., Spector, T.D., van der Schouw, Y.T., Viikari, J., Wallaschofski, H., McCarthy, M.I., Frayling, T.M., Murray, A., Franks, S., Jarvelin, M.R., de Jong, F.H., Raitakari, O., Teumer, A., Ohlsson, C., Murabito, J.M., Perry, J.R.,2012. A genome-wide association meta-analysis of circulating sex

hormone-binding globulin reveals multiple Loci implicated in sex steroid hormone regulation. PLoS Genet. 8, e1002805.

Fernandez-Guasti, A., Picazo, O.,1997. Anxiolytic actions of diazepam, but not of buspirone, are influenced by gender and the endocrine stage. Behavioural brain research. 88, 213-8.

Freeman, L.M., Rissman, E.F.,1996. Neural aromatization and the control of sexual behavior. Trends Endocrinol Metab. 7, 334-8.

Goldstein, I., Lue, T.F., Padma-Nathan, H., Rosen, R.C., Steers, W.D., Wicker, P.A.,1998. Oral sildenafil in the treatment of erectile dysfunction. Sildenafil Study Group. N. Engl. J. Med. 338, 1397-1404.

Greep, R., Astwood, E.,1973. Handbook of Physiology, Section 7: Endocrinology : Female Reproductive System. II (in two parts), 603-613.

Hariri, A.R., Holmes, A.,2006. Genetics of emotional regulation: the role of the serotonin transporter in neural function. Trends Cogn. Sci. 10, 182-191.

Janssen, E., Bancroft, J.,2007. The dual control model: the role of sexual inhibition and excitation in sexual arousal and behaviour. In: Janssen, E. (Ed.), The Psychophysiology of Sex, Indiana University Press, Bloomington, pp. 197.

Joseph, R.,1999. Environmental influences on neural plasticity, the limbic system, emotional development and attachment: a review. Child Psychiatry Hum. Dev. 29, 189-208.

Karama, S., Lecours, A.R., Leroux, J.M., Bourgouin, P., Beaudoin, G., Joubert, S., Beauregard, M.,2002. Areas of brain activation in males and females during viewing of erotic film excerpts. Hum. Brain Mapp. 16, 1-13.

Knickmeyer, R.C., Wheelwright, S., Taylor, K., Raggatt, P., Hackett, G., Baron-Cohen, S.,2005. Gender-typed play and amniotic testosterone. Dev. Psychol. 41, 517-528.

Konishi, S., Jimura, K., Asari, T., Miyashita, Y.,2003. Transient activation of superior prefrontal cortex during inhibition of cognitive set. J. Neurosci. 23, 7776-7782.

Labrie, F., Luu-The, V., Belanger, A., Lin, S.X., Simard, J., Pelletier, G., Labrie, C.,2005. Is dehydroepiandrosterone a hormone? The Journal of endocrinology. 187, 169-96.

Le Francois, B., Czesak, M., Steubl, D., Albert, P.R.,2008. Transcriptional regulation at a HTR1A polymorphism associated with mental illness. Neuropharmacology. 55, 977-985.

Leslie, S.J., Atkins, G., Oliver, J.J., Webb, D.J.,2004. No adverse hemodynamic interaction between sildenafil and red wine. Clinical pharmacology and therapeutics. 76, 365-70.

Liddle, P.F., Kiehl, K.A., Smith, A.M.,2001. Event-related fMRI study of response inhibition. Hum. Brain Mapp. 12, 100-109.

Liu, Y.P., Wilkinson, L.S., Robbins, T.W.,2004. Effects of acute and chronic buspirone on impulsive choice and efflux of 5-HT and dopamine in hippocampus, nucleus accumbens and prefrontal cortex. Psychopharmacology (Berl). 173, 175-185.

Longcope, C.,1986. Adrenal and gonadal androgen secretion in normal females. Clinics in endocrinology and metabolism. 15, 213-28.

Lydiard, R.B.,2000. An overview of generalized anxiety disorder: disease state--appropriate therapy. Clinical therapeutics. 22 Suppl A, A3-19; discussion A20-4.

Manning, J.T., Scutt, D., Wilson, J., Lewis-Jones, D.I.,1998. The ratio of 2nd to 4th digit length: a predictor of sperm numbers and concentrations of testosterone, luteinizing hormone and oestrogen. Hum. Reprod. 13, 3000-3004.

Manson, J.E.,2008. Prenatal exposure to sex steroid hormones and behavioral/cognitive outcomes. Metabolism. 57 Suppl 2, S16-21.

Mekli, K., Payton, A., Miyajima, F., Platt, H., Thomas, E., Downey, D., Lloyd-Williams, K., Chase, D., Toth, Z.G., Elliott, R., Ollier, W.E., Anderson, I.M., Deakin, J.F., Bagdy, G., Juhasz, G.,2010. The HTR1A and HTR1B receptor genes influence stress-related information processing. Eur. Neuropsychopharmacol.

Milham, M.P., Banich, M.T., Barad, V.,2003. Competition for priority in processing increases prefrontal cortex's involvement in top-down control: an event-related fMRI study of the stroop task. Brain Res. Cogn. Brain Res. 17, 212-222.

Murphy, D.L., Fox, M.A., Timpano, K.R., Moya, P.R., Ren-Patterson, R., Andrews, A.M., Holmes, A., Lesch, K.P., Wendland, J.R.,2008. How the serotonin story is being rewritten by new gene-based discoveries principally related to SLC6A4, the serotonin transporter gene, which functions to influence all cellular serotonin systems. Neuropharmacology. 55, 932-960.

Okten, A., Kalyoncu, M., Yaris, N.,2002. The ratio of second- and fourth-digit lengths and congenital adrenal hyperplasia due to 21-hydroxylase deficiency. Early Hum. Dev. 70, 47-54.

Perez-Edgar, K., Bar-Haim, Y., McDermott, J.M., Gorodetsky, E., Hodgkinson, C.A., Goldman, D., Ernst, M., Pine, D.S., Fox, N.A.,2010. Variations in the serotonin-transporter gene are associated with attention bias patterns to positive and negative emotion faces. Biol. Psychol. 83, 269-271.

Poels, S., Bloemers, J., van Rooij, K., Goldstein, I., Gerritsen, J., van Ham, D., van Mameren, F., Chivers, M., Everaerd, W., Koppeschaar, H., Olivier, B., Tuiten, A.,2013. Toward personalized sexual medicine (part 2): testosterone combined with a PDE5 inhibitor increases sexual satisfaction in women with HSDD and FSAD, and a low sensitive system for sexual cues. J. Sex. Med. 10, 810-823.

Quigley, C.A., De Bellis, A., Marschke, K.B., el-Awady, M.K., Wilson, E.M., French, F.S.,1995. Androgen receptor defects: historical, clinical, and molecular perspectives. Endocr. Rev. 16, 271-321.

Rubia, K., Russell, T., Overmeyer, S., Brammer, M.J., Bullmore, E.T., Sharma, T., Simmons, A., Williams, S.C., Giampietro, V., Andrew, C.M., Taylor, E.,2001. Mapping motor inhibition: conjunctive brain activations across different versions of go/no-go and stop tasks. Neuroimage. 13, 250-261.

Schuckit, M.A.,1987. Alcohol and drug interactions with antianxiety medications. The American journal of medicine. 82, 27-33.

Sherwin, B.B., Gelfand, M.M., Brender, W.,1985. Androgen enhances sexual motivation in females: a prospective, crossover study of sex steroid administration in the surgical menopause. Psychosomatic medicine. 47, 339-51.

Stoleru, S., Redoute, J., Costes, N., Lavenne, F., Bars, D.L., Dechaud, H., Forest, M.G., Pugeat, M., Cinotti, L., Pujol, J.F.,2003. Brain processing of visual sexual stimuli in men with hypoactive sexual desire disorder. Psychiatry Res. 124, 67-86.

Stoleru, S., Gregoire, M.C., Gerard, D., Decety, J., Lafarge, E., Cinotti, L., Lavenne, F., Le Bars, D., Vernet-Maury, E., Rada, H., Collet, C., Mazoyer, B., Forest, M.G., Magnin, F., Spira, A., Comar, D.,1999. Neuroanatomical correlates of visually evoked sexual arousal in human males. Arch. Sex. Behav. 28, 1-21.

Tuiten, A., Van Honk, J., Koppeschaar, H., Bernaards, C., Thijssen, J., Verbaten, R.,2000. Time course of effects of testosterone administration on sexual arousal in women. Arch Gen Psychiatry. 57, 149-53; discussion 155-6.

Tuiten, A., van Honk, J., Verbaten, R., Laan, E., Everaerd, W., Stam, H.,2002. Can sublingual testosterone increase subjective and physiological measures of laboratory-induced sexual arousal? Archives of general psychiatry. 59, 465-6.

Van der Made, F., Bloemers, J., Van Ham, D., El Yassem, W., Kleiverda, G., Everaerd, W., Olivier, B., Tuiten, A.,2009. Childhood sexual abuse, selective attention for sexual cues and the effects of testosterone with or without vardenafil on physiological sexual arousal in women with sexual dysfunction: A pilot study. J Sex Med. 6, 429-439; 429.

van der Made, F., Bloemers, J., Yassem, W.E., Kleiverda, G., Everaerd, W., van Ham, D., Olivier, B., Koppeschaar, H., Tuiten, A.,2009. The influence of testosterone combined with a PDE5-inhibitor on cognitive, affective, and physiological sexual functioning in women suffering from sexual dysfunction. The journal of sexual medicine. 6, 777-90.

van Rooij, K., Poels, S., Bloemers, J., Goldstein, I., Gerritsen, J., van Ham, D., van Mameren, F., Chivers, M., Everaerd, W., Koppeschaar, H., Olivier, B., Tuiten, A.,2013. Toward personalized sexual medicine (part 3): testosterone combined with a Serotonin1A receptor agonist increases sexual satisfaction in women with HSDD and FSAD, and dysfunctional activation of sexual inhibitory mechanisms. J. Sex. Med. 10, 824-837.

Vanbillemont, G., Bogaert, V., De Bacquer, D., Lapauw, B., Goemaere, S., Toye, K., Van Steen, K., Taes, Y., Kaufman, J.M.,2009. Polymorphisms of the SHBG gene contribute to the interindividual

variation of sex steroid hormone blood levels in young, middle-aged and elderly men. Clin. Endocrinol. (Oxf). 70, 303-310.

Varga, G., Szekely, A., Antal, P., Sarkozy, P., Nemoda, Z., Demetrovics, Z., Sasvari-Szekely, M.,2012. Additive effects of serotonergic and dopaminergic polymorphisms on trait impulsivity. Am. J. Med. Genet. B. Neuropsychiatr. Genet. 159B, 281-288.

Waxenberg, S.E., Drellich, M.G., Sutherland, A.M.,1959. The role of hormones in human behavior. I. Changes in female sexuality after adrenalectomy. The Journal of clinical endocrinology and metabolism. 19, 193-202.

Xita, N., Tsatsoulis, A.,2010. Genetic variants of sex hormone-binding globulin and their biological consequences. Mol. Cell. Endocrinol. 316, 60-65.

Xita, N., Georgiou, I., Lazaros, L., Psofaki, V., Kolios, G., Tsatsoulis, A.,2008. The role of sex hormone-binding globulin and androgen receptor gene variants in the development of polycystic ovary syndrome. Hum. Reprod. 23, 693-698.

Zhang, K., Xu, Q., Xu, Y., Yang, H., Luo, J., Sun, Y., Sun, N., Wang, S., Shen, Y.,2009. The combined effects of the 5-HTTLPR and 5-HTR1A genes modulates the relationship between negative life events and major depressive disorder in a Chinese population. J. Affect. Disord. 114, 224-231.

Zitzmann, M.,2009. Pharmacogenetics of testosterone replacement therapy. Pharmacogenomics. 10, 1341-1349.

12 APPENDICES

Appendix 1: Study Timeline

Appendix 2: Schedule of Study Procedures

Appendix 3: Schedule of Hematology and Blood Chemistry Assessments

Appendix 4: Sexual Anamnesis Questionnaire-Diagnostic (SAQ-D)

Appendix 5: Weekly Diary

Appendix 6: Sexual Event Diary (SED)

Appendix 7: Sexual Motivation Questionnaire (SMQ)

Appendix 8: Female Sexual Distress Scale Revised (FSDS-R)

Appendix 9 : Patient Benefit Evaluation (PBE)

Appendix 10 : Patient's Global Impression of Improvement (PGI-I)

APPENDIX 1: Study Timeline

Week	Visit	Period
−4*	V0 Screening	Enrollment
0	V1 Start Up Visit	Placebo run-in
1		Placebo run-in
2	V2 Study regimen follow-up	Treatment period 1
3		Treatment period 1
4	V3 Study regimen follow-up	Treatment period 2
5		Treatment period 2
6	V4 Study regimen follow-up	Treatment period 3
7		Treatment period 3
8	V5 Study regimen follow-up	
9		
11	V6 Final follow-up	

*Average, maximum 12 weeks (-12)

APPENDIX 2: Schedule of study procedures

Procedure	V0	V1	V2	V3	V4	V5	V6	Home
Informed consent	X							
Inclusion/exclusion criteria	X	X	X	X	X	X	X	
Randomization		X[a]						
Demographics	X							
Sexual anamnesis	X							
Improvement interview psychologist/sexologist	X		X	X	X	X	X	
Psychoeducational DVD		X						
Medical history	X							
Physical examination	X						X	
Vital signs	X	X	X	X	X	X	X	
Hyperandrogenism monitoring	X	X	X	X	X	X	X	
Height and body weight [b]	X						X[b]	
Pregnancy test (urine)	X	X	X	X	X	X	X	
Electrocardiogram	X							
2D:4D measurement, left hand		X						
Urine drug screen	X							
Urinalysis	X							
Hematology assessment	X						X	
Blood chemistry assessment	X						X	
Biomarker assessment		X						
Study medication dispensing		X	X	X	X			
Study medication interim count			X	X	X	X	X	
Concomitant medications	X	X	X	X	X	X	X	
AE and SAE monitoring		X	X	X	X	X	X	
Questionnaire: SAQ–D	X							
Questionnaire: SMQ	X							
Questionnaire: FSDS-R	X	X	X	X	X	X	X	
Questionnaire: PGI-I & PBE			X	X	X	X		

Questionnaire: SED								X
Questionnaire: weekly diary								X

[a] Randomization will occur before V1.

[b] Only body weight will be determined at Visit 6.

APPENDIX 3: Schedule of Hematology and Blood Chemistry Assessments

	Visit		
**Tests**	**V0**	**V1**	**V6**
Creatinine	X		X
Albumin	X		
GGT	X		X
ALT	X		X
AST	X		X
Thyroid stimulating hormone	X		
Free T4	X		
Hemoglobin	X		X
Hematocrit	X		X
Erythrocytes	X		X
Indices	X		X
Leucocytes	X		X
Thrombocytes	X		X
Hemoglobin A1c	X		X
Glucose	X		X
HbA1c	X		X
LH	X		
FSH	X		
Prolactin	X		
Estradiol	X		
SHBG	X		X
Testosterone	X		X
HBsAg	X		
Anti-HCV/hepatitis C	X		
Anti-HIV 1 and 2	X		

	Visit		
**Tests (continued)**	**V0**	**V1**	**V6**
5-HT1A polymorphism		X	
CAG-AR		X	
ADT-G		X	
3α-diol-G		X	
Micorarray chip		X	

Notes: ALT = alanine aminotransferase; AST = aspartate aminotransferase; FSH = follicle stimulating hormone; GGT = gamma glutamyl transpeptidase; HBsAg = hepatitis B surface antigen; HCV = hepatitis C virus; HIV = human immunodeficiency virus; LH = luteininzing hormone; SHBG = sex hormone binding globulin; T4 = thyroxine; 5-HT1a polymorphism= polymorphism in serotonin 1A receptor; CAG-AR= cytosine adenine guanine repeat androgen receptor; ADT-G = androsterone glucuronide; 3α-diol-G = 3-alpha-androstanediol glucuronide; for a detailed overview of the genetic markers determined by microarray chips see the section Definition of terms.
