# Supplementary material for: A survival of the fittest strategy for the selection of genotypes by which drug responders and non-responders can be predicted in small groups
Source: PLoS One. 2021 Mar 5;16(3):e0246828. doi: 10.1371/journal.pone.0246828 (PMC7935233; doi:10.1371/journal.pone.0246828)
Supplement: S2 File — (DOCX) [file pone.0246828.s003.docx]

**A Survival of the Fittest Strategy for the Selection of Genotypes by which Drug Responders and Non-Responders can be Predicted in small groups**

**Supplementary Methods**

**Medication**

**Used Treatments and its Rationality**

As described in the introduction, for one subgroup of patients it was assumed that brain systems for sexual excitation were relatively insensitive, resulting in low sexual desire/arousal. In another subgroup, we assumed that sexual stimulation increases the activity of inhibitory sexual mechanisms that results in comparable sexual complaints of low desire. The presumed neurobiological mechanism for this inhibitory effect is a phasic increase in serotonergic activity, provoked by sexual stimulation, in the left dorsolateral prefrontal cortex[1]. Basic to both drug treatments is the delayed effect of a testosterone (T) pulse (induced by 0.5 mg sublingual T) on physiological and subjective sexual responses established in sexually functional women, which occurs 3 to 6 hours after the plasma T peak induction[2,3].

An increase in sexual motivation/desire is a necessary condition for the effectiveness of phosphodiesterase type 5 (PDE5) inhibitors in both men and women. For low sensitive women, a fixed-dose/fixed-release combination tablet with sublingual T combined with the PDE-5 inhibitor sildenafil (S) has been designed, in which the time of the peak plasma concentration of S coincides with behavioral effects of T after the 3 to 6 hours delay[4] (Treatment: T+S). The treatment developed for women suffering from high sexual inhibition is a tablet with sublingual T, combined with the 5-hydroxytryptamine 1A (5-HT_1A_) receptor agonist buspirone (B), which is released in such a timeframe that the pharmacological effects of B coincides with the sexual motivational window induced by T administration[5] (Treatment: T+B). Acute treatment with 5HT_1A_ receptor agonists decreases the release of 5-HT[6], and subsequent serotonergic firing activity[7,8], which then transiently decreases extracellular concentrations of serotonin, thus dampening the phasic serotonergic inhibitory control in the prefrontal cortex (PFC). For both drugs the delayed release is achieved by a pH-independent delayed release coating and is necessary to accomplish overlap of the pharmacodynamic effects of the two active pharmaceutical ingredients (see paragraph medication, dosing and instructions for further details).

**Description of Investigational Product**

**Sublingual testosterone + sildenafil (T+S)**: a dual-route/dual-release fixed-dose combination of testosterone and sildenafil citrate[4]. The drug product is a 9 mm, round, biconvex, white, menthol flavored tablet for sublingual administration. The outer, polymeric film coating contains testosterone (0.5 mg) that is released immediately after sublingual administration. The inner core of the tablet contains sildenafil (50 mg). This inner core has a polymeric coating designed to delay release of the sildenafil for approximately 2.5 hours; after this time delay, the sildenafil is released immediately (i.e., it is not a sustained release).

**Sublingual testosterone + buspirone (T+B)**: a dual-route/dual-release fixed-dose combination of testosterone and buspirone hydrochloride[5]. The drug product is a 9 mm, round, biconvex, white, menthol flavored tablet for sublingual and oral administration. The appearance, method of administration and flavor or T+B is identical to T+S. The outer, polymeric, film coating contains testosterone (0.5 mg) that is released immediately after sublingual administration. The inner core of the tablet contains buspirone hydrochloride (10 mg). This inner core has a polymeric coating designed to delay release of the buspirone for approximately 2.5 hours; after this time delay, the buspirone is released immediately (i.e., it is not a sustained release).

**Placebo**: Placebo tablets had the exact same appearance and flavor as the fixed-dose combination T+S and T+B tablets containing active pharmaceutical ingredients. All medication was manufactured and packaged at Piramal Healthcare UK (Morpeth, UK).

**Supplementary Results**

The 69 SNPs that were used for the T+S formula and the 65 that were used for the T+B formula are shown in **Supplementary** **Table 1** below. Of these SNPs, 15 were used in both formulae.

**Supplementary Table 1** Selected SNPs of the two companion demarcation tools

|  |  | T+S | | | T+B | | |
| --- | --- | --- | --- | --- | --- | --- | --- |
| SNP | Gene | Allele  -1/count | Allele 0/count | Allele 1/count | Allele  -1/count | Allele 0/count | Allele 1/count |
| rs3761422 | ADORA2A |  |  |  | **AA/22** | GG/47 | AG/55 |
| rs1079078 | ADRA1A | CC/6 | AA/74 | **AC/44** |  |  |  |
| rs2036109 | ADRA1A | CC/59 | AC/50 | **AA/13** |  |  |  |
| rs472151 | ADRA1A | **GG/44** | AA/22 | AG/55 | GG/44 | AA/22 | **AG/55** |
| rs556793 | ADRA1A |  |  |  | AA/41 | AG/69 | **GG/13** |
| rs573514 | ADRA1A | **AG/65** | AA/36 | GG/24 | AG/65 | AA/36 | **GG/24** |
| rs577366 | ADRA1A | GG/10 | AA/58 | **AG/57** |  |  |  |
| rs7718362 | ADRA1B |  |  |  | AA/116 |  | **AC/10** |
| rs2097628 | DBH | AG/57 | GG/21 | **AA/46** |  |  |  |
| rs4436578 | DRD2 | AA/95 |  | **AG/32** |  |  |  |
| rs4648317 | DRD2 |  |  |  | **GG/93** | AG/32 |  |
| rs16822416 | DRD3 | **AG/12** |  | GG/115 |  |  |  |
| rs3773678 | DRD3 | **GG/106** |  | AG/21 |  |  |  |
| rs963468 | DRD3 | **AG/56** | GG/42 | AA/28 | **AG/56** | GG/42 | AA/28 |
| rs1285057 | ESR1 | **GG/50** | AA/21 | AG/55 |  |  |  |
| rs1890010 | ESR1 |  |  |  | **AG/49** | AA/65 | GG/10 |
| rs1999805 | ESR1 |  |  |  | **AA/42** | GG/22 | AG/62 |
| rs2347869 | ESR1 | **AA/48** | AC/54 | CC/17 |  |  |  |
| rs2813544 | ESR1 | AA/85 | AG/35 | **GG/6** |  |  |  |
| rs2881766 | ESR1 |  |  |  | AC/40 | AA/80 | **CC/3** |
| rs3020333 | ESR1 | AA/30 | AG/66 | **GG/27** |  |  |  |
| rs3020365 | ESR1 |  |  |  | **AA/8** | AC/57 | CC/54 |
| rs3020418 | ESR1 |  |  |  | AA/10 | AG/50 | **GG/66** |
| rs3778082 | ESR1 |  |  |  | **AG/28** | GG/96 | AA/3 |
| rs4583998 | ESR1 | **AA/12** | GG/52 | AG/62 |  |  |  |
| rs6902771 | ESR1 | AG/69 | GG/38 | **AA/17** | **AG/69** | AA/17 | GG/38 |
| rs6908732 | ESR1 |  |  |  | GG/4 | AG/40 | **AA/79** |
| rs6912184 | ESR1 |  |  |  | GG/10 | AG/47 | **AA/57** |
| rs712219 | ESR1 | **GG/45** | AA/17 | AG/60 | GG/45 | AA/17 | **AG/60** |
| rs726281 | ESR1 | **AA/69** | GG/6 | AG/51 |  |  |  |
| rs7450824 | ESR1 | GG/5 | AG/38 | **AA/80** |  |  |  |
| rs851982 | ESR1 |  |  |  | **GG/19** | AG/59 | AA/44 |
| rs851995 | ESR1 | **AA/24** | AG/57 | GG/28 |  |  |  |
| rs9479087 | ESR1 | **AA/3** | AG/40 | GG/84 |  |  |  |
| rs985191 | ESR1 | **CC/2** | AA/102 | AC/23 |  |  |  |
| rs1256046 | ESR2 | **CC/42** | AC/61 | AA/16 |  |  |  |
| rs1256114 | ESR2 |  |  |  | **GG/87** | AG/34 | AA/4 |
| rs17766755 | ESR2 | **AA/7** | AG/66 | GG/41 | **AA/7** | AG/66 | GG/41 |
| rs1887994 | ESR2 | AC/23 |  | **CC/103** |  |  |  |
| rs4365213 | ESR2 | GG/28 | AG/61 | **AA/36** |  |  |  |
| rs10806098 | HTR1B |  |  |  | AA/8 | GG/61 | **AG/55** |
| rs9361235 | HTR1B |  |  |  | AG/61 | AA/13 | **GG/41** |
| rs6936751 | HTR1E | **AA/111** |  | AG/15 |  |  |  |
| rs7751022 | HTR1E |  |  |  | AG/9 |  | **GG/118** |
| rs828361 | HTR1E |  |  |  | AA/3 | GG/86 | **AG/38** |
| rs9344662 | HTR1E | **GG/99** | AA/4 | AG/24 |  |  |  |
| rs12714696 | HTR1F | AG/53 | AA/18 | **GG/44** |  |  |  |
| rs1431153 | HTR1F | **AG/53** | GG/20 | AA/49 |  |  |  |
| rs6770013 | HTR1F | AA/22 | AC/52 | **CC/51** |  |  |  |
| rs7652406 | HTR1F | AG/52 | AA/19 | **GG/51** |  |  |  |
| rs9681975 | HTR1F | **AG/54** | AA/17 | GG/55 |  |  |  |
| rs9863076 | HTR1F | AC/53 | AA/22 | **CC/52** |  |  |  |
| rs2770296 | HTR2A |  |  |  | **AG/50** | AA/66 | GG/5 |
| rs4942587 | HTR2A |  |  |  | **AG/50** | AA/70 | GG/6 |
| rs582385 | HTR2A | **AA/87** | GG/5 | AG/33 |  |  |  |
| rs731779 | HTR2A |  |  |  | **AC/40** | AA/83 | CC/2 |
| rs9567737 | HTR2A |  |  |  | **AG/66** | AA/28 | GG/26 |
| rs9567746 | HTR2A |  |  |  | **AG/40** | AA/82 | GG/3 |
| rs1549339 | HTR2B | **GG/51** | AA/18 | AG/53 |  |  |  |
| rs17619636 | HTR2B |  |  |  | **AC/9** |  | AA/118 |
| rs7581418 | HTR2B | **GG/49** | AA/17 | AG/53 |  |  |  |
| rs1360851 | HTR2C | AA/80 | CC/5 | **AC/39** |  |  |  |
| rs1414324 | HTR2C | GG/80 | AA/5 | **AG/40** | AA/5 | GG/80 | **AG/40** |
| rs1801412 | HTR2C | **AC/12** |  | AA/115 |  |  |  |
| rs2428717 | HTR2C | GG/82 | AA/6 | **AG/39** |  |  |  |
| rs2497508 | HTR2C | AA/84 | GG/6 | **AG/37** |  |  |  |
| rs2497524 | HTR2C | AA/83 | CC/6 | **AC/38** |  |  |  |
| rs2497541 | HTR2C | AA/80 | GG/5 | **AG/38** |  |  |  |
| rs4332303 | HTR2C | GG/82 | AA/5 | **AG/39** |  |  |  |
| rs4911871 | HTR2C | AG/39 |  | **AA/86** | GG/2 | AG/39 | **AA/86** |
| rs556677 | HTR2C | GG/79 | AA/6 | **AG/41** | AA/6 | GG/79 | **AG/41** |
| rs10160548 | HTR3A |  |  |  | **AC/55** | AA/54 | CC/13 |
| rs6792482 | HTR3D |  |  |  | GG/23 | AG/66 | **AA/25** |
| rs10040819 | HTR4 |  |  |  | **AC/29** | CC/94 | AA/1 |
| rs10078551 | HTR4 |  |  |  | AG/56 | AA/47 | **GG/20** |
| rs12152801 | HTR4 | CC/20 | AA/49 | **AC/54** |  |  |  |
| rs13182913 | HTR4 |  |  |  | AG/54 | AA/49 | **GG/19** |
| rs1345697 | HTR4 |  |  |  | **AG/63** | AA/23 | GG/37 |
| rs17639735 | HTR4 |  |  |  | GG/4 | AG/18 | **AA/102** |
| rs3995091 | HTR4 |  |  |  | GG/46 | AG/51 | **AA/19** |
| rs10785973 | HTR7 | CC/57 | AC/55 | **AA/4** |  |  |  |
| rs10881838 | HTR7 |  |  |  | **GG/6** | AG/54 | AA/67 |
| rs11186309 | HTR7 |  |  |  | **GG/5** | AA/74 | AG/44 |
| rs11186320 | HTR7 |  |  |  | **GG/6** | AG/53 | AA/68 |
| rs11527868 | HTR7 |  |  |  | AG/11 |  | **GG/110** |
| rs2465115 | HTR7 | AC/40 | CC/10 | **AA/77** |  |  |  |
| rs3740046 | HTR7 | AG/40 | AA/6 | **GG/76** |  |  |  |
| rs10521432 | MAOB | **GG/65** | AG/58 | AA/4 | GG/65 | AG/58 | **AA/4** |
| rs3027452 | MAOB |  |  |  | AA/2 | GG/85 | **AG/40** |
| rs1353939 | NOS1 |  |  |  | **GG/84** | AG/40 | AA/3 |
| rs2291908 | NOS1 |  |  |  | AA/65 | AG/54 | **GG/7** |
| rs570234 | NOS1 | CC/17 | AC/62 | **AA/38** |  |  |  |
| rs7295972 | NOS1 | GG/26 | AG/63 | **AA/22** |  |  |  |
| rs816353 | NOS1 | CC/49 | AC/58 | **AA/11** | CC/49 | AC/58 | **AA/11** |
| rs904658 | NOS1 | **AA/63** | AC/55 | CC/8 | AA/63 | AC/55 | **CC/8** |
| rs10482672 | NR3C1 |  |  |  | **GG/98** | AG/27 | AA/2 |
| rs4912910 | NR3C1 |  |  |  | **AG/48** | GG/61 | AA/14 |
| rs6084253 | OXT | AG/30 | AA/4 | **GG/93** |  |  |  |
| rs11131149 | OXTR | GG/48 | AA/7 | **AG/54** | AG/54 | GG/48 | **AA/7** |
| rs2268495 | OXTR | AA/1 | AG/44 | **GG/77** |  |  |  |
| rs12646525 | PDE5A |  |  |  | **GG/80** | AG/40 | AA/4 |
| rs17358524 | PDE5A | **AG/28** |  | AA/94 | **AA/94** | AG/28 | GG/2 |
| rs1205960 | PRL | AG/58 | GG/62 | **AA/7** |  |  |  |
| rs13354826 | PRLR | GG/23 | AG/54 | **AA/46** |  |  |  |
| rs37364 | PRLR |  |  |  | **AA/76** | AC/44 | CC/3 |
| rs37383 | PRLR |  |  |  | **GG/4** | AA/94 | AG/27 |
| rs4703509 | PRLR |  |  |  | GG/7 | AA/71 | **AG/48** |
| rs7731153 | PRLR | **AA/107** |  | AG/20 |  |  |  |
| rs7734558 | PRLR | AG/56 | AA/42 | **GG/25** |  |  |  |
| rs7735260 | PRLR | **AG/20** |  | GG/103 |  |  |  |
| rs15534 | SLC6A2NET |  |  |  | **AG/35** |  | GG/90 |
| rs36030 | SLC6A2NET | GG/4 | AA/87 | **AG/36** | GG/4 | AA/87 | **AG/36** |
| rs3785157 | SLC6A2NET | **AG/58** | GG/49 | AA/12 |  |  |  |
| rs41154 | SLC6A2NET |  |  |  | GG/19 | AG/60 | **AA/48** |
| rs3776512 | SLC6A3DAT |  |  |  | AA/1 | AG/49 | **GG/76** |
| rs460000 | SLC6A3DAT | AC/36 | CC/86 | **AA/3** |  |  |  |
| rs140701 | SLC6A45HTT |  |  |  | AG/72 | AA/17 | **GG/35** |
| rs2066713 | SLC6A45HTT | **GG/34** | AG/71 | AA/9 |  |  |  |
| rs4429345 | SLC6A45HTT |  |  |  | AG/64 | GG/28 | **AA/33** |

Notes: List of selected SNPs, with associated genes, per drug treatment. Per treatment three columns are displayed: Allele -1 is the negative risk allele which is colored red if selected in the final formula, whereas the positive risk allele (valued 1) is colored green. Allele number 0 is never used and only displayed for completeness. The ordering of the alleles, to risk alleles, may differ between treatments - as they are separately ordered to their respective primary endpoints. SNPs that were not used for said formula are left blank. SNPs used neither in T+S nor T+B are excluded from this overview.

**References**

[1] Pfaus JG. Pathways of sexual desire. J Sex Med 2009;6:1506–33. doi:10.1111/j.1743-6109.2009.01309.x.

[2] Tuiten A, Van Honk J, Koppeschaar H, Bernaards C, Thijssen J, Verbaten R. Time course of effects of testosterone administration on sexual arousal in women. Arch Gen Psychiatry 2000;57:149–53; discussion 155-6.

[3] Tuiten A, van Honk J, Verbaten R, Laan E, Everaerd W, Stam H. Can sublingual testosterone increase subjective and physiological measures of laboratory-induced sexual arousal? Arch Gen Psychiatry 2002;59:465–6.

[4] Bloemers J, van Rooij K, de Leede L, Frijlink HW, Koppeschaar HPF, Olivier B, et al. Single dose sublingual testosterone and oral sildenafil versus a dual-route/dual-release fixed-dose combination tablet: a pharmacokinetic comparison: Pharmacokinetics of testosterone and sildenafil combination tablet. Br J Clin Pharmacol 2016:n/a-n/a. doi:10.1111/bcp.12887.

[5] van Rooij K, de Leede L, Frijlink HW, Bloemers J, Poels S, Koppeschaar H, et al. Pharmacokinetics of a prototype formulation of sublingual testosterone and a buspirone tablet, versus an advanced combination tablet of testosterone and buspirone in healthy premenopausal women. Drugs RD 2014;14:125–32. doi:10.1007/s40268-014-0047-7.

[6] Liu YP, Wilkinson LS, Robbins TW. Effects of acute and chronic buspirone on impulsive choice and efflux of 5-HT and dopamine in hippocampus, nucleus accumbens and prefrontal cortex. Psychopharmacology (Berl) 2004;173:175–85. doi:10.1007/s00213-003-1726-1.

[7] Sprouse JS, Aghajanian GK. Electrophysiological responses of serotoninergic dorsal raphe neurons to 5-HT1A and 5-HT1B agonists. Synap N Y N 1987;1:3–9. doi:10.1002/syn.890010103.

[8] Hamon M, Fattaccini CM, Adrien J, Gallissot MC, Martin P, Gozlan H. Alterations of central serotonin and dopamine turnover in rats treated with ipsapirone and other 5-hydroxytryptamine1A agonists with potential anxiolytic properties. J Pharmacol Exp Ther 1988;246:745–52.
