# Supplementary material for: A survival of the fittest strategy for the selection of genotypes by which drug responders and non-responders can be predicted in small groups
Source: PLoS One. 2021 Mar 5;16(3):e0246828. doi: 10.1371/journal.pone.0246828 (PMC7935233; doi:10.1371/journal.pone.0246828)
Supplement: S1 Data — (ZIP) [file pone.0246828.s004.zip › README.docx]

**Data manipulation**

In this folder you can find the data we used for the publication of Höhle et al: “A survival of the fittest strategy for the selection of genotypes by which drug responders and non-responders can be predicted in small groups”.

The primary SNP data was sequenced by Eurofins Aros (Denmark). This we received in 'txt' files, one for each participant. (note 1)

In the sub-folder "SNPs raw" you find the original data. This is a subset - as discussed in the manuscript - of the original ~300.000 SNPs. These files can be requested by the author.

From these ~300.000 we filtered SNPs as discussed in the manuscript, resulting in 285 SNPs.

From "SNPs raw" the nucleotides were recoded to numbers - at this stage the numbers have no mathematical value.

Next the genotypes were recoded to effect for the different measures - see folder "coding_sse".

In subfolder 'xlsx' you find two files, each for another target variable, in which a variable is set called "random_X". This variable is used to decide which cases will be used for the recoding of the genotype score towards the target variable.

The recoding is done using a proprietary piece of software (writing in Java) by the corresponding author.

Input files for the program were *_der.txt and *_val.txt. The former was used for the recoding of the numerical genotypes to the, so-called, A-variables (genotypes recoded to the effect of their respective target variables).

The recoding to the new values are described in "allele_score_mapping_file_*". This file is then used, also, for the recoding of the validation file.

The program's result are in A_lybrido*.txt, der -> derivatie and val -> validatie. The remaining file is for display purposes only and describes the exact effect of the different genotypes to the target variable of the derivation set.

For technical reasons the combination of the derivation and validation sets, for each of the target variables, can be seen in the folder "xlsx - resultaat". In these files, one finds the first and the recoded values, the latter prefixed with A_, of each of the SNPs.

These aforementioned A-variables were then used as input for the SPSS analyses.

See PP.CD_Lybrido_Dichotomie_28-03-2019.sav and PP.CD_Lybridos_28_03-2019.sav for the subsequent analysis.

Subfolder ‘ROC analyses and figures’ contains the derived .sav and .sps files with which the ROC analyses were performed.

**Note 1**: There is more to that story. There are multiple types of sequencer, one specifically for women. Since our research is focussed on women, we wanted that one.

However, the results of the first sequenced set included SNPs on the Y chromosome - which is unusual for women. Thus we let them redo the scan. We did a per SNP comparison between those runs, and the SNPs that were sequenced in both, and had no missing values, were identical - mostly. For some there was a third rerun. We combined the results from the different runs to fill in the missing values.

There have been a few inconsistencies in the sequencing files, as can be seen in "mismatches.txt". There are nine genotypes for which the DNA sequencer displayed two different genotypes. For four of these we could recover the value from another DNA sequencer we did – GSGT, HumanOmniExpress. These four were consistent with the most recent DNA analyses. For all of the nine genotypes with inconsistencies we used to results of the latest sequencer. Two genotypes were not found in these datasets, and were added from another set:
GSGT Version 1.9.4; Processing Date 3/17/2015 9:51 AM; HumanOmniExpress-24-v1-0-B.bpm; Num SNPs 716503; request paddy_analysis.zip for check

MD150-070 01-0103 rs2268495 A-A

MD150-149 02-0040 rs6770013 A-C
