# Supplementary material for: A survival of the fittest strategy for the selection of genotypes by which drug responders and non-responders can be predicted in small groups
Source: PLoS One. 2021 Mar 5;16(3):e0246828. doi: 10.1371/journal.pone.0246828 (PMC7935233; doi:10.1371/journal.pone.0246828)

Er is geen verschil tussen zfactor op basis van gesplitste sets (derivatie en validatie) of over de gehele sample.

ROCs nu op basis van zfactor op gesplitste sets. Is dit theoretisch het best te verdedigen? Je deriveert immers eerst, en derivatie toets je op alles (incl ROCs etc) dus moet je wel op de gesplitste zfactor hanteren. Misschien toch zfactor over gehele set wanneer ROC over gehele set wordt berekend? (scheelt een paar honderdste op AUC).

**LYBRIDO**

ROC derivatie (AUC 0.867)


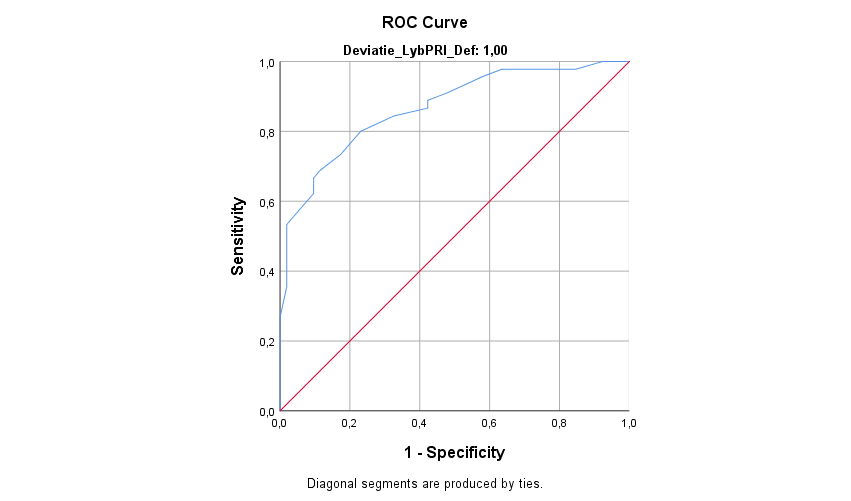


ROC validatie (AUC 0.890)


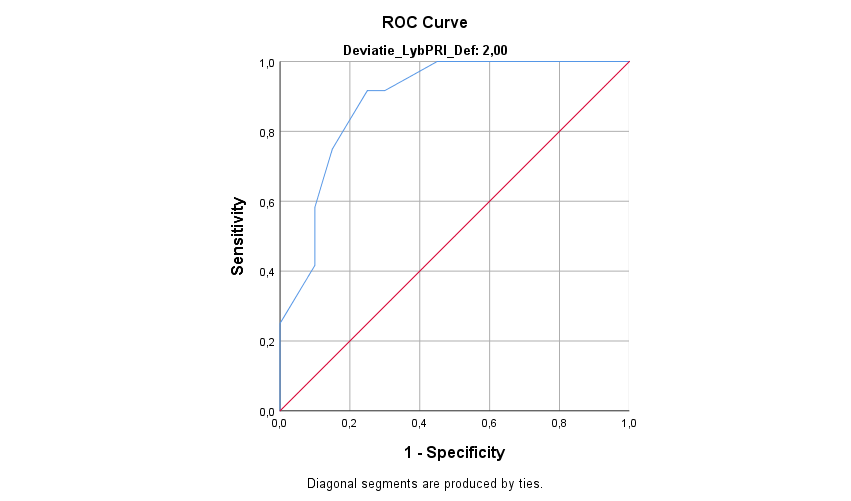


ROC geheel (AUC 0.868)


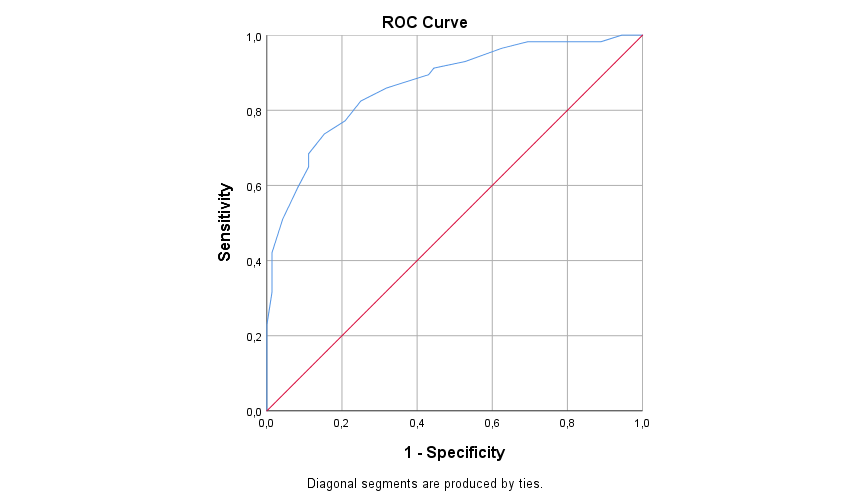


Crosstabs derivatie & validatie

| **PredLyb * LybResp Crosstabulation** | | | | | |
| --- | --- | --- | --- | --- | --- |
| Count | | | | | |
| Deviatie_LybPRI_Def | | | LybResp | | Total |
|  |  |  | ,00 | 1,00 |  |
| 1**,**00 | PredLyb | ,00 | 43 | 12 | 55 |
|  |  | 1,00 | 9 | 33 | 42 |
|  | Total | | 52 | 45 | 97 |
| 2,00 | PredLyb | ,00 | 14 | 1 | 15 |
|  |  | 1,00 | 6 | 11 | 17 |
|  | Total | | 20 | 12 | 32 |

Derivatie:

| Sensitivity | 0,733333 |
| --- | --- |
| Specificity | 0,826923 |
| FNR | 0,266667 |
| FPR | 0,173077 |
| PPV | 0,785714 |
| NPV | 0,781818 |
| Accuracy | 0,783505 |

Validatie:

| Sensitivity | 0,916667 |
| --- | --- |
| Specificity | 0,7 |
| FNR | 0,083333 |
| FPR | 0,3 |
| PPV | 0,647059 |
| NPV | 0,933333 |
| Accuracy | 0,78125 |

Crosstabs geheel

| **PredLyb * LybResp Crosstabulation** | | | | |
| --- | --- | --- | --- | --- |
| Count | | | | |
|  | | LybResp | | Total |
|  |  | ,00 | 1,00 |  |
| PredLyb | ,00 | 57 | 13 | 70 |
|  | 1,00 | 15 | 44 | 59 |
| Total | | 72 | 57 | 129 |

| Sensitivity | 0,77193 |
| --- | --- |
| Specificity | 0,791667 |
| FNR | 0,22807 |
| FPR | 0,208333 |
| PPV | 0,745763 |
| NPV | 0,814286 |
| Accuracy | 0,782946 |

ANOVA derivatie en validatie sets (afh var = SSE; onafh var = voorspelde groep)

| **ANOVA** | | | | | | |
| --- | --- | --- | --- | --- | --- | --- |
| Ver_LybPRI.SatisfiedEvents | | | | | | |
| Deviatie_LybPRI_Def | | Sum of Squares | df | Mean Square | F | Sig. |
| 1,00 | Between Groups | 216,687 | 1 | 216,687 | 24,273 | ,000 |
|  | Within Groups | 848,055 | 95 | 8,927 |  |  |
|  | Total | 1064,742 | 96 |  |  |  |
| 2,00 | Between Groups | 98,384 | 1 | 98,384 | 12,017 | ,002 |
|  | Within Groups | 245,616 | 30 | 8,187 |  |  |
|  | Total | 344,000 | 31 |  |  |  |


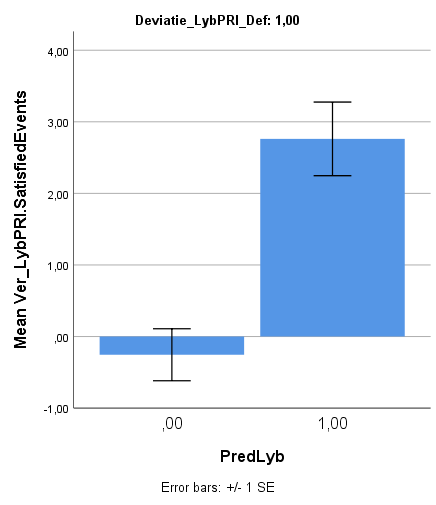


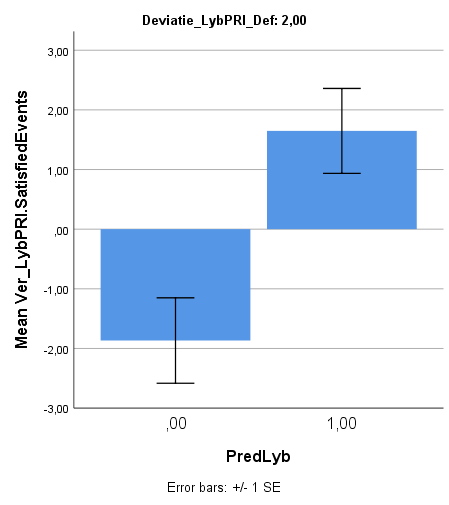


ANOVA gehele sets (afh var = SSE; onafh var = voorspelde groep)

| **ANOVA** | | | | | |
| --- | --- | --- | --- | --- | --- |
| Ver_LybPRI.SatisfiedEvents | | | | | |
|  | Sum of Squares | df | Mean Square | F | Sig. |
| Between Groups | 296,006 | 1 | 296,006 | 32,995 | ,000 |
| Within Groups | 1139,342 | 127 | 8,971 |  |  |
| Total | 1435,349 | 128 |  |  |  |


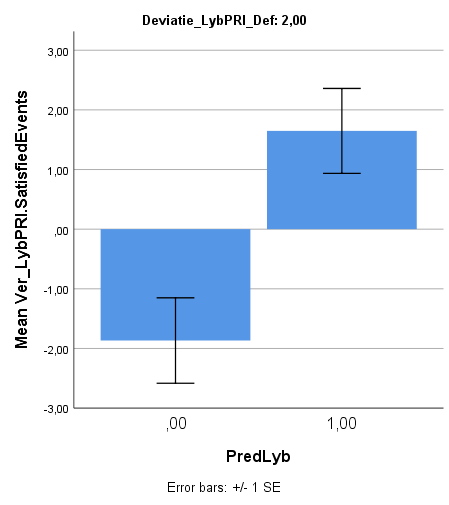

Supplement: S1 Data — (ZIP) [file pone.0246828.s004.zip › ROC analyses and figures/results samenvatting Lybrido.docx]
