# Supplementary material for: A survival of the fittest strategy for the selection of genotypes by which drug responders and non-responders can be predicted in small groups
Source: PLoS One. 2021 Mar 5;16(3):e0246828. doi: 10.1371/journal.pone.0246828 (PMC7935233; doi:10.1371/journal.pone.0246828)

**LYBRIDOS**

ROC derivatie (AUC 0.957)


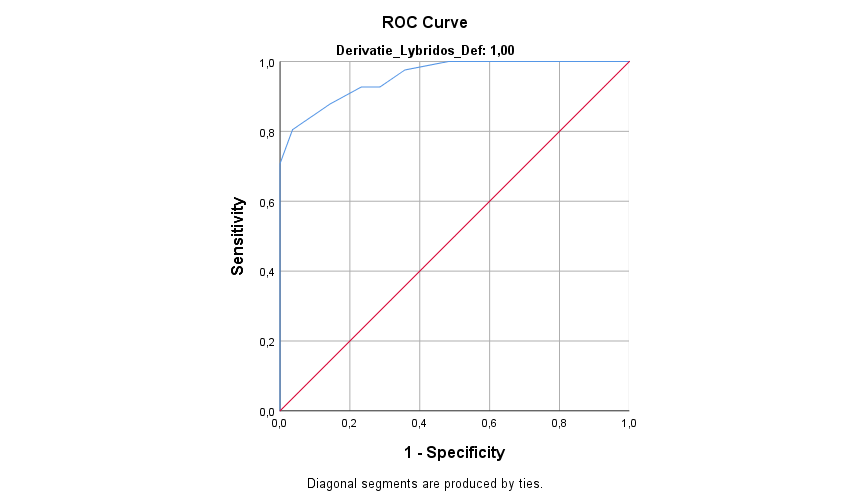


ROC validatie (AUC 0.869)


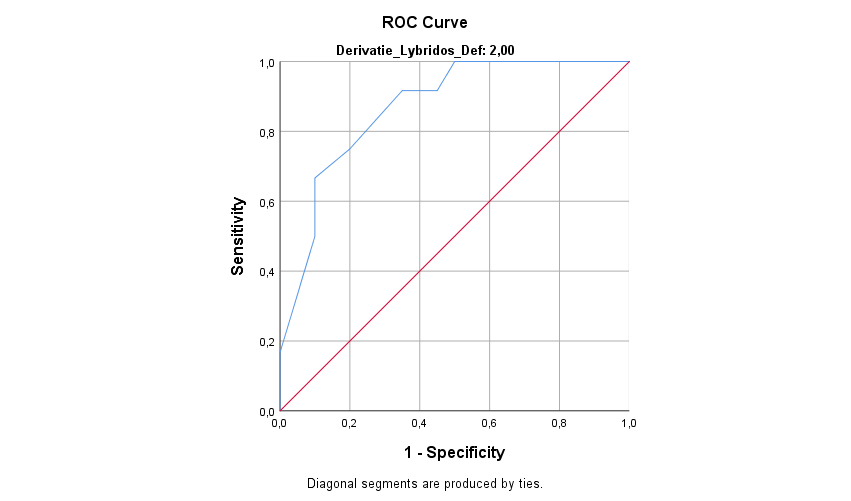


ROC geheel (AUC 0.933)


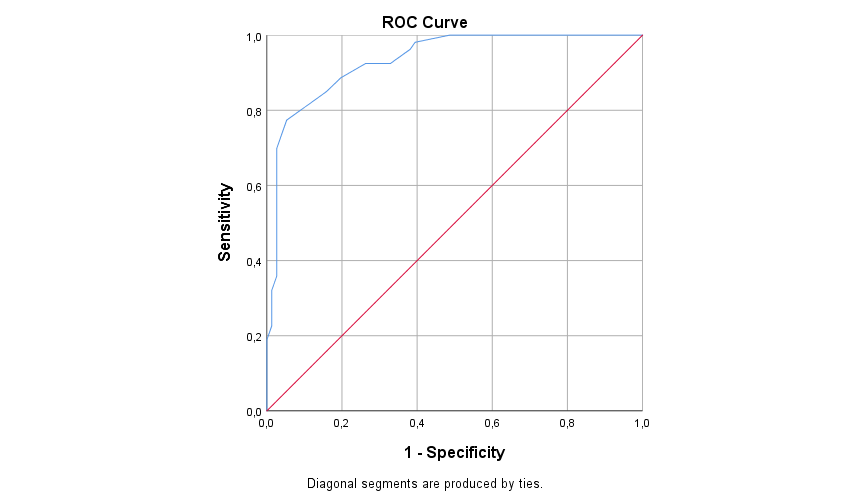


Crosstabs derivatie & validatie

| **PredLybs * LybsResp Crosstabulation** | | | | | |
| --- | --- | --- | --- | --- | --- |
| Count | | | | | |
| Derivatie_Lybridos_Def | | | LybsResp | | Total |
|  |  |  | ,00 | 1,00 |  |
| 1,00 | PredLybs | ,00 | 43 | 3 | 46 |
|  |  | 1,00 | 13 | 38 | 51 |
|  | Total | | 56 | 41 | 97 |
| 2,00 | PredLybs | ,00 | 13 | 1 | 14 |
|  |  | 1,00 | 7 | 11 | 18 |
|  | Total | | 20 | 12 | 32 |

Derivatie:

| Sensitivity | 0,926829 |
| --- | --- |
| Specificity | 0,767857 |
| FNR | 0,073171 |
| FPR | 0,232143 |
| PPV | 0,745098 |
| NPV | 0,934783 |
| Accuracy | 0,835052 |

Validatie:

| Sensitivity | 0,916667 |
| --- | --- |
| Specificity | 0,65 |
| FNR | 0,083333 |
| FPR | 0,35 |
| PPV | 0,611111 |
| NPV | 0,928571 |
| Accuracy | 0,75 |

Crosstabs geheel

| **PredLybs * LybsResp Crosstabulation** | | | | |
| --- | --- | --- | --- | --- |
| Count | | | | |
|  | | LybsResp | | Total |
|  |  | ,00 | 1,00 |  |
| PredLybs | ,00 | 56 | 4 | 60 |
|  | 1,00 | 20 | 49 | 69 |
| Total | | 76 | 53 | 129 |

| Sensitivity | 0,924528 |
| --- | --- |
| Specificity | 0,736842 |
| FNR | 0,075472 |
| FPR | 0,263158 |
| PPV | 0,710145 |
| NPV | 0,933333 |
| Accuracy | 0,813953 |

ANOVA derivatie en validatie sets (afh var = SSE; onafh var = voorspelde groep)

| **ANOVA** | | | | | | |
| --- | --- | --- | --- | --- | --- | --- |
| Ver_LybsPRI.SE | | | | | | |
| Derivatie_Lybridos_Def | | Sum of Squares | df | Mean Square | F | Sig. |
| 1,00 | Between Groups | 307,564 | 1 | 307,564 | 42,177 | ,000 |
|  | Within Groups | 692,766 | 95 | 7,292 |  |  |
|  | Total | 1000,330 | 96 |  |  |  |
| 2,00 | Between Groups | 104,960 | 1 | 104,960 | 12,983 | ,001 |
|  | Within Groups | 242,540 | 30 | 8,085 |  |  |
|  | Total | 347,500 | 31 |  |  |  |


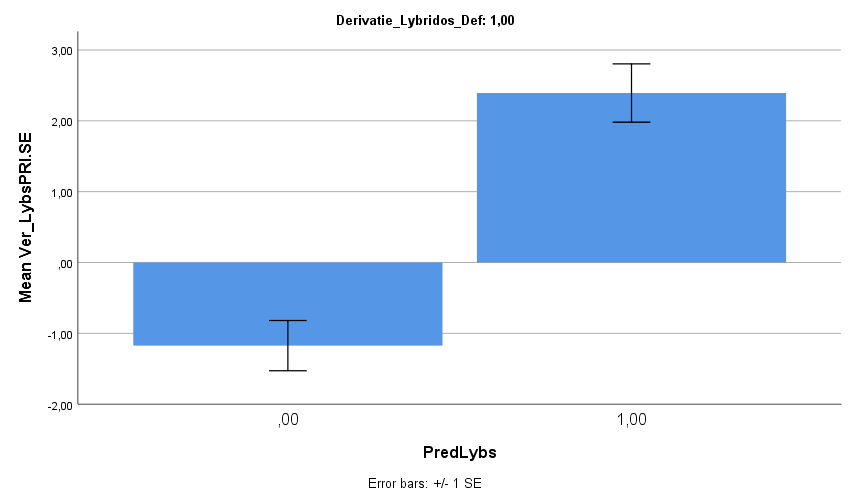


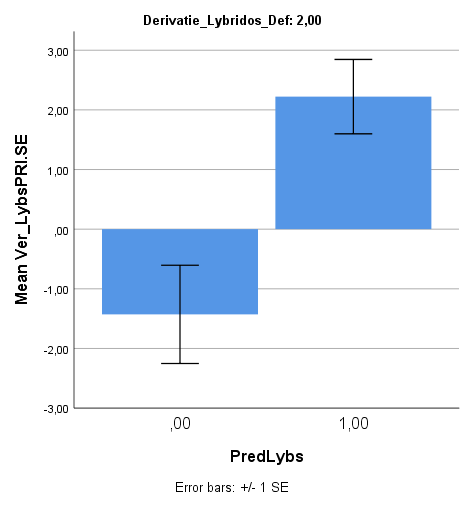


ANOVA gehele sets (afh var = SSE; onafh var = voorspelde groep)

| **ANOVA** | | | | | |
| --- | --- | --- | --- | --- | --- |
| Ver_LybsPRI.SE | | | | | |
|  | Sum of Squares | df | Mean Square | F | Sig. |
| Between Groups | 411,583 | 1 | 411,583 | 55,822 | ,000 |
| Within Groups | 936,386 | 127 | 7,373 |  |  |
| Total | 1347,969 | 128 |  |  |  |


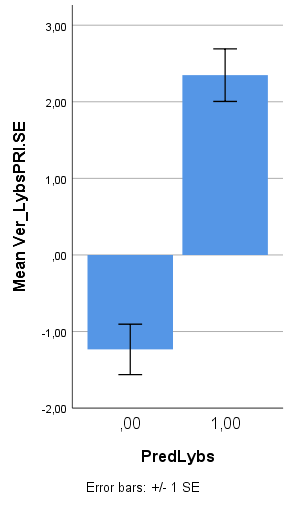

Supplement: S1 Data — (ZIP) [file pone.0246828.s004.zip › ROC analyses and figures/results samenvatting Lybridos.docx]
