# Supplementary figures and images for: A survival of the fittest strategy for the selection of genotypes by which drug responders and non-responders can be predicted in small groups
Source: PLoS One. 2021 Mar 5;16(3):e0246828. doi: 10.1371/journal.pone.0246828 (PMC7935233; doi:10.1371/journal.pone.0246828)

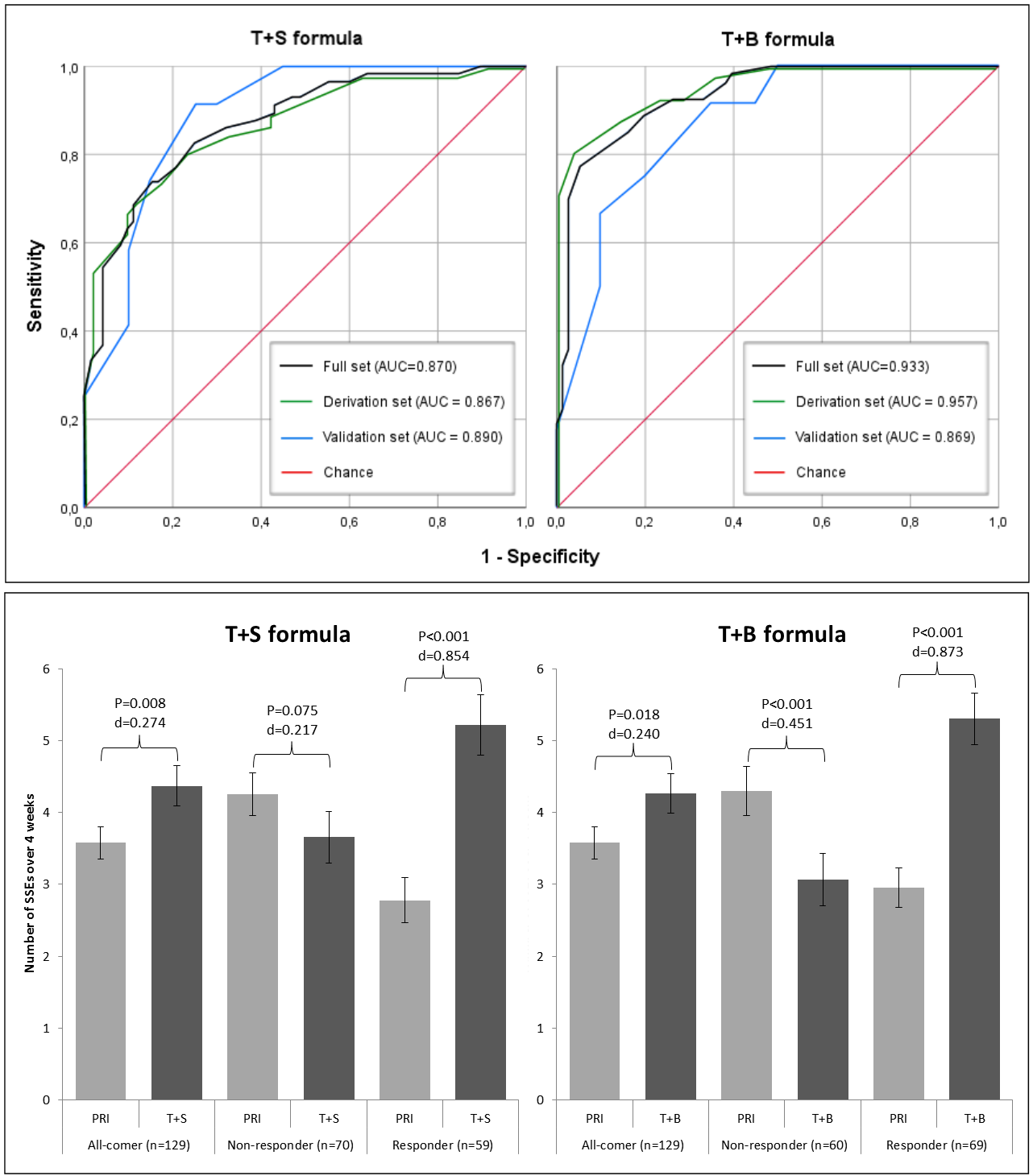

Supplement: S1 Data — (ZIP) [file pone.0246828.s004.zip › ROC analyses and figures/Corel Automatisch behouden/Resuts ROC SSE.tif]

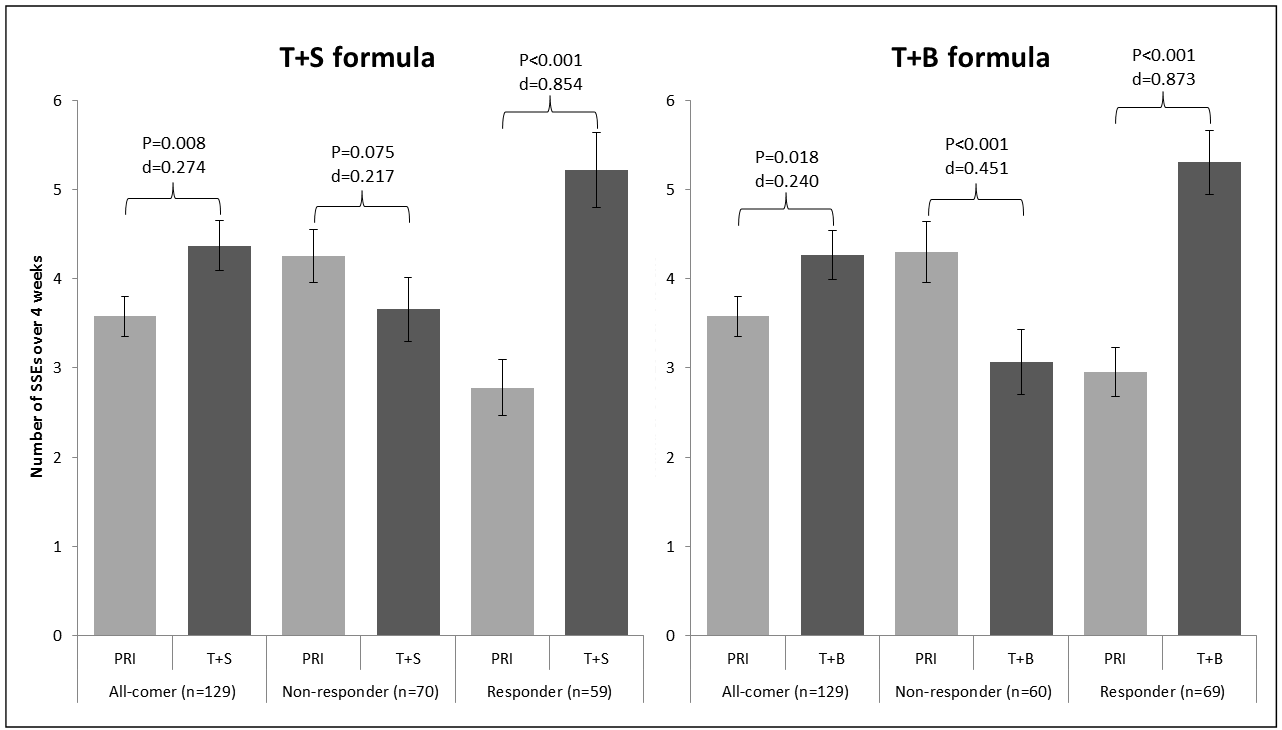

Supplement: S1 Data — (ZIP) [file pone.0246828.s004.zip › ROC analyses and figures/fig SSE Lyb(s).tif]

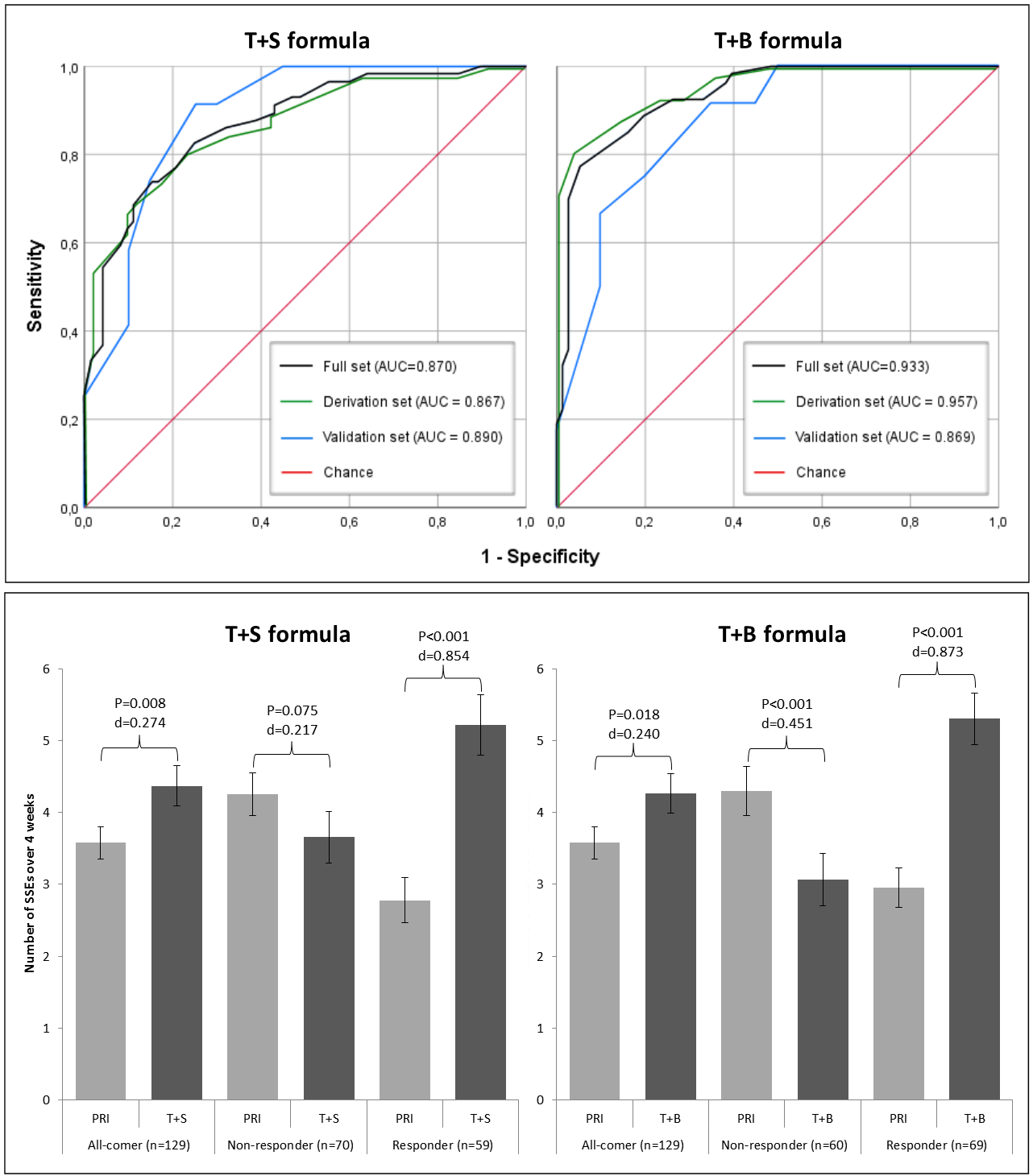

Supplement: S1 Data — (ZIP) [file pone.0246828.s004.zip › ROC analyses and figures/Resuts ROC SSE.tif]

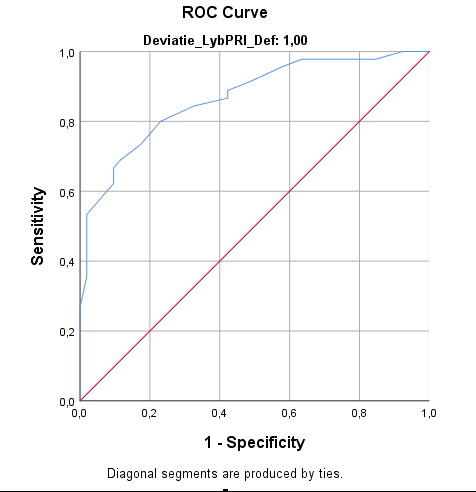

Supplement: S1 Data — (ZIP) [file pone.0246828.s004.zip › ROC analyses and figures/ROC LYB derivation.PNG]

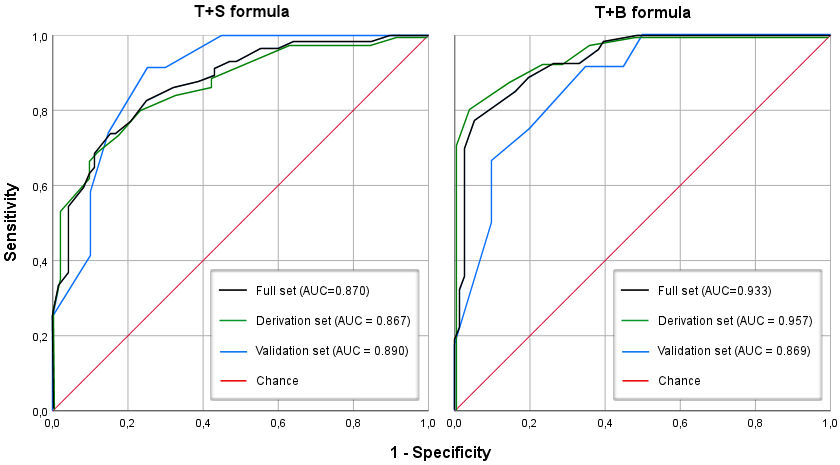

Supplement: S1 Data — (ZIP) [file pone.0246828.s004.zip › ROC analyses and figures/ROC LYB LYBS.jpg]

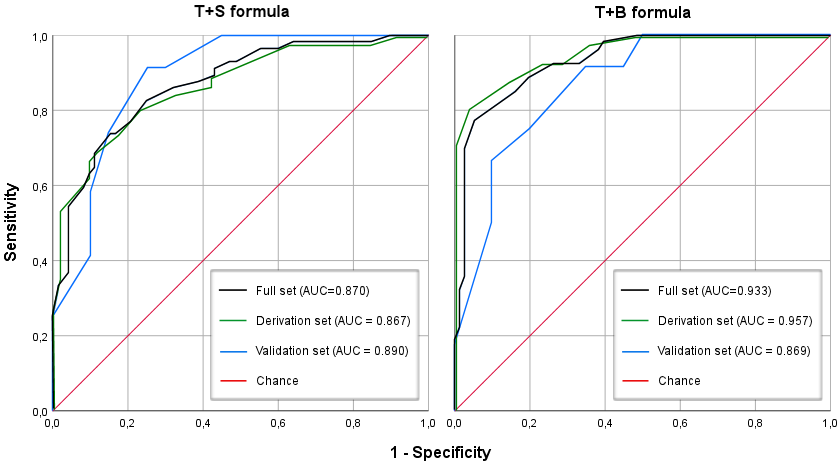

Supplement: S1 Data — (ZIP) [file pone.0246828.s004.zip › ROC analyses and figures/ROC LYB LYBS.tif]

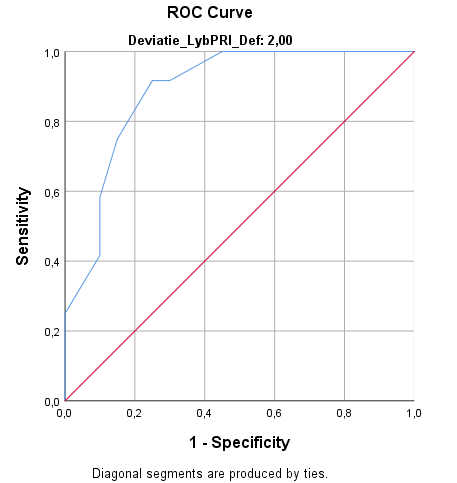

Supplement: S1 Data — (ZIP) [file pone.0246828.s004.zip › ROC analyses and figures/ROC LYB validation.PNG]

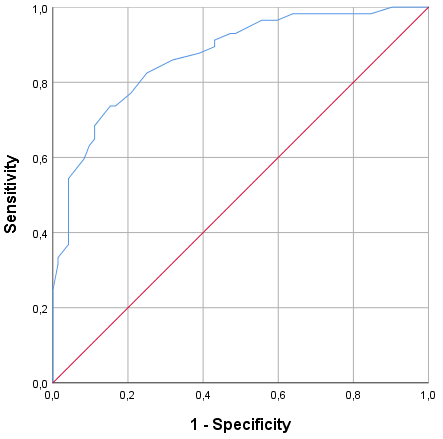

Supplement: S1 Data — (ZIP) [file pone.0246828.s004.zip › ROC analyses and figures/ROC Lyb.PNG]

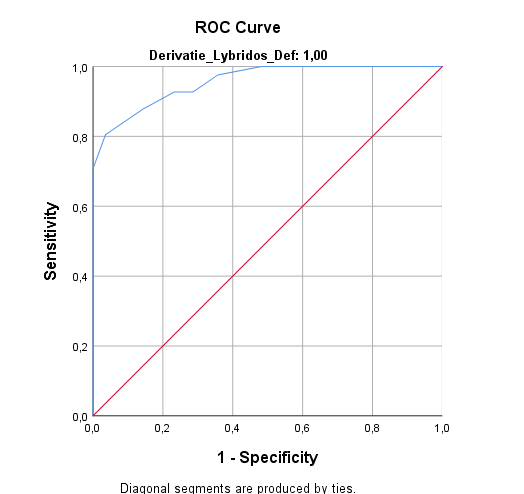

Supplement: S1 Data — (ZIP) [file pone.0246828.s004.zip › ROC analyses and figures/ROC LYBS derivation.PNG]

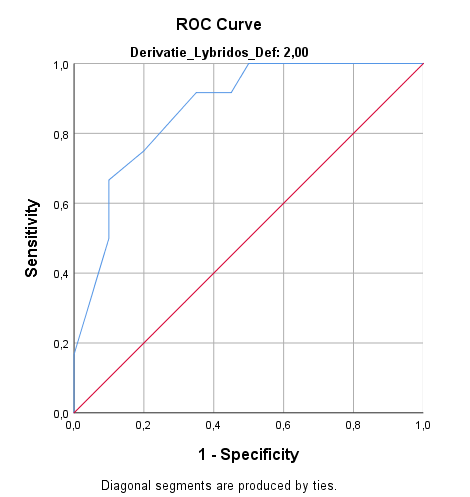

Supplement: S1 Data — (ZIP) [file pone.0246828.s004.zip › ROC analyses and figures/ROC LYBS validation.png]

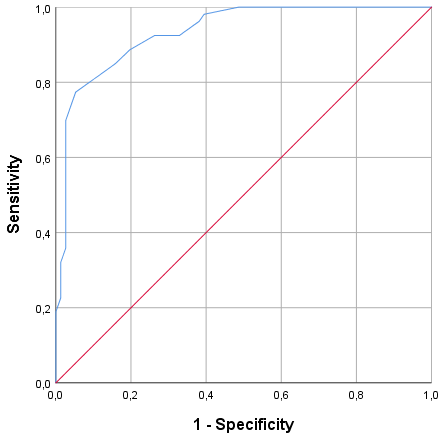

Supplement: S1 Data — (ZIP) [file pone.0246828.s004.zip › ROC analyses and figures/ROC Lybs.PNG]

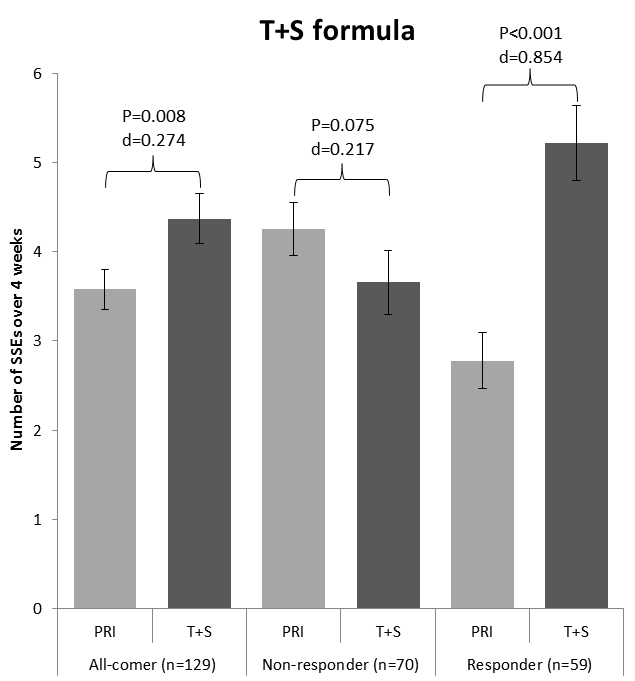

Supplement: S1 Data — (ZIP) [file pone.0246828.s004.zip › ROC analyses and figures/SSE LYB met all comers.PNG]

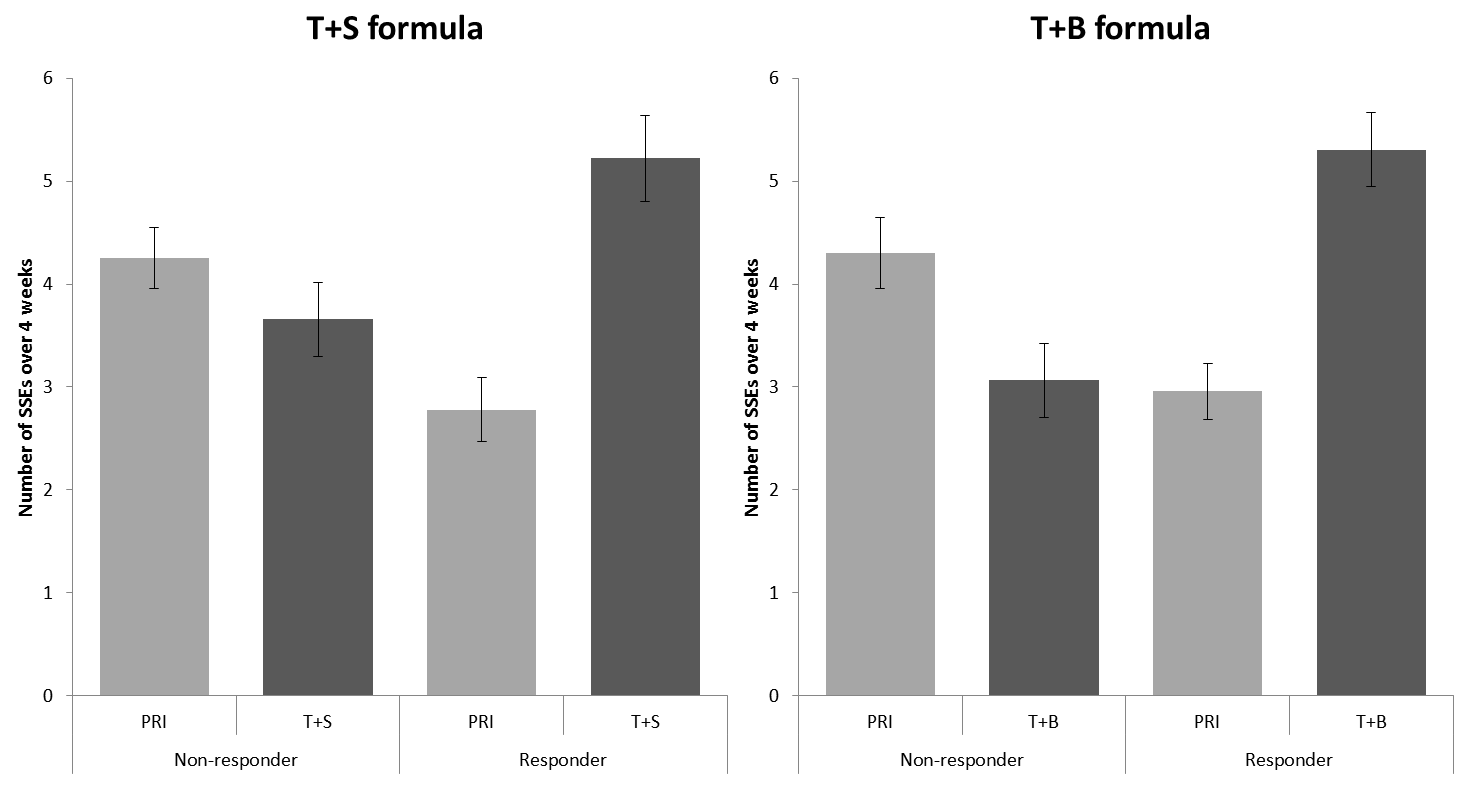

Supplement: S1 Data — (ZIP) [file pone.0246828.s004.zip › ROC analyses and figures/SSE LYB(S).tif]

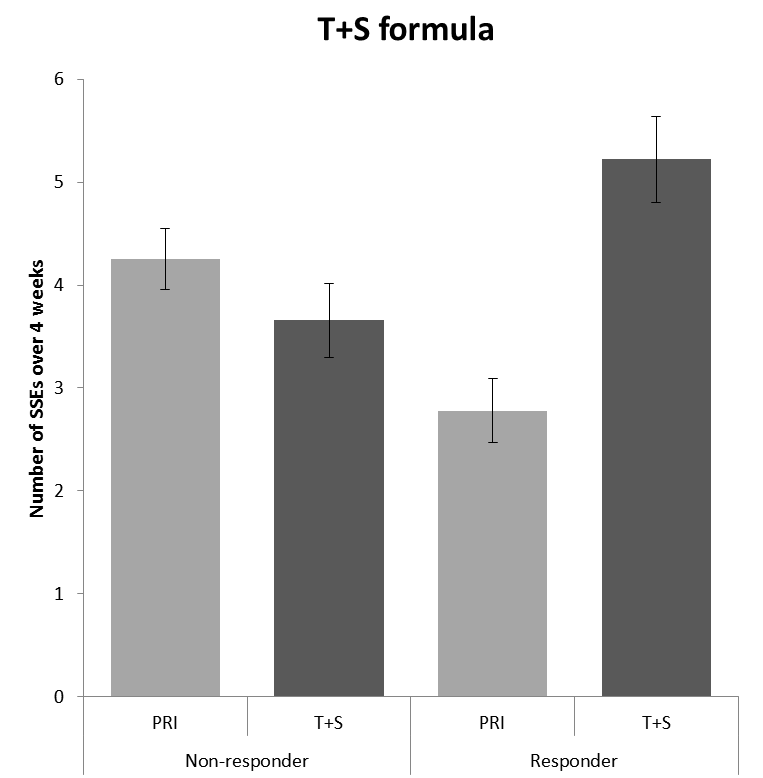

Supplement: S1 Data — (ZIP) [file pone.0246828.s004.zip › ROC analyses and figures/SSE LYB.PNG]

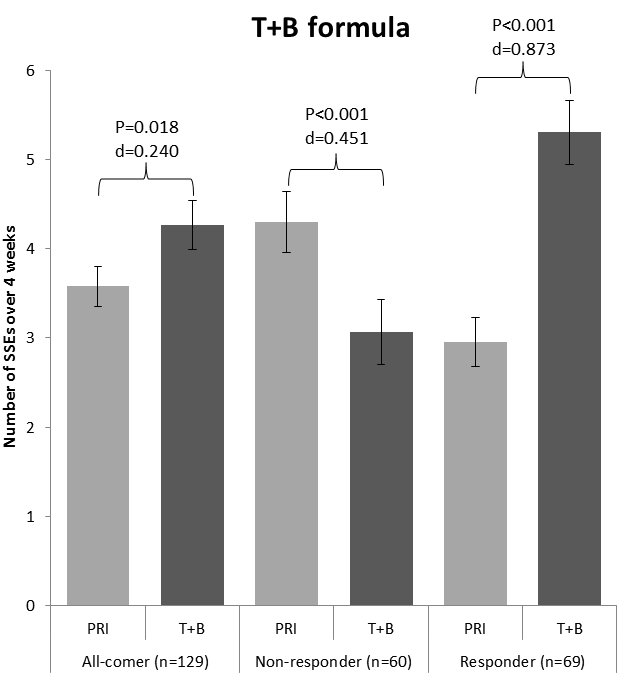

Supplement: S1 Data — (ZIP) [file pone.0246828.s004.zip › ROC analyses and figures/SSE LYBs met all comers.PNG]

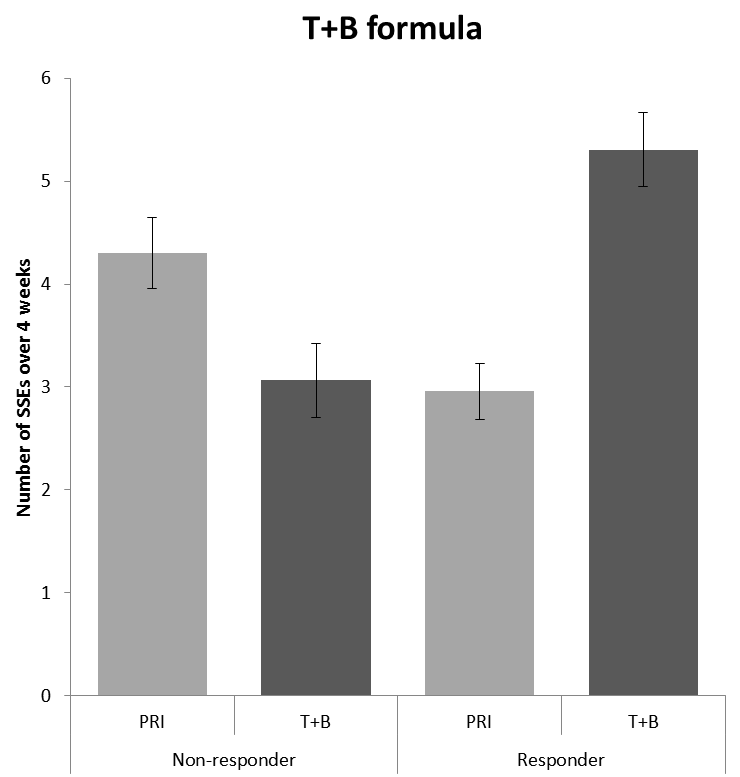

Supplement: S1 Data — (ZIP) [file pone.0246828.s004.zip › ROC analyses and figures/SSE LYBS.PNG]
